# Point and interval estimation in two-stage adaptive designs with time to event data and biomarker-driven subpopulation selection

**DOI:** 10.1002/sim.8557

**Published:** 2020-05-03

**Authors:** Peter K. Kimani, Susan Todd, Lindsay A. Renfro, Ekkehard Glimm, Josephine N. Khan, John A. Kairalla, Nigel Stallard

**Affiliations:** 1 Warwick Medical School, University of Warwick, Coventry, UK; 2 Department of Mathematics and Statistics, University of Reading, Reading, UK; 3 Division of Biostatistics, University of Southern California, Los Angeles, CA,; 4 Novartis Campus, Novartis Pharma AG, Basel, Switzerland; 5 MRC Biostatistics Unit, University of Cambridge, Cambridge, UK; 6 Department of Biostatistics, University of Florida, Gainesville, Florida,

**Keywords:** adaptive threshold design, enrichment designs, stratified medicine, subgroup analysis, survival data

## Abstract

In personalized medicine, it is often desired to determine if all patients or only a subset of them benefit from a treatment. We consider estimation in two-stage adaptive designs that in stage 1 recruit patients from the full population. In stage 2, patient recruitment is restricted to the part of the population, which, based on stage 1 data, benefits from the experimental treatment. Existing estimators, which adjust for using stage 1 data for selecting the part of the population from which stage 2 patients are recruited, as well as for the confirmatory analysis after stage 2, do not consider time to event patient outcomes. In this work, for time to event data, we have derived a new asymptotically unbiased estimator for the log hazard ratio and a new interval estimator with good coverage probabilities and probabilities that the upper bounds are below the true values. The estimators are appropriate for several selection rules that are based on a single or multiple biomarkers, which can be categorical or continuous.

## 1 ∣ INTRODUCTION

Clinical trials in personalized medicine involve assessing whether a patient’s characteristic(s), known as biomarkers, can be used to determine their best care. A biomarker may influence the progression of disease without treatment (prognostic biomarker) or the size of the effect of a treatment (predictive biomarker).^1^ We focus on predictive biomarkers, where the effects of a new treatment in different subpopulations defined by biomarker values are assessed. Several efficient two-stage adaptive designs with an interim analysis to determine the part of the population (subpopulation) to benefit most from a new treatment have been proposed.^2–6^ The general framework of such designs is that patients are recruited from the full population in stage 1, with an interim analysis performed to determine the subpopulation where the new treatment is apparently beneficial. In stage 2, patients are recruited from this group. Confirmatory analysis then includes data from both stages. Appropriate analysis of two-stage adaptive trials needs to adjust for the bias arising from using stage 1 data for both subpopulation selection and the final analysis.

Time to event patient outcomes are considered in several clinical trials assessing predictive biomarkers.^7–10^ For two-stage adaptive trials, methods for controlling type I error rate and/or increasing power have been developed.^2,3,7^ However, existing point estimators and confidence intervals that adjust for subpopulation selection do not consider time to event data.^4–6,11^ Li et al^12^ quantify the bias of the naive estimator for time to event data but do not derive unbiased estimators. Thus, there is a need to develop point and interval estimators for time to event data in two-stage adaptive trials with subpopulation selection. This is the aim of this article. Using the asymptotic distribution of the log hazard ratio, we extend existing methods for normally distributed data to time to event patient outcomes. We also address the additional complexity associated with following in stage 2 the stage 1 patients without the event of interest at the interim analysis.

For normally distributed outcomes, estimators that adjust for subpopulation selection may be obtained in three ways. The first involves estimating and subtracting the bias of the naive estimator.^6,13–15^ The second utilizes the empirical Bayes technique to obtain shrinkage estimators.^6,15–17^ The third is based on the Rao-Blackwell theorem that the expected value of the unbiased stage 2 estimator conditional on the selected subpopulation and a sufficient statistic is the uniformly minimum variance conditional unbiased estimator (UMVCUE).^4,6,18–22^ Kimani et al^6^ compared the estimation approaches in the context of subpopulation selection, concluding that the UMVCUE was superior. As we expect the same conclusion if the three estimators are extended to time to event data, in this article, we have only extended the UMVCUE. To address the complexity associated with time to event data, we assume hypothesis testing similar to that proposed by Jenkins et al^3^ and use the duality with hypothesis testing to construct confidence intervals with desired properties as proposed for non-time to event data by Magirr et al.^23^

Previous research has considered biomarkers of various forms (a binary biomarker, a continuous biomarker, or multiple biomarkers) and different subpopulation selection rules. Our point and interval estimators are appropriate for different selection rules and biomarkers of many forms.

## 2 ∣ SETTING AND NOTATION

### 2.1 ∣ Partitioning the population and general concepts in selecting partitions

**FIGURE 1:**
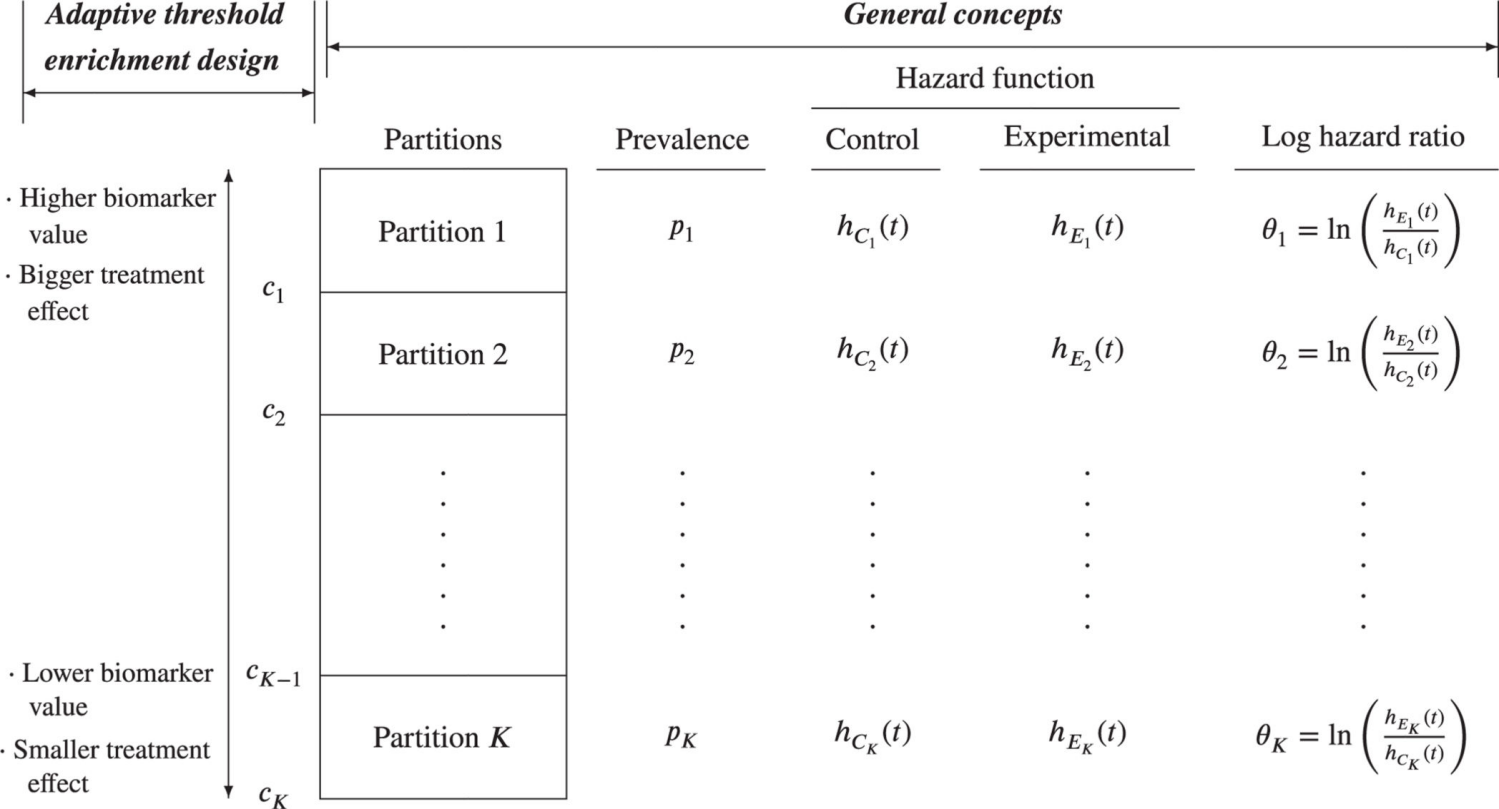
Partitioning of the full population

This section describes the partitioning of the full population (*F*) and general approaches for specifying selection rules. Specific selection rules will be described in Section 2.3. Figure 1 summarizes the partitioning of *F*. We first describe concepts common to all settings that we consider, indicated as general concepts in the figure. Assume that *F* consists of *K* (*K* ≥ 2) distinct partitions. For partition *j* (*j* = 1, … *, K*), we denote the true prevalence of patients by *p*_*j*_ and hazard functions for the control and experimental groups by $h_{C_{j}}(t)$ and $h_{E_{j}}(t)$, respectively. Assuming proportional hazards within a partition, we denote the log hazard ratio (HR) for partition *j* (*j* = 1, … *, K*) by *θ*_*j*_, with *θ*_*j*_ *<* 0 indicating that the experimental treatment delays occurrence of the event in partition *j* and hence is superior to the control.

Let S⊆{1,…,K} be the subset of indices corresponding to the partitions selected to continue to stage 2. The partitions are selected based on the stage 1 estimate for ${\left(\theta_{1},\ldots ,\theta_{K}\right)}^{\prime }$. At the end of the trial, for each j∈S, the aim is to estimate *θ*_*j*_. We will obtain log HR estimates in selected partitions separately, corresponding to a stratified model. Consequently, although the selection rules we consider in this paper are aimed at identifying predictive biomarkers, as shown in Figure 1, control group hazard functions in different partitions may be different so that the biomarker may also be prognostic. A disadvantage of this approach is that in some cases, such as when the biomarker is neither predictive nor prognostic, it would be better to obtain a single estimate using the data from all the partitions while assuming proportional hazards overall in *F* rather than separate estimates of effects assuming proportional hazards only within a partition. A model with partition membership as a categorical covariate and an interaction term for partition and treatment would enable an estimator of a combined effect. However, this model is not as general as the stratified model, imposing more restrictions on the hazard functions.

The expected relationship between biomarker and treatment effect informs the partitioning of *F* and the selection rule. Figure 1 gives an example of the two-stage adaptive threshold enrichment design.^6,24^ Here, it is assumed that a single continuous biomarker and the treatment effect are monotonically related with higher biomarker values associated with bigger treatment effects. Consequently *K* candidate threshold values *c*_1_ *> c*_2_ *>* … *> c*_*K*_ are specified to subdivide *F* into *K* distinct partitions. Setting *c*_0_ = ∞, partition *j*(*j* = 1, … *, K*) consists of patients with biomarker values in [*c*_*j*_*, c*_*j*–1_]. As it is expected that *θ*_1_ ≤ *θ*_2_ ≤ ⋅ ⋅ ⋅ ≤ *θ*_*K*_, a selection rule is prespecified to test partitions in stage 2 with biomarker values above *c*_*s*_ (*s* ∈ {1, … *, K*}).

Partitioning of *F* and selection rules can be similarly given for biomarkers of other forms. Common cases are a single binary biomarker and multiple biomarkers. A single binary biomarker where the effect in one partition is expected to be bigger than in the complementary partition is a special case of the continuous biomarker with *K* = 2. For multiple biomarkers, we consider two scenarios. In the first, we assume biomarkers’ values can be combined into an aggregate score with a monotonic relationship with the treatment effect, with this score used to define partitions and a selection rule as for a single continuous biomarker. In the second scenario, the partitions consist of different combinations of biomarker level categories. A monotonic relationship between the biomarker values and treatment effects is not assumed and so a selection rule where partition *j*(*j* = 1, … *,K*) is considered for continuing to stage 2 based on the stage 1 estimate for *θ*_*j*_ only is specified. A single binary biomarker, where there is no knowledge of the partition that is more likely to benefit, can be considered a special case of the second scenario with *K* = 2.

### 2.2 ∣ Analysis times and notation of estimates for different subsets of trial data

**FIGURE 2:**
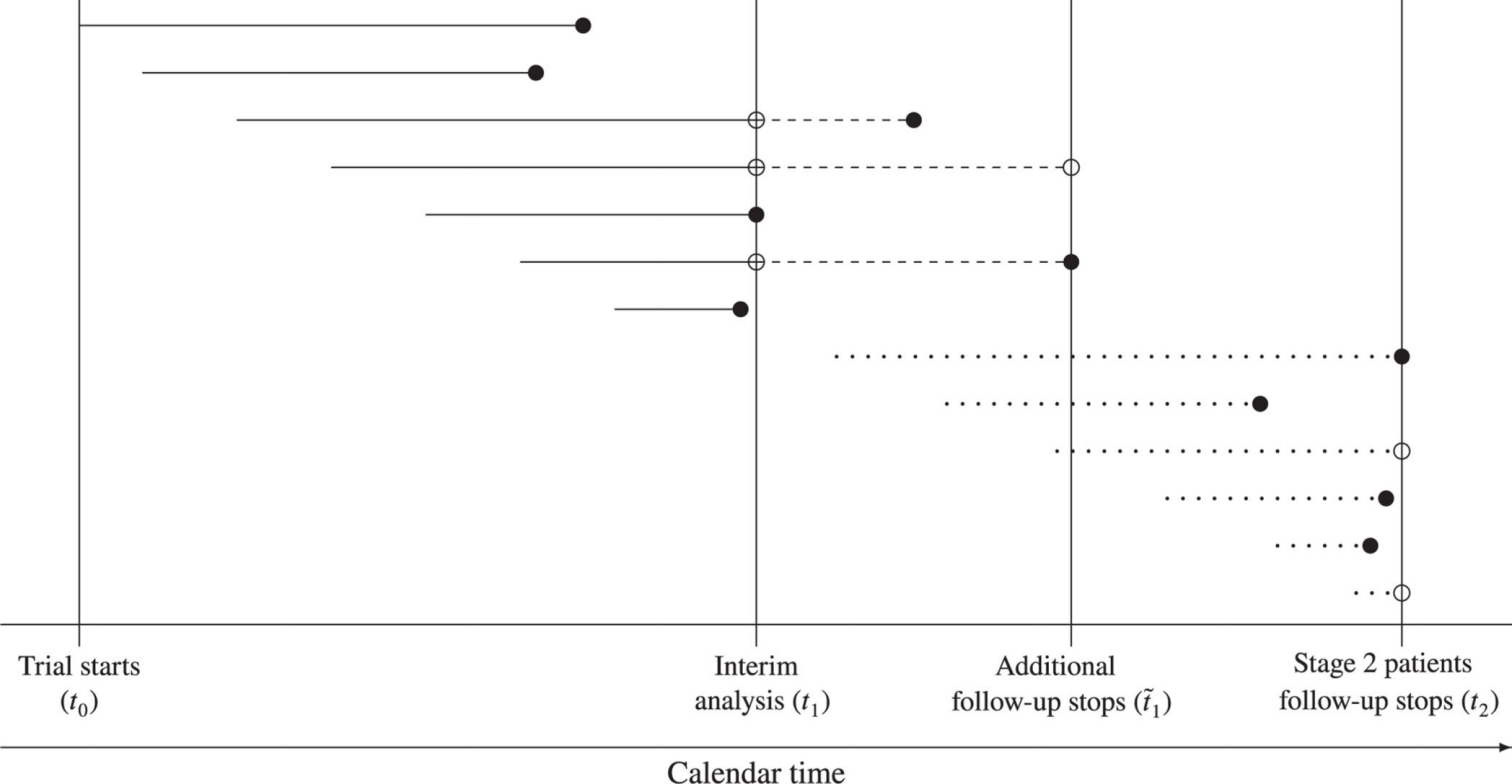
Various time points in the adaptive design with time to event data

Figure 2 shows the available data at different times in the trial. Each horizontal line that ends with a circle corresponds to a patient, with the line’s length being the patient’s survival time in the trial. The left hand end of each line corresponds to the calendar time a patient was recruited. Filled and non-filled circles correspond to an event having occurred and not, respectively.

The trial starts recruiting at some time *t*_0_ and an interim analysis is performed at time *t*_1_. In Sections 4 and 5, we will take *t*_1_ to correspond to when a prespecified number of events is observed. Alternatives include *t*_1_ being a prespecified date.^3,25^ Stage 1 consists of the data that are used in the interim analysis, with the survival times being the lengths of continuous lines in Figure 2. As described below, we obtain estimates from these data based on the distribution of the score statistic. Estimates based on the distribution of the score statistic are similar to those from the Cox’s proportional hazards model.^26^ The choice of the model used to obtain estimates is discussed in Section 6. Let *S*_1,*j*_ and *V*_1,*j*_ (*j* = 1, … *,K*) be the score statistic and Fisher information, respectively, obtained from analyzing partition *j* stage 1 data at *t*_1_. Based on the score statistic theory, asymptotically $S_{1,j}\sim N\left(\theta_{j}V_{1,j},V_{1,j}\right)$. Note that the estimator ${\hat{\theta }}_{1,j}$ defined by $S_{1,j}/V_{1,j}\text{ is }N\left(\theta_{j},\sigma_{1,j}^{2}\right)$, where $\sigma_{1,j}^{2}=1/V_{1,j}$ (for example, see chapter 3 ofWhitehead^27^ and chapter 13.4 of Jennnison and Turnbull^28^).

Based on the stage 1 observed value for the vector ${\left({\hat{\theta }}_{1,1},\ldots ,{\hat{\theta }}_{1,K}\right)}^{\prime }$, the trial stops for futility or continues to stage 2 with *F* or some part of *F*. Various selection rules are described in Section 2.3. Stage 2 patients are recruited only from the selected partitions. Recruitment and follow-up of stage 2 patients stops at calendar time *t*_2_. In Sections 4 and 5, we take *t*_2_ to correspond to when a prespecified number of events from stage 2 patients is observed but alternatives such as *t*_2_ being a prespecified date can be used. In Figure 2, the survival times of stage 2 patients correspond to the lengths of the dotted lines.

At the interim analysis, some stage 1 patients will not have had the event of interest. As following these patients further gives estimators with smaller standard errors, we assume that they are followed up to time ${\tilde{t}}_{1}$. The choice of ${\tilde{t}}_{1}$ is described below. We refer to the data collected from stage 1 patients after the interim analysis as the incremental data.

For j∈S, let $S_{N_{j}}$ and $V_{N_{j}}$ denote the score statistic and Fisher information obtained from all patients recruited in partition *j* with the survival times and status for stages 1 and 2 patients determined at times ${\tilde{t}}_{1}$ and *t*_2_, respectively. Similar to above, ${\hat{\theta }}_{N_{j}}$ defined by $S_{N_{j}}/V_{N_{j}}$. is asymptotically $N\left(\theta_{j},\sigma_{N_{j}}^{2}\right)$, where $\sigma_{N_{j}}^{2}=1/V_{N_{j}}$. In the next paragraph, we will describe a strategy for achieving approximate independence between data collected before and after the interim analysis. When independence can be assumed, score statistic theory has been extended to a setting with repeated analyses of data, such as analyzing all patients’ data at *t*_l_ and *t*_2_.^26,29^ This gives $S_{N_{j}}-S_{1,j}$ independent of $S_{1,j}$ and asymptotically $N\left(\theta_{j}\left(V_{N_{j}}-V_{1,j}\right),V_{N_{j}}-V_{1,j}\right)$.^26,29,30^ It follows that ${\hat{\theta }}_{2,j}$ defined as $\left(S_{N_{j}}-S_{1,j}\right)/\left(V_{N_{j}}-V_{1,j}\right)$ is $N\left(\theta_{j},\sigma_{2,j}^{2}\right)$, where $\sigma_{2j}^{2}=1/\left(V_{N_{j}}-V_{1,j}\right)$. Note that

(1) $${\hat{\theta }}_{N_{j}}=\frac{\sigma_{2,j}^{2}{\hat{\theta }}_{1,j}+\sigma_{1,j}^{2}{\hat{\theta }}_{2,j}}{\sigma_{1,j}^{2}+\sigma_{2,j}^{2}}.$$

Estimators developed in Section 3 require $S_{N_{j}}-S_{1,j}$ to be independent of *S*_1,*j*_ (the independent increment structure), where test statistics based on the data from before and after the interim analysis are independent.^3,31,32^ However, adaptation such as subpopulation selection may induce correlation.^33^ If, as we propose above, *t*_*i*_ (*i* = 1, 2) depends on stage *i* patients only, conditional on the selection made, stage 2 patients’ data are independent of *S*_1,*j*_ and so any correlation between $S_{N_{j}}-S_{1,j}$ and $S_{1,j}$is assumed to be induced by the stage 1 patients’ incremental data. Some authors ignore this correlation noting that the independent increment structure assumption holds approximately. We follow Jenkins et al^3^ who, in addition to setting *t*_1_ and *t*_2_ independent of each other, for example, as described above, suggest improving the independent increment structure assumption by fixing in advance the rule for how long the stage 1 patients without events of interest at *t*_1_ are followed post stage 1. This ensures independence of Fisher information for stage 1, stage 2, and the incremental data. We suggest two rules for fixing the length of post stage 1 follow-up, and hence ${\tilde{t}}_{1}$. These rules are valid when *t*_1_ and *t*_2_ are determined as above, that is independently, and they (*t*_1_ and *t*_2_) are either prespecified dates, correspond to observation of prespecified numbers of events or are based on any other method for prespecifying duration of trials with time-to-event data.^3,25^ In the first, a fixed time between *t*_1_ and ${\tilde{t}}_{1}$ is prespecified. This rule achieves approximate independence for whether or not stage 1 patients from the dropped partitions without events at *t*_1_ are followed until ${\tilde{t}}_{1}$, though this should be specified before the trial and also, they should only be followed as part of the trial if they continue with the allocated treatments and adhere to the trial protocol. In the second rule, ${\tilde{t}}_{1}$ is the time when a prespecified number of events from stage 1 patients without events at *t*_1_ is obtained. For the approximate independence to work well, this rule requires that the patients from dropped partitions are followed until ${\tilde{t}}_{1}$. Therefore, we only recommend this rule if it is plausible for the stage 1 patients from the dropped partitions to continue with the allocated treatments and adhere to the trial protocol. In Sections 4 and 5, we used the first rule. To assess the approximate independence assumption with this approach, we computed correlations between ${\hat{\theta }}_{1,j}$ and ${\hat{\theta }}_{2,j}$ for some scenarios in Section 5 (not presented) and obtained similarly small values as Tsiatis et al.^29^ Note that if *t*_1_ and *t*_2_ correspond to prefixed dates, it is valid to set ${\tilde{t}}_{1}=t_{2}$.

In some cases, such as when *t*_2_ corresponds to the time when a prespecified number of events from stage 2 patients is obtained and ${\tilde{t}}_{1}$ is a prefixed date, it is possible to have ${\tilde{t}}_{1}>t_{2}$. In practice, this is undesirable since stage 2 patients’ follow-up information beyond *t*_2_ is not included in data analysis. Therefore, in practice, ${\tilde{t}}_{1}$should be fixed in such a way that ${\tilde{t}}_{1}>t_{2}$ is very unlikely.

In this section, we have made the assumption that, for $j(j=1,\ldots ,K),{\hat{\theta }}_{1,j}\sim N\left(\theta_{j},\sigma_{1,j}^{2}\right)$, and for each $j\in S,{\hat{\theta }}_{N_{j}}\sim N\left(\theta_{j},\sigma_{N_{j}}^{2}\right)$ and ${\hat{\theta }}_{2,j}\sim N\left(\theta_{j},\sigma_{2,j}^{2}\right)$.We emphasize that these distributional assumptions are conditional on the selection made.

For example, while deriving unbiased estimators in Section 3.1, we will adjust for subpopulation selection by taking the expectation over the region of the decision made based on the interim analysis results. Also, since there is no overlap of patients among the partitions, estimates from different partitions are independent. For example, for $j\neq j^{\prime },{\hat{\theta }}_{1,j}$ is independent of each of ${\hat{\theta }}_{1,j^{\prime }},{\hat{\theta }}_{N_{f}}$, and ${\hat{\theta }}_{2,j^{\prime }}$

### 2.3 ∣ Selection rules

**FIGURE 3:**
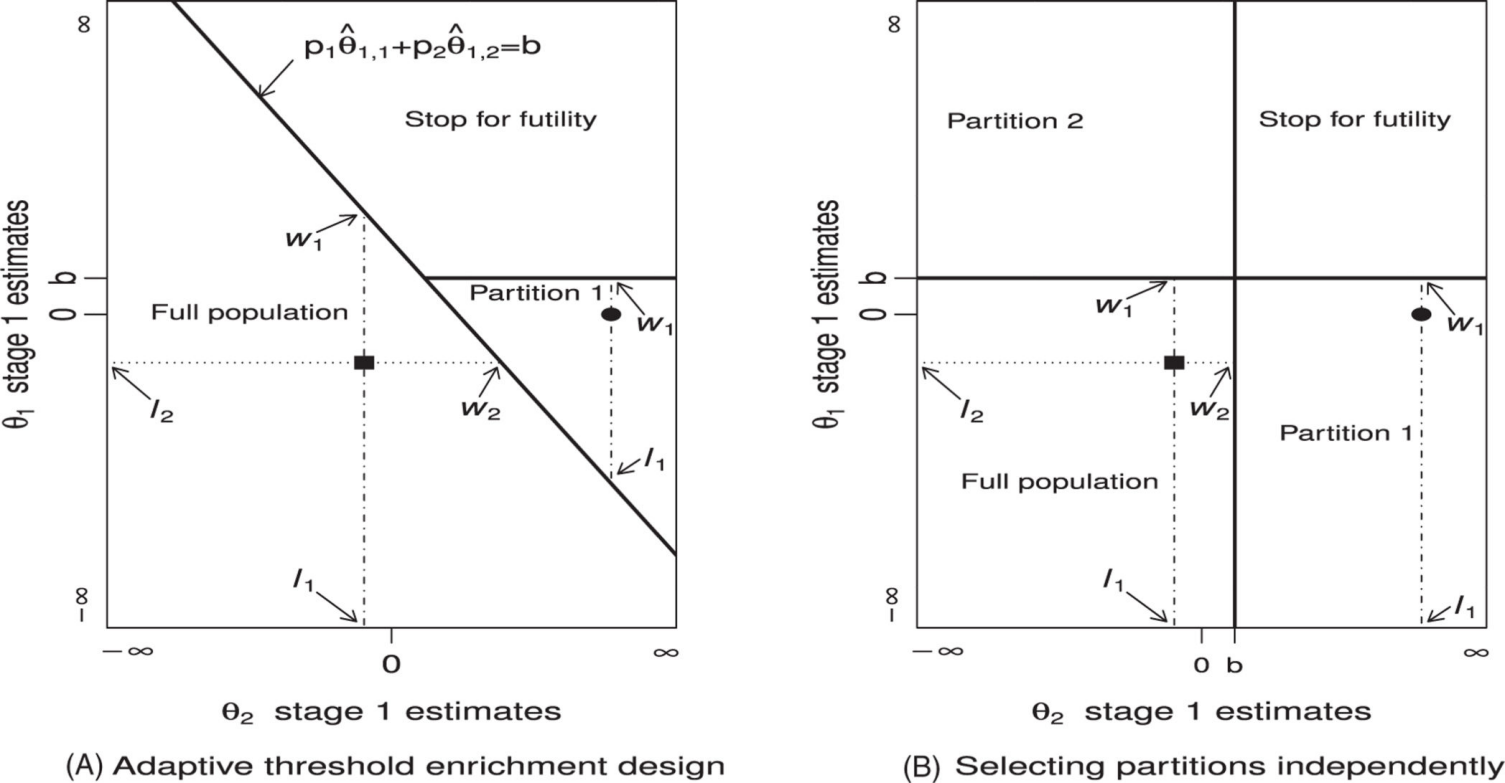
Decision regions for two selection rules when *K* = 2. The continuous lines are the decision boundaries. The filled circle and square are two possible stage 1 results that lead to selecting partition 1 and *F*, respectively. The edges of the vertical dashed and dotted lines give the bounds for estimating *θ*_1_ that are denoted by *l*_1_ (lower bound) and *w*_1_ (upper bound). The edges of the horizontal dashed lines give the bounds for estimating *θ*_2_ and are denoted by *l*_2_ (lower bound) and *w*_2_ (upper bound). A, Adaptive threshold enrichment design. B, Selecting partitions independently

Estimators proposed in this article can be used to adjust for any subpopulation selection rule based only on the stage 1 observed value for the vector ${\left({\hat{\theta }}_{1,1},\ldots ,{\hat{\theta }}_{1,K}\right)}^{\prime }$. In this section, we review selection rules suggested by various authors. The first is appropriate for the two-stage adaptive threshold enrichment design described in paragraph three of Section 2.1.^6,7^ Let *τ*_*s*_ denote the subpopulation consisting of partitions 1 to *s* and let $p_{j}^{\prime }=\sum_{i=1}^{j}p_{i}(j=1,\ldots ,K)$. To maximize the number of partitions tested in stage 2,we continue with the largest subpopulation *τ*_*s*_ (*s* = 1,…*, K*) such that $\sum_{j=1}^{s}p_{j}{\hat{\theta }}_{1,j}/p_{s}^{\prime }\leq b$, where *b* is a prespecified value. Note that although $\sum_{j=1}^{s}p_{j}{\hat{\theta }}_{1,j}/p_{s}^{\prime }$ is not interpretable, it can give an indication of the treatment effects in the *s* partitions included. Figure 3A shows the decision regions for this rule when *K* = 2 and *p*_1_ = *p*_2_. The filled square is an example of a case where both partitions would continue to stage 2, while the filled circle is an example of a case where only partition 1 would continue.

The selection rule just described is appropriate when a monotonic relationship between the biomarker and the treatment effect is expected. However, as described in Section 2.1, sometimes it is not expected that the relationship is monotonic. In such a case, for a binary biomarker, a selection rule should enable the trial to continue to stage 2 with partition 1 (biomarker +ve), partition 2 (biomarker -ve) or both (*F*). As described in Section 2.1, this can be extended to *K >* 2. A common selection rule in this setting is to decide whether partition *j* (*j* = 1, … *, K*) continues to stage 2 based on ${\hat{\theta }}_{1,j}$ only.^12^ Thus, with a futility boundary, partitions with stage 1 estimates below *b* continue to stage 2. The decision regions for this rule when *K* = 2 are shown in Figure 3B.

We will demonstrate the estimators developed in Section 3 with the above two selection rules. Other selection rules^4,5,12,34^ are reviewed in the supplementary material. For all selection rules, for some values of (*θ*_1_, … *,θ*_*K*_ ), even when *F* is selected, the naive estimates are biased because of subpopulation selection. The new estimators in Section 3 correct for this bias since they condition on the selection rule, the selected partitions, and the observed data. The estimators also correct for bias appropriately when the selection rule does not reflect the true underlying relationship between biomarker and treatment effect.

### 2.4 ∣ Naive estimation

We will consider ${\hat{\theta }}_{N_{j}}$, given by expression (1), as the naive point estimator. Note that ${\hat{\theta }}_{N_{j}}$ is not simply the estimate based on all data available at the end of the trial because, as described in Section 2.2, it is based on the data where the independent increment is assumed. For the special case of ${\tilde{t}}_{1}=t_{1}$, the bias of ${\hat{\theta }}_{N_{j}}$ corresponds to the subpopulation selection bias. The difference in biases for ${\hat{\theta }}_{N_{j}}$ computed at ${\tilde{t}}_{1}=t_{1}$ and ${\hat{\theta }}_{N_{j}}$ computed at ${\tilde{t}}_{1}=t_{1}$ gives an indication of the bias attributable to the incremental data.

For the naive confidence interval, we assume that for each j∈S, the naive estimator ${\hat{\theta }}_{N_{j}}\sim N\left(\theta_{j},\sigma_{N_{j}}^{2}\right)$. Consequently, for each j∈S, the two sided naive confidence interval for *θ*_*j*_ that splits *α* equally among the |S| selected partitions is

(2) $${\hat{\theta }}_{N_{j}}\pm z_{\alpha /(2|S|)}\sigma_{N_{j}},$$

where $z_{\alpha /(2|S|)}=\Phi^{-1}\{1-\alpha /(2|S|)\}$. This naive confidence interval addresses the issue of the independent increments as described in Section 2.2 and adjusts for multiple hypotheses but not the subpopulation selection.

## 3 ∣ BIAS ADJUSTED ESTIMATORS

### 3.1 ∣ New approximately conditionally unbiased point estimator

To adjust for the subpopulation selection, for each j∈S, we derive a UMVCUE for *θ*_*j*_. The UMVCUE is based on the Rao-Blackwell theorem, which was initially proposed in adaptive designs by Cohen and Sackrowitz^18^ and subsequently extended to several treatment and subpopulation selection rules.^4,6,19–22,35,36^

Conditional on the selection made, for each j∈S, the estimator ${\hat{\theta }}_{2,j}$ provides an unbiased estimator for *θ*_*j*_. By the Rao-Blackwell theorem, the UMVCUE is the expected value of this estimator given the sufficient and complete statistic. Here, the UMVCUE is conditional on the subpopulation selection rule used, the partitions selected to continue to stage 2 and the observed data. This is reflected in the UMVCUE for *θ_j_* by its expression having terms for the lower and upper bounds for ${\hat{\theta }}_{1,j}$ that are determined based on the selection rule, the selected partitions, and the observed stage 1 data. Since the lower and upper bounds depend on the stage 1 data, they are random variables which we denote by *L*_*j*_ and *W*_*j*_, with observed values *l*_*j*_ and *w*_*j*_, respectively. Let $p_{j}^{\prime }=\sum_{i=1}^{j}p_{i}(j=1,\ldots ,K)$. For the adaptive threshold enrichment design selection rule in Section 2.3, when a subpopulation consisting of *s* (*s* = 1,...,*K*) partitions is selected, for each $j\in \{1,...,s\}=S,w_{j}=(p_{s}^{\prime }b-\sum_{\begin{array}{c} i=1\\ i\neq j \end{array}}^{s}p_{i}{\hat{\theta }}_{1,i})/p_{j}$ (the term $\sum_{\begin{array}{c} i=1\\ i\neq j \end{array}}^{s}p_{i}{\hat{\theta }}_{1,i}$ is set to zero when *s =* 1) and

$$l_{j}=\max\{\frac{p_{s+1}^{\prime }b-\sum_{\begin{array}{c} i=1\\ i\neq j \end{array}}^{s+1}p_{i}{\hat{\theta }}_{1,i}}{p_{j}}\frac{p_{s+2}^{\prime }b-\sum_{\begin{array}{c} i=1\\ i\neq j \end{array}}^{s+2}p_{i}{\hat{\theta }}_{1,i}}{p_{j}},\ldots ,\frac{p_{K}^{\prime }b-\sum_{\begin{array}{c} i=1\\ i\neq j \end{array}}^{K}p_{i}{\hat{\theta }}_{1,i}}{p_{j}}\},$$

with *l*_*j*_ set to be −∞ if all partitions are selected. For the selection rule of continuing to stage 2 with any partition whose treatment effect is ≤ *b* (second rule in Section 2.3), for all $j\in S,l_{j}=-\infty$ and *w*_*j*_ = *b*. For *K* = 2, the points corresponding to the expressions for *l*_*j*_ and *w*_*j*_ are illustrated in Figure 3. For estimating *θ*_*1*_, l_*1*_ and *w*_1_ are the lower and upper edges of the vertical dashed and dotted lines that go through the stage 1 estimates, respectively. For estimating *θ*_*2*_, l_*2*_ and *w*_2_ are the right and left hand edges of the horizontal lines that go through the stage 1 estimates, respectively. The details of how the bounds for the adaptive threshold enrichment design selection rule and some other selection rules suggested in literature are derived are given in the supplementary material.

Let Qs denote the event of the observed data and S. Suppose that |S|=s and that the *s* selected partitions are indexed 1,...,*s*. Define ${\hat{\theta }}_{N_{1}^{*}}=\left(\sigma_{2,1}/\sigma_{1,1}\right){\hat{\theta }}_{1,1}+\left(\sigma_{1,1}/\sigma_{2,1}\right){\hat{\theta }}_{2,1}$, the vector ${\left({\hat{\theta }}_{N_{1}^{*}},{\hat{\theta }}_{1,2},\ldots ,{\hat{\theta }}_{1,K},{\hat{\theta }}_{2,2},\ldots ,{\hat{\theta }}_{2,s}\right)}^{\prime }$ is sufficient and complete for estimating *θ*_1_. Therefore, the UMVCUE for *θ*_1_ is the expression for $E\left[{\hat{\theta }}_{2,1}|{\hat{\theta }}_{N_{1}^{*}},{\hat{\theta }}_{1,2},\ldots ,{\hat{\theta }}_{1,K},{\hat{\theta }}_{2,2},\ldots ,{\hat{\theta }}_{2,s},Q_{S}\right]$. The expression is obtained by deriving the conditional density $f_{Q_{S}}\left({\hat{\theta }}_{2,1}|{\hat{\theta }}_{N_{1}^{*}},{\hat{\theta }}_{1,2},\ldots ,{\hat{\theta }}_{1,K},{\hat{\theta }}_{2,2},\ldots ,{\hat{\theta }}_{2,s}\right)$ with $E\left[{\hat{\theta }}_{2,1}|{\hat{\theta }}_{N_{1}^{*}},{\hat{\theta }}_{1,2},\ldots ,{\hat{\theta }}_{1,K},{\hat{\theta }}_{2,2},\ldots ,{\hat{\theta }}_{2,s},Q_{S}\right]$ obtained by deriving the expression for $\int {\hat{\theta }}_{2,1}f_{Q_{S}}\left({\hat{\theta }}_{2,1}|{\hat{\theta }}_{N_{1}^{*}},{\hat{\theta }}_{1,2},\ldots ,{\hat{\theta }}_{1,K},{\hat{\theta }}_{2,2},\ldots ,{\hat{\theta }}_{2,s}\right)d{\hat{\theta }}_{2,1}$. The UMVCUEs for the effects in the other selected partitions are obtained similarly. We show in the supplementary material that for each j∈S, the UMVCUE for *θ*_*j*_ is given by

(3) $${\hat{\theta }}_{U_{j}}={\hat{\theta }}_{N_{j}}-\frac{\sigma_{2,j}^{2}}{\sqrt{\sigma_{1,j}^{2}+\sigma_{2,j}^{2}}}\frac{\phi \left(g\left(L_{j}\right)\right)-\phi \left(g\left(W_{j}\right)\right)}{\Phi \left(g\left(L_{j}\right)\right)-\Phi \left(g\left(W_{j}\right)\right)},$$

where $g(x)=\frac{\sqrt{\sigma_{1,j}^{2}+\sigma_{2j}^{2}}}{\sigma_{1j}^{2}}\left({\hat{\theta }}_{N_{j}}-x\right)$, and *ϕ* and Φ denote the density and distribution functions of a standard normal, respectively.

For the special case of ${\tilde{t}}_{1}=t_{1},{\hat{\theta }}_{U_{j}}$ is an asymptotic UMVCUE for *θ*_*j*_. However, when patients without events at *t*_1_ are followed in stage 2, that is, ${\tilde{t}}_{1}>t_{1},{\hat{\theta }}_{U_{j}}$is an approximate asymptotic UMVCUE for *θ*_*j*_ meaning in some scenarios it may have small biases because as described in Section 2.2, the independent structure which is assumed in the derivation of ${\hat{\theta }}_{U_{j}}$ is an approximate assumption. Like any estimator based on the asymptotic score statistic distribution(s), ${\hat{\theta }}_{U_{j}}$may be biased because score statistic distributions, such as those summarized in the last paragraph of Section 2, are asymptotic distributions that assume the value of *θ*_*j*_ is close to zero, that is, a small effect size. These aspects will be explored further in a simulation study in Section 5.

### 3.2 ∣ A new method for constructing confidence intervals

In this section, we construct new simultaneous confidence intervals that are based on the duality between hypothesis testing and confidence intervals. To account for the stage 1 patients that are followed further in stage 2 because they did not have an event at the interim analysis, we propose hypothesis testing using the strategy suggested by Jenkins et al.^3^ They combine evidence from stages 1 and 2 using a *P*-value combination function and adjust for multiple hypotheses by the closure principle (CP).^37^ Let *H*_*j*_ (*j* = 1, … *,K*) denote the elementary null hypothesis $\theta_{j}=0$ and $H_{I}(I\subseteq \{1,\ldots ,K\})$ the intersection null hypothesis $\cap_{i\in I}H_{i}$, where for simplicity, for example, we write *H*_12_ for *H*_{1,2}_. We derive the expressions for the lower and upper bounds separately based on one-sided tests. For the lower bounds, the alternative hypothesis for $H_{I}(I\subseteq \{1,\ldots ,K\})$ is that for at least one *j* ∈ *I*, *θ*_*j*_ *>* 0 and we denote the corresponding one-sided *P*-value for *H*_*I*_ obtained using data from patients recruited in stage *k* (*k* = 1, 2) only by $p_{k,I}^{+}$. Note that *θ*_*j*_ *>* 0 indicates that the experimental treatment is inferior in partition *j* and that a lower bound below 0 is not sufficient to conclude that the experimental treatment is significantly beneficial. The *P*-value $p_{k,I}^{+}(k=1,2)$ is obtained using stage *k* patients only since the *P*-value combination functions assume that $p_{1,I}^{+}$ and $p_{2,I}^{+}$ are independent. Therefore, $p_{2,I}^{+}$is computed by separately analysing the patients whose survival times correspond to the wholly dotted lines in Figure 2. For the selected partitions, while computing $p_{1,I}^{+}$ using the stage 1 patients, so as to include the incremental data in hypothesis testing, following Jenkins et al, the survival time and status are determined at time ${\tilde{t}}_{1}$. Consequently, the survival times for patients with events at the interim analysis correspond to the continuous lines in Figure 2, while the survival times for patients without events at the interim analysis correspond to the lines consisting of continuous and dashed segments. While computing $p_{1,I}^{+}$, if the patients in the dropped partitions are followed after the interim analysis, as for the selected partitions, their survival times and status are determined at ${\tilde{t}}_{1}$. However, if the patients in the dropped partitions are not followed after the interim analysis, their survival times and status are determined at *t*_1_ in the computation of $p_{1,I}^{+}$ so that their survival times correspond to the continuous line segments in Figure 2. We described in Section 2.2 how to decide whether to follow up to ${\tilde{t}}_{1}$ stage 1 patients from dropped partitions. For stage *k* (*k* = 1, 2) patients, since there is no overlap in the data used to obtain $p_{k,I}^{+}$ and the data used to compute $p_{k,i^{\prime }}^{+}\left(i\neq i^{\prime }\right)$, we recommend the Šidak adjusted *P*-value given by $p_{k,I}^{+}=1-{\left(1-\min_{i\in I}\left\{p_{k,i}^{+}\right\}\right)}^{\left|I\right|}$ because its type I error rate is exact. Using the weighted inverse normal method^38^ to combine the evidence from the two stages, the combined *P*-value $C\left(p_{1,I}^{+},p_{2,I}^{+}\right)=1-\Phi \left\{\omega_{1}\Phi^{-1}\left(1-p_{1,I}^{+}\right)+\omega_{2}\Phi^{-1}\left(1-p_{2,I}^{+}\right)\right\}$, where *ω*_1_ and *ω*_2_ are prespecified weights such that $\omega_{1}^{2}+\omega_{2}^{2}=1$. We take *ω*_*k*_ (*k* = 1, 2) to be the square root of the proportion of the prespecified total number of events from stage *k* patients. To control the type I error rate by the CP, it is concluded that *θ*_*j*_ *>* 0 (*j* = 1, … *, K*) if all hypotheses $H_{I}(I\subseteq \{1,\ldots ,K\})$, with *j* ∈ *I* are rejected or equivalently if the adjusted *P*-value $\max_{I}\left\{C\left(p_{1,I}^{+},p_{2,I}^{+}\right)\right\}\leq \alpha /2$, with *j* ∈ *I*. To allow for the dropped partitions, the stage 2 *P*-value $p_{2,I}^{+}$ is obtained using the test for $H_{I\cap S}$, with $p_{2,I}^{+}$ set to be 1 if I∩S=∅.

Stage 1 pairwise *P*-values $p_{1,j}^{+}(j=1,\ldots ,K)$ that are required in the expression for the Šidak adjusted *P*-value, $p_{1,I}^{+}$, can be obtained using statistics similar to those in Section 2.2. Let ${\tilde{S}}_{1,j}$ and ${\tilde{V}}_{1,j}(j=1,\ldots ,K)$ denote the score statistic and Fisher information for partition *j* stage 1 patients with survival time and status evaluated at ${\tilde{t}}_{1}$ if partition *j* patients without events at *t*_1_ are followed in stage 2 until ${\tilde{t}}_{1}$ and at *t*_1_ if partition *j* patients without events at *t*_1_ are not followed in stage 2. Defining ${\tilde{\theta }}_{1,j}={\tilde{S}}_{1,j}/{\tilde{V}}_{1,j}$ and ${\tilde{\sigma }}_{1,j}^{2}=1/{\tilde{V}}_{1,j},p_{1,j}^{+}=1-\Phi \left({\tilde{\theta }}_{1,j}/{\tilde{\sigma }}_{1,j}\right)$. Similarly, for each j∈S, stage 2 pairwise *P*-value $p_{2,j}^{+}$ that is required in the expression for the Šidak adjusted *P*-value, $p_{2,I}^{+}$, is computed using the score statistic and Fisher information obtained by separately analyzing the patients corresponding to the dotted lines in Figure 2.

Magirr et al^23^ developed simultaneous confidence intervals following two-stage adaptive clinical trials with treatment selection that are based on the duality between confidence intervals and hypothesis testing. They assume a hypothesis testing approach that is similar to that we have proposed above. Following their work, we give simultaneous confidence intervals following two-stage adaptive clinical trials with subpopulation selection that are compatible with the above testing procedure. Magirr et al describe the theory of how to obtain a confidence region with the correct coverage and subsequently how to extract simultaneous confidence intervals. We do not repeat the theory and only focus on giving the expressions for the confidence intervals in our setting. We give the expressions assuming that the Šidak adjustment is used for the intersection hypotheses. The expressions are functions of the *P*-values for the generalized null hypotheses. Let $H_{j}\left(\theta_{j}^{*}\right)$ denote the generalized null hypothesis $\theta_{j}=\theta_{j}^{*}$ and for I⊆{1,…,K}, we write *H*_*I*_(*θ*^∗^) for the generalized intersection hypothesis $\cap_{i\in I}H_{i}\left(\theta_{i}^{*}\right)$. For an observed stage *k* (*k* = 1, 2) dataset **x**_*k*_, the generalized *P*-value for *H*_*I*_ is $p_{k,I}^{+}\left(\theta^{*},x_{k}\right)$ and is computed as $\mathrm{Prob}\left(X_{k}\geq x_{k};\theta^{*}\right)$, where $\theta^{*}={\left(\theta_{1}^{*},\ldots ,\theta_{K}^{*}\right)}^{\prime }$. The combined *P*-value for *H*_*I*_(*θ*^∗^) is $C\left(p_{1,I}^{+}\left(\theta^{*},x_{1}\right),p_{2,I}^{+}\left(\theta^{*},x_{2}\right)\right)=1-\Phi \left\{\omega_{1}\Phi^{-1}\left(1-p_{1,I}^{+}\left(\theta^{*},x_{1}\right)\right)+\omega_{2}\Phi^{-1}\left(1-p_{2,I}^{+}\left(\theta^{*},x_{2}\right)\right)\right\}$. The Šidak adjusted generalized *P*-value, $p_{k,I}^{+}\left(\theta^{*},x_{k}\right)(k=1,2)$, is given by $1-{\left(1-\min_{i\in I}\left\{p_{k,i}^{+}\left(\theta_{i}^{*},x_{k}\right)\right\}\right)}^{\left|I\right|}$, where $p_{k,i}^{+}\left(\theta_{i}^{*},x_{k}\right)$ is the generalized pairwise *P*-value. As an example, $p_{1,j}^{+}\left(\theta_{j}^{*},x_{1}\right)(j=1,\ldots ,K)$is given by 1 $1-\Phi (\left(\left({\tilde{\theta }}_{1,j}-\theta_{j}^{*}\right)/{\tilde{\sigma }}_{1,j}\right)$.

Let $p_{M}^{+}$ be the maximum stage 1 *P*-value for all the intersection hypotheses $H_{I}(I\subseteq \{1,\ldots ,K\}\backslash S)$. For example if *K* = 4 and $S=\{1,2\},p_{M}^{+}=\max\left\{p_{1,3}^{+},p_{1,4}^{+},p_{1,34}^{+}\right\}$. If all partitions are selected to continue to stage 2 so that $\{1,\ldots ,K\}\backslash S=\emptyset ,p_{M}^{+}$ is set equal to 0. If at the end of stage 2 it is concluded that *θ*_*j*_ *>* 0 for all j∈S, then for each j∈S, the lower bound for the effect in partition *j* is given by

(4) $$\theta_{j,L}=\max\left[0,\sup\left\{v:C\left(\max\left\{p_{M}^{+},1-{\left(1-p_{1,j}^{+}\left(v,x_{1}\right)\right)}^{K}\right\},1-{\left(1-p_{2,j}^{+}\left(v,x_{2}\right)\right)}^{\left|S\right|}\right)\leq \alpha /2\right\}\right],$$

Note that $p_{k,j}^{+}\left(v,x_{k}\right)(k=1,2)$ is a generalized pairwise *P*-value for *H*_*j*_ and so it is computationally quick to find the root.

If at the end of the trial, for some j∈S, it is not concluded that *θ*_*j*_ *>* 0, the expression for the lower bound of the effect in a partition depends on the outcome of the hypothesis testing. For j∈S where it is concluded that *θ*_*j*_ *>* 0, the lower Bound for *θ*_*j*_ is

(5) $$\theta_{j,L}=0.$$

For $H_{I}(I\subseteq \{1,\ldots ,K\})$, based on stage *k* (*k* = 1, 2) patients’ data, we define $p_{k,I}^{+}(j,v)=\mathrm{Prob}\left(X_{k}\geq x_{k};\theta^{j,v}\right)$, where *θ*^*j*,*v*^ is a *K* × 1 vector whose *j*th entry is *ν* and the other entries are zero. For I⊆{1,…,K} with *j* ∈ *I*, we define $\theta_{j,L}^{I}=\infty \text{ if }C\left(p_{1,I}^{+},p_{2,I}^{+}\right)<\alpha /2$ and $\theta_{j,L}^{I}=\sup\left\{v:C\left(p_{1,I}^{+}(j,v),p_{2,I}^{+}(j,v)\right)\leq \alpha /2\right\}$ otherwise. For j∈S where it is not concluded that *θ*_*j*_ *>* 0, the lower bound for *θ*_*j*_ is

(6) $$\theta_{j,L}=\underset{I\subseteq \{1,\ldots ,K\}}{\min}\left\{\theta_{j,L}^{I}\right\}.$$

Note that in this case where at the end of the trial, for some j∈S, it is not concluded that *θ*_*j*_ *>* 0, the confidence intervals for the effects in the partitions where it is concluded that the log HRs are greater than 0 are not informative. This is because from expression (5), the lower bounds for those partitions are fixed to be 0 regardless of the values of the point estimates and the adjusted *P*-values. This is a drawback of the method.^23^ We emphasize that the lower bounds obtained using expression (6) are informative and those obtained using expression (4) would be expected to be informative most of the time. So non-informative lower bounds are mostly obtained when more than one partition is selected to continue to stage 2 and it is concluded that the log HR in at least one partition is not greater than 0 (the lower bounds in such partitions are informative) and it is concluded that the log HR in at least one partition is greater than 0 (the lower bounds for such partitions are set to be 0 and hence non-informative).

To derive the upper bounds, the alternative hypothesis for $H_{I}(I\subseteq \{1,\ldots ,K\})$ is that for at least one *j* ∈ *I*, *θ*_*j*_ *<* 0. Note that, when the upper bound is less than 0, then the experimental treatment is significantly superior. Let $\delta_{j}^{*}=-\theta_{j}^{*}$ and ${\tilde{\delta }}_{k,j}=-{\tilde{\theta }}_{k,j}$, the stage 1 “conventional” and generalized *P*-values in this case are $p_{1,j}^{-}=\Phi \left({\tilde{\theta }}_{1,j}/{\tilde{\sigma }}_{1,j}\right)=1-\Phi \left({\tilde{\delta }}_{1,j}/{\tilde{\sigma }}_{1,j}\right)$, and $p_{1,j}^{-}\left(\theta_{j}^{*},x_{1}\right)=\Phi \left(\left({\tilde{\theta }}_{1,j}-\theta_{j}^{*}\right)/{\tilde{\sigma }}_{1,j}\right)=1-\Phi \left(\left({\tilde{\delta }}_{1,j}-\delta_{j}^{*}\right)/{\tilde{\sigma }}_{1,j}\right)$ respectively. Note that $p_{1,j}^{-}$ is the *P*-value for the hypothesis test *θ*_*j*_ *=* 0 against *θ*_*j*_ *<* 0 as well as for the hypothesis test *δ*_*j*_ *=* 0 against *δ*_*j*_ *<* 0 as well as for the hypothesis test *δ*_*j*_ = 0 against *δ*_*j*_ *>* 0, where *δ*_*j*_ = −*θ*_*j*_. Therefore, as we do in Sections 4 and 5, the upper bound for the effect in partition *j*, *θ*_*j*,*U*_, can be obtained as follows. Change the signs of the point estimates, for example, changing ${\tilde{\theta }}_{1,j}$ to $-{\tilde{\theta }}_{1,j}$, and then obtain the lower bound, *δ*_*j*,*L*_, for *δ*_*j*_ = −*θ*_*j*_ as described for *θ*_*j*_ above. The upper bound for the effect in partition *j* is *θ*_*j*,*U*_ = −*δ*_*j*,*L*_. As with the lower bounds, the upper bounds can be non-informative.

## 4 ∣ EXAMPLE

To illustrate how to compute the various estimates, we construct a two-stage enrichment trial using data from a single-stage trial that compared intravenous methotrexate (C-MTX) and high-dose methotrexate (HDMTX) in the treatment of T-cell acute lymphoblastic leukemia (T-ALL) in children.^10^ The numbers of patients allocated to C-MTX and HDMTX were 519 and 512, respectively. Based on the clinical features, the patients were categorized as low risk (LRi), intermediate risk (IRi), and high risk (HRi). The analysis included assessing separate effects in 109 LRi, 707 IRi, and 215 HRi patients and so we take the risk level as the biomarker. So as not to have too few events in each stage of the constructed example, we use disease free survival (DFS). The conclusion from the trial was that C-MTX is superior to HDMTX. The observed advantage of C-MTX over HDMTX increased with risk level and was statistically significant for intermediate and high risk levels. Although the aim of the trial was to assess which of C-MTX and HDMTX is superior, for the constructed example, we take HDMTX and C-MTX to be the control and experimental treatment, respectively. Also, because of the few LRi patients and events from them, for the constructed example, we combine LRi and IRi patients into one category. Therefore, we have two partitions, one consisting of the HRi patients and the other consisting of the LRi and IRi (LRi/IRi) patients.

We take the futility boundary *b* = 0. Based on the observed monotonic relationship between treatment effect and risk level on the initial categorization (LRi, IRi, and HRi), the adaptive threshold enrichment design could be used. For this design, based on Figure 1, partitions 1 and 2 correspond to HRi and LRi/IRi patients, respectively. If for this design we use the first selection rule in Section 2.3, we need to specify the prevalences of the partitions, which we have assumed to be known. In this illustrative example, we use the observed prevalences in the entire trial (*p*_1_ = 0.2 and *p*_2_ = 0.8). Since we do not pool estimates from multiple partitions, the estimates remain valid for any set of prevalences. However, if better guesses of the prevalences are available, for example, from historical data, they could be used as they influence the probability of selecting the desired partitions. The selection rule of continuing with any partition whose stage 1 estimate is ≤ *b* can also be used with this example. We will demonstrate how to compute estimates based on both selection rules.

Patients were recruited in 2720 days, the last follow-up was 3644 days after recruitment started, and there were 122 events. We assume that the interim analysis was conducted after 60 events (21 in partition 1 and 39 in partition 2), which corresponds to 2176 days after recruitment started and when 80% of the patients (stage 1 patients) had been recruited. The number of events from the 20% of the patients who were recruited after the interim analysis (stage 2 patients) is 31 (13 in partition 1 and 18 in partition 2). The number of events from stage 1 patients who did not have events at the interim analysis was 31 (11 in partition 1 and 20 in partition 2). The last follow-up of stage 2 patients was 3644 days from when the trial first recruited. We assume that it was prespecified that the follow-up of stage 2 patients will stop after 31 events were observed from them and that this happened 3644 days from when recruitment started. This assumption enables us to describe how ${\tilde{t}}_{1}$ that is different from *t*_2_ can be prespecified, and the consequences of this. We assume that it was prespecified that stage 1 patients without events at the interim analysis will be followed for 3.5 years after the interim analysis, which corresponds to 3455 days from when recruitment started. This resulted in not including in the final analysis one DFS event from stage 1 patients who did not have events at *t*_1_. Note that *t*_1_, ${\tilde{t}}_{1}$, and *t*_2_ correspond to calendar dates 2176, 3455, and 3644 days from the date of first enrolment, respectively.

Details of formatting the data and the R^39^ code used to analyze them are provided in the supplementary material. The estimates are summarized in Table 1. The stage 1 estimates in partitions 1 and 2 are ${\hat{\theta }}_{1,1}=-0.902$ and ${\hat{\theta }}_{1,2}=-0.419$, respectively. For the adaptive threshold enrichment design selection rule, since $\left(p_{1}{\hat{\theta }}_{1,1}+p_{2}{\hat{\theta }}_{1,2}\right)=-0.516<b(=0)$, both partitions (*F*) are selected to continue to stage 2. The naive estimates for partitions 1 and 2 are ${\hat{\theta }}_{N_{1}}=-0.746$ and ${\hat{\theta }}_{N_{2}}=-0.362$, respectively. Since *F* is selected, from Section 3.1, $l_{1}=l_{2}=-\infty ,w_{1}=\left(p_{2}^{\prime }b-\sum_{i=1}^{2}p_{i}{\hat{\theta }}_{1,i}\right)/p_{1}=\left(b-p_{2}{\hat{\theta }}_{1,2}\right)/p_{1}=(0-[0.8\times -0.419])/0.2=1.676$.Similarly, $w_{2}=\left(b-p_{1}{\hat{\theta }}_{1,1}\right)/p_{2}=0.226$. For example, for partition 1, substituting ${\hat{\theta }}_{N_{j}},\sigma_{1,j}^{2},\sigma_{2,j}^{2},L_{j}$ and *W*_*j*_ in Equation (3) with ${\hat{\theta }}_{N_{1}}=-0.746,\sigma_{1,1}^{2}=0.191,\sigma_{2,1}^{2}=0.167,l_{1}=-\infty$, and *w*_1_ = 1.676, respectively, the UMVCUE for the effect in partition 1 is ${\hat{\theta }}_{U_{1}}=-0.737$. Similarly, the UMVCUE for the effect in partition 2 is ${\hat{\theta }}_{U_{2}}=-0.359$. The UMVCUEs are closer to zero than the corresponding naive estimates. With the selection rule of continuing with any partition whose stage 1 estimate ≤ *b*(= 0), *F* is selected since ${\hat{\theta }}_{1,1}=-0.902<0$ and ${\hat{\theta }}_{1,2}=-0.419<0$. From Section 3.1, *l*_1_ = *l*_2_ = −∞ and *w*_1_ = *w*_*2*_ = *b* = 0. All the other quantities to substitute in Equation (3) are the same as for the adaptive threshold rule. The UMVCUEs for the rule of continuing with any partition whose the observed stage 1 effect is ≤ 0 (independently selecting partitions) are closer to zero (${\hat{\theta }}_{U_{1}}=-0.631$ and ${\hat{\theta }}_{U_{2}}=-0.335$) than the corresponding naive estimates and the UMVCUEs for the adaptive threshold design. This indicates that the naive estimates may have bigger biases when a partition is selected independent of the observed effects in the other partitions compared to when partitions are selected using the adaptive threshold design selection rule.

The naive and duality confidence intervals are conditional on the number of partitions selected and not the selection rule used to select the partitions. Hence, since *F* was selected with the above two selection rules, the naive and duality confidence intervals are the same for the two selection rules. For the naive confidence interval, for $\alpha =0.05,z_{\alpha /(2|S|)}$ in expression (2) is $z_{\alpha /(2\times 2)}=2.241$. The values for ${\hat{\theta }}_{N_{j}}$. and $\sigma_{N_{j}}(j=1,2)$ are given in Table 1. Consequently, the naive confidence intervals for the effects in partitions 1 and 2 are (−1.415, −0.077) and (−0.876, 0.153), respectively. For the duality confidence intervals, we take the weights $\omega_{1}=\sqrt{0.75}$ and $\omega_{2}=\sqrt{0.25}$. These are approximately proportional to the number of events from stages 1 and 2 patients, which is optimal in combining stages 1 and 2 evidence.^38^ This assumes that, in advance, we could tell how many events will be observed from patients recruited in each stage. In practice, it may not be possible to specify optimal weights. Since there are two partitions, the null hypotheses tested are H_*1*_ (*θ*_1_ = 0), *H*_*2*_(*θ*_2_ = 0), and *H*_{1,2}_(*θ*_1_ = *θ*_2_ = 0). For the lower bound, the alternative hypothesis for *H*_*j*_ (*j* = 1, 2) is *θ*_*j*_ *>* 0, while the alternative hypothesis for *H*_{1,2}_ is *θ*_1_ *>* 0 or *θ*_2_ *>* 0. The stagewise and the combined *P*-values are given in the supplementary material (Figure S2). The adjusted *P*-values for partitions 1 and 2 are both equal to 0.998 so that we do not conclude the effects are greater than 0. Therefore, to get the lower bounds of the effects in both partitions, we use expression (6) giving the lower bounds for the effects in partitions 1 and 2 as −1.499 and −0.911, respectively. For the upper bound, the alternative hypothesis for *H*_*j*_ (*j* = 1, 2) is *θ*_*j*_ *<* 0, while the alternative hypothesis for *H*_{1,2}_ is *θ*_1_ *<* 0 or *θ*_2_ *<* 0. The stagewise and the combined *P*-values are given in the supplementary material (Figure S3). The adjusted *P*-values for partitions 1 and 2 are 0.0179 and 0.0601, respectively, so that the conclusion is that the log HR in partition 1 is less than 0, while we do not conclude that the log HR in partition 2 is less than 0. Hence, the confidence interval for the effect in partition 1 is not informative with upper bound fixed to be 0 by expression (5). When the duality confidence interval upper bound is not informative, in Section 6, we propose using the naive upper bound to make a decision on the treatment effect. For the upper bound for the effect in partition 2, we use expression (6) and the last paragraph in Section 3.2 to obtain 0.093.

To assess the impact of a bigger trial and to demonstrate how to use expression (4) to compute duality confidence intervals’ limits, we combined the above data with a bootstrap sample with the same number of patients. The proportion of events at the interim analysis is the same. The results are in the supplementary material (Table S2 and Section 5.3). The bias corrections for some of the naive estimates are smaller. This may be attributed to more precise stage 1 estimates, which may indicate the treatment effects are less than 0 and hence less correction for the futility rule. Also, the log HRs in both partitions are concluded to be less than 0 and so the duality confidence intervals for the effects in the two partitions are informative.

## 5 ∣ SIMULATION STUDY

### 5.1 ∣ The simulation study setting

For data generation, we assumed the Weibull distribution with the hazard function for death for treatment *i* (*i* = *C, E*) in partition *j* (*j* = 1, … *,K*) parameterized as

$$h_{i_{j}}(t)=\lambda_{i_{j}}\gamma_{i_{j}}t^{\gamma_{ij}-1},$$

where *t* is time (in days for the simulation study), and $\lambda_{i_{j}}$ and $\gamma_{i}$ are the scale and shape parameters, respectively. In all simulation scenarios, we considered the case of $\gamma_{i_{j}}=\gamma$. For two scenarios where the HRs in partitions are the same, but in one scenario the biomarker is prognostic while in the other the biomarker is not prognostic, the properties of the new estimators are expected to be the same. Therefore, in all simulation scenarios, we only considered the case of a biomarker that is predictive but not prognostic taking the scale parameter for the control group in all partitions to be $\lambda_{C_{j}}=\lambda_{C}$. In most simulations, we will take *γ*= 0.5 but in order to assess the effect of the shape parameter, we will compare some results for *γ* = 0.5 with the cases of *γ* = 1 (exponential distribution) and *γ* = 1.5.

The log HR in partition *j* (*j* = 1, … *, K*) is given by $\theta_{j}=\ln\left(\lambda_{E_{j}}/\lambda_{C}\right)$. In most simulations, we considered four partitions of equal prevalences (quartiles) and three configurations for (*θ*_1_*, θ*_2_*, θ*_3_*, θ*_4_)′, which are (0.0198, 0.0198, 0.0198, 0.0198)^′^, (−0.2231, −0.0953, 0.3364, 0.4055)^′^, and (−0.4055, −0.2231, −0.0953, 0)^′^. Log HRs equal to −0.4055, −0.2231, −0.0953, 0, 0.0198, 0.3364, and 0.4055 correspond to HRs equal to 0.6667, 0.80, 0.9091, 1, 1.02, 1.4, and 1.5, respectively. In all simulations, we set the futility boundary *b* = 0.

Sample sizes in the simulations are selected such that a power of approximately 80% would be obtained in a single-stage one-year trial with a HR of 0.8. This corresponds to a setting typical of many oncology trials. The hazard function parameters and the required number of deaths and patients are given in Table 2. For the control arm, we set the scale parameters so that the median survival time is 400 days, with the scale parameters for the experimental arm chosen so that *λ*_*E*_∕*λ*_*C*_ = 0.8. For 80% power, the required number of deaths is 630 in all scenarios, while the required numbers of patients are 2060, 2600, and 3300 for *γ*= 0.5, *γ* = 1.0, and *γ* = 1.5, respectively.^40^ Informed by these sample sizes for one-year single-stage trials, we considered two-stage trials with an interim analysis after 300 deaths and with stage 2 patients followed until 300 deaths are observed from them. For *γ* = 0.5, *γ* = 1, and *γ* = 1.5, we assumed, respectively, 2200, 2800, and 3400 patients can be recruited uniformly over two years.

We will assess the properties of the various estimators for the case where stage 1 patients without events at *t*_1_ are not followed in stage 2, that is, ${\tilde{t}}_{1}=t_{1}$, and for the case where they are followed in stage 2 up to time ${\tilde{t}}_{1}>t_{1}$, which corresponds a prespecified number of days after *t*_1_. Since it is expected that stage 2 is about 1 year, we set ${\tilde{t}}_{1}$ to correspond to 250 days after *t*_1_ for *γ* = 0.5. We choose fewer than 365 days so that it is unlikely that ${\tilde{t}}_{1}>t_{2}$ In a real trial, for *γ* = 1.0 and *γ*= 1.5, we would also choose ${\tilde{t}}_{1}$ to correspond to 250 days after *t*_1_. However, so that we have approximately the same total number of events in the simulated trials for *γ* = 0.5, *γ* = 1.0, and *γ* = 1.5, we set ${\tilde{t}}_{1}$ to correspond to 110 days and 77 days after *t*_1_ for *γ* = 1.0 and *γ* = 1.5, respectively. With these specifications for ${\tilde{t}}_{1}$, in the selected partitions, the average number of events at ${\tilde{t}}_{1}$ for stage 1 patients without events at *t*_1_ is approximately 40.

The scenarios we have described in this section cover a subset of the simulations undertaken. Other scenarios are considered in the last paragraph of Section 5.2 and in Section 5.3. For each scenario, we simulated 100 000 trials. For each simulated trial, for each selected partition, we computed two naive estimates corresponding to ${\tilde{t}}_{1}=t_{1}$ and ${\tilde{t}}_{1}>t_{1}$ and similarly two UMVCUE estimates. We also computed the naive confidence interval and the duality confidence interval.

### 5.2 ∣ Simulation results for the adaptive threshold enrichment design

We first consider the case of selecting partitions to continue to stage 2 using the adaptive threshold enrichment design selection rule described in Section 2.3. Table 3 shows the simulated probabilities of selecting different partitions under different settings. The probabilities for *γ* = 0.5, *γ* = 1.0, and *γ* = 1.5 are similar. For the first two configurations, the probabilities of making the ideal decisions (shown in bold in Table 3) are relatively small (32% and 34%, respectively). The naive estimators have more bias when the ideal decision is not made^6^ and so the naive estimators would be expected to have large biases when the probability of making the ideal decision is small.

**FIGURE 4:**
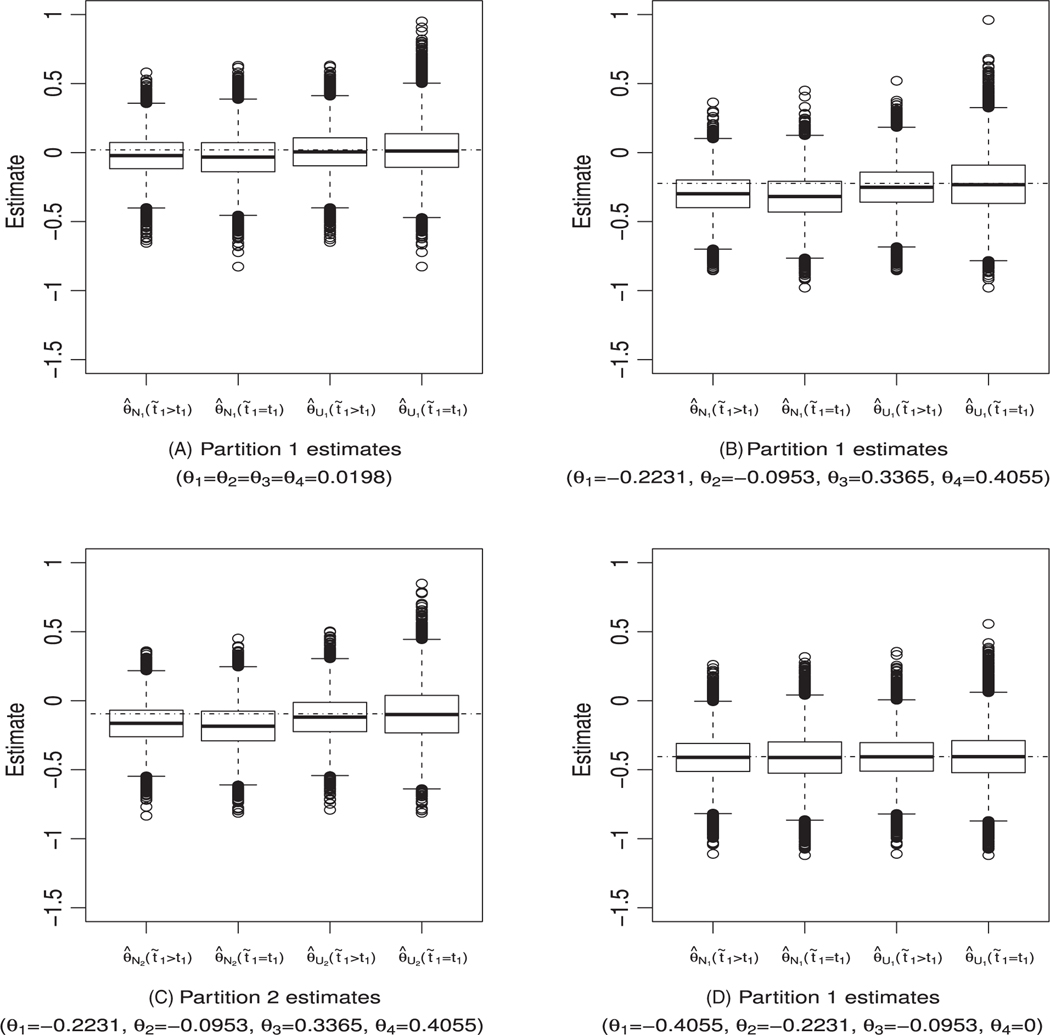
Boxplots for estimates in partition 1 (panels A, B, and D) and partition 2 (panel C) when the full population is selected to continue to stage 2 for Weibull distribution (*γ* = 0.5). The horizontal dashed and dotted line corresponds to the true log hazard ratio

Table 4 shows the simulated biases and root mean square errors (RMSEs) of the point estimators for *γ* = 0.5. Columns labeled ${\tilde{t}}_{1}=t_{1}$ correspond to when stage 1 patients without events at the interim analysis are not followed after *t*_1_, while columns labeled ${\tilde{t}}_{1}>t_{1}$ correspond to when stage 1 patients without events at the interim analysis are followed until ${\tilde{t}}_{1}$. A positive bias indicates that the estimator is overestimating the true effect size while a negative bias indicates that the estimator is underestimating the true effect size. We first describe the biases and RMSEs for the naive estimator $\left({\hat{\theta }}_{N_{j}}\right)$ For both ${\tilde{t}}_{1}>t_{1}$ and ${\tilde{t}}_{1}=t_{1},{\hat{\theta }}_{N_{j}}$ can be positively or negatively biased. The biases for ${\hat{\theta }}_{N_{j}}$ are smaller when ${\tilde{t}}_{1}>t_{1}$ than when ${\tilde{t}}_{1}>t_{1}$. Hence, the incremental data induce negative bias when subpopulation selection bias is positive and the incremental data induce positive bias when subpopulation selection bias is negative. In all cases, the RMSEs for ${\hat{\theta }}_{N_{j}}$ when ${\tilde{t}}_{1}>t_{1}$ are smaller than when ${\tilde{t}}_{1}=t_{1}$. Thus, the naive estimator has better properties when patients without events of interest at the interim analysis are followed further in stage 2. Next, for ${\tilde{t}}_{1}>t_{1}$, we compare the naive estimator $\left({\hat{\theta }}_{N_{j}}\right)$ to the UMVCUE $\left({\hat{\theta }}_{U_{j}}\right)$. Estimator ${\hat{\theta }}_{U_{j}}$ evaluated when ${\tilde{t}}_{1}>t_{1}$ is slightly biased in some cases but its biases are smaller than those for ${\hat{\theta }}_{N_{j}}$ evaluated when ${\tilde{t}}_{1}>t_{1}$, and the differences are big in some cases. Still focusing on when ${\tilde{t}}_{1}>t_{1}$, the RMSEs for ${\hat{\theta }}_{U_{j}}$ and ${\hat{\theta }}_{N_{j}}$ are close, sometimes with negligible difference so that when ${\tilde{t}}_{1}>t_{1}$, we consider ${\hat{\theta }}_{U_{j}}$ to be a better estimator than ${\hat{\theta }}_{N_{j}}$ since the two estimators have close RMSEs but the former has smaller biases. The summary so far is that we consider the UMVCUE *(i)*_*Uj*_*)* when i_1_ > t_1_ to be better than the naive estimator *(0*_*Nj*_*)* both when i_1_ > t_1_ and i_1_ = t_1_. Finally, we compare ${\hat{\theta }}_{U_{j}}$ when ${\tilde{t}}_{1}>t_{1}$ and when ${\tilde{t}}_{1}=t_{1}$. For ${\tilde{t}}_{1}=t_{1},{\hat{\theta }}_{U_{j}}$ is mean unbiased. This is expected since by derivation, when ${\tilde{t}}_{1}=t_{1},{\hat{\theta }}_{U_{j}}$ is an asymptotic UMVCUE. Although ${\hat{\theta }}_{U_{j}}$ when ${\tilde{t}}_{1}=t_{1}$ is mean unbiased, it has bigger RMSEs than ${\hat{\theta }}_{U_{j}}$ when ${\tilde{t}}_{1}>t_{1}$. We consider ${\hat{\theta }}_{U_{j}}$ when ${\tilde{t}}_{1}>t_{1}$ to be better than when ${\tilde{t}}_{1}=t_{1}$, since in the former, ${\hat{\theta }}_{U_{j}}$is only slightly biased but has smaller RMSEs. The results for the first row in Table 4 for Scenario 1 (*θ*_*1*_ *= θ*_*2*_ *= θ*_*3*_ *= θ*_*4*_ *=* 0.0198), the first two rows for Scenario 2 (*θ*_*1*_ *=* −0.2231, *θ*_*2*_ *=* −0.0953, *θ*_*3*_ *=* 0.3365, *θ*_*4*_ *=* 0.4055), and the first row for Scenario 3 (*θ*_*1*_ *=* −0.4055, *θ*_*2*_ *=* −0.2231, *θ*_*3*_ *=* −0.0953, *θ*_*4*_ *=* 0) are complemented by Figure 4A–D, respectively. We note that, even in the cases in Table 4 where ${\hat{\theta }}_{U_{j}}$ for ${\tilde{t}}_{1}>t_{1}$ seems to have noticeably more bias than when ${\tilde{t}}_{1}=t_{1}$, the median 50% estimates and the maximum values for ${\hat{\theta }}_{U_{j}}$ when ${\tilde{t}}_{1}>t_{1}$ are closer to the true value. Hence, the conclusion from Figure 4A–D is the same as that made from Table 4. Thus the summary from Table 4 and Figure 4 is that, for an adaptive trial with subpopulation selection, it is better to follow stage 1 patients without events of interest at the interim analysis up to a prespecified time ${\tilde{t}}_{1}>t_{1}$ in stage 2 and estimate the effects in partitions using the approximate asymptotic UMVCUE. Additionally, we note that for both ${\hat{\theta }}_{N_{j}}$ and ${\hat{\theta }}_{U_{j}}$, the estimators have smaller RMSEs when ${\tilde{t}}_{1}>t_{1}$ than when ${\tilde{t}}_{1}=t_{1}$. This feature would be expected in all scenarios since for ${\tilde{t}}_{1}>t_{1},{\hat{\theta }}_{N_{j}}$ and ${\hat{\theta }}_{U_{j}}$ contain additional information collected from stage 1 patients without events of interest at the interim analysis, which asymptotically are approximately an independent increment.

Table 5 summarizes the simultaneous properties for the confidence intervals of the effects in the selected partitions for *α* = 0.05. In most scenarios, the naive confidence regions have at least the desired 95% coverage probability. However, there are also several scenarios where they do not. Moreover, the “type I error” rate (non-coverage at upper end, which is defined as the probability that at least one upper bound is less than the true value) seems to be more severe than the violations of general coverage. Consequently, in general, the naive confidence intervals do not have the desired properties. For the duality confidence regions, in all scenarios, as desired, the coverage probabilities for the confidence regions are at least 95%. The confidence regions are not symmetric but the probabilities that at least one upper bound is less than the true value are below the desired 2.5%. However, these probabilities tend to be very small compared to the target 2.5%. This is partly due to the non-informative upper bounds. Hence, although the simultaneous duality confidence intervals have the desired coverage probabilities and type I error rates, they may be non-informative.

Results for other scenarios (*γ* = 1, *γ* = 1.5, slower recruitment rate, more events from stage 1 patients without events at *t*_1_, fewer events in a trial and conducting subpopulation earlier in the trial) are given in the supplementary material (Section 7, Tables S4 to S17, and Figures S5 to S11). In all scenarios, we recommend having ${\tilde{t}}_{1}>t_{1}$ and obtaining point estimates using ${\hat{\theta }}_{U_{j}}$. Furthermore, we consider the duality confidence regions to have at least the nominal coverage probabilities and the probabilities that at least one upper bound is less than the true value to be less than the target 2.5% but usually very small, which is partly explained by non-informative confidence intervals.

### 5.3 ∣ Simulation results for a different selection rule

To assess the characteristics of the various estimators when a different selection rule is used, we performed simulations for the case of continuing with any partition whose stage 1 log hazard ratio estimate is ≤ 0. This corresponds to the second selection rule in Section 2.3, with *b* = 0. The other aspects of the simulations are the same as those used to obtain the results in Table 4. The results for the point estimators for the three configurations of (*θ*_1_, *θ*_2_, *θ*_3_, *θ*_4_)′, which are (0.0198, 0.0198, 0.0198, 0.0198)′, (−0.2231, −0.0953, 0.3364, 0.4055)′ and (−0.4055, −0.2231, −0.0953, 0)′ are given in the supplementary material in Tables S18 to S20, respectively. The biases of the naive point estimator ${\hat{\theta }}_{N_{j}}$ are positive in all cases. This is because a partition is selected if it has a positive effect. In several scenarios, biases are larger than in the case of the adaptive threshold enrichment design (Results in Section 5.2). When ${\tilde{t}}_{1}>t_{1}$, the UMVCUE ${\hat{\theta }}_{U_{j}}$ is slightly biased in some cases but has smaller RMSE than when ${\tilde{t}}_{1}=t_{1}$. Hence we recommend having ${\tilde{t}}_{1}>t_{1}$ and using ${\hat{\theta }}_{U_{j}}$ to obtain estimates.

We expect the magnitudes of the biases for the point estimators for most selection rules that have a futility element to fall between the biases for the selection rule used in this section and the adaptive threshold selection rule considered in Section 5.2. This is because the selection rule in this section selects a partition based on the stage 1 observed effect in that partition only, while the adaptive threshold design considers stage 1 observed effects in all partitions and also assumes a relationship between the treatment effect and the biomarker value. Consequently, for most selection rules, we expect having ${\tilde{t}}_{1}>t_{1}$ and using estimator ${\hat{\theta }}_{U_{j}}$ as the best way of obtaining point estimates.

The simultaneous properties of the naive and the duality confidence intervals are summarized in the supplementary material (Table S21). For the two scenarios where the values for (*θ*_1_, *θ*_2_, *θ*_3_, *θ*_4_)′ are (0.0198, 0.0198, 0.0198, 0.0198)′ and (−0.4055, −0.2231, −0.0953, 0)′, unlike the naive confidence regions, the duality confidence regions have at least 95% coverage and the probabilities that at least one upper bound is less than the true value are less than 2.5%. For the other scenario of (*θ*_1_, *θ*_2_, *θ*_3_, *θ*_4_)′ equal to (−0.2231, −0.0953, 0.3364, 0.4055)′, the simulated probabilities (not reported in the table) for S equal to ∅, {1, 2}, {1} and {2} are 5.9%, 48.1%, 25.5%, and 10.9%, respectively. In these cases that constitute more than 90%, the duality confidence regions have at least 95% coverage probability and the probabilities that at least one upper bound is less than the true value are less than 2.5%. For the remaining cases, the naive confidence intervals have undesirable properties because the coverage probabilities are as small as 88% and the probabilities that at least one upper bound is less than the true value are as high as 12%. The coverage probabilities for the simultaneous duality confidence intervals are generally at least the target 95% but the probabilities that at least one upper bound is less than the true value are mostly above 2.5%, although much smaller than those of the naive confidence intervals. We note that this is driven by the upper bounds for the treatment effects in partitions 3 and 4. This may be considered to be of less practical impact since the log hazard ratios in these partitions are above 0 and the upper bounds are also mostly above 0 so that the new treatment would not be recommended in partitions 3 and 4. The reason that the duality upper bounds for the effects in partitions 3 and 4 do not show the desired properties is because the hypothesis testing described in Section 3.2 does not control the type I error rate conditional on the selection made but controls the probability of selecting any partition where the treatment is not effective and concluding it is effective. When the treatment is effective in some partitions and not in others, conditional on the selection, the type I error rate is above the target 2.5%. Hence, since we assessed the properties of the simultaneous confidence intervals conditional on the selection made, tail probabilities for such scenarios can be above 2.5%.

Based on the selection rule used in this section, we also performed simulations to assess the properties of the estimators for the case of bigger treatment effects. The simulated probabilities for S={1,2,3,4} and S={1,2,3} are 49.8% and 50.1%, respectively. The point estimation results are presented in the supplementary material (Table S22 and Figure S12). Even in scenarios where the selection bias is negligible, the point estimators are slightly negatively biased when the true hazard ratio is *<* 0.4. We describe the consequence of this finding in Section 6. We attribute the bias to the fact that the asymptotic distributions in Section 2.2 are based on the approximation of Taylor’s expansion of the likelihood function, and the accuracy improves as the effect size gets closer to zero.^27^ We have also assessed the properties of the confidence intervals for a scenario with big treatment effects and where the probability of having noninformative bounds with the duality confidence intervals is small (results in supplementary material Table S23). For both the naive confidence intervals and the duality confidence intervals, the probabilities that at least one upper bound is below the true value are smaller than 2.5%. As with point estimates, we attribute this to the underestimation of the treatment effects so that consequently the upper bounds are underestimated.

## 6 ∣ DISCUSSION

We have used the Rao-Blackwell theorem to derive a point estimator that adjusts for any subpopulation selection rule that is based on stage 1 estimates only in two-stage adaptive trials with time to event data. It is an asymptotic UMVCUE if the patients without events at stage 1 are not followed further in stage 2, while it is an approximate asymptotic UMVCUE if they are followed further in stage 2. When the stage 2 follow up length for stage 1 patients without events is specified before the trial, based on our simulation, the approximate asymptotic UMVCUE performed best. Unlike the case of normally distributed outcomes,^4,6^ compared with the naive estimator, this estimator did not have markedly higher RMSE and in some simulation scenarios, it outperformed the naive estimator in terms of RMSE. With time to event data, it is difficult to explore all factors that influence the properties of the various estimators. However, in our simulations, we considered several factors that may be encountered in real clinical trials and hence we expect the recommendation that the approximate UMVCUE is the best estimator to hold in most settings.

We have also described a new method for constructing simultaneous confidence intervals based on the duality between hypothesis testing and confidence intervals. In simulations, unlike the naive confidence intervals, the confidence regions corresponding to the new confidence intervals had at least the nominal coverage probability and also the probabilities that at least one lower bound was below the true value were acceptable. However, for example, as in the results in Table 1, the new confidence intervals can be non-informative. Focusing on the upper bounds, the non-informative confidence intervals are obtained in partitions where the treatment is concluded effective whenever in at least one selected partition, the treatment is not concluded effective. The probability of this happening depends on aspects such as the true treatment effects in partitions, the interim analysis sample size (events), the overall sample size (events), the selection rule, and the number of partitions. In the simulations, there was a high probability of this happening since most scenarios consisted of partitions where the new treatment is not effective and the futility rule only required stage 1 estimates to indicate the new treatment is as good as the control. More research to develop methods that do not give non-informative confidence intervals, such as extending existing work,^41^ is required.

We have assumed that there is no endpoint change between stages 1 and 2, such as using an early endpoint to make subpopulation selection in stage 1. This is appropriate in disease conditions such as pancreatic cancer where survival times are short and hence there is no practical advantage of considering an early endpoint in stage 1 and conditions such as uveal melanoma where an early endpoint does not exist.^42^ Increasingly, however, whenever practically feasible, adaptive clinical trials with time to event endpoints as the primary outcome(s) use time to some earlier event or different endpoints that are observed earlier than the primary outcome(s) to make adaptations in stage 1.^12^ The UMVCUE developed in this article can be extended by combining the techniques presented here and the techniques that consider using an early endpoint to make an adaptation.^43,44^ For the confidence intervals, the expressions for the new confidence intervals when there is change of endpoint are exactly the same as those in this article since they apply to any selection rule and hence no additional methodology is required. The coverage probabilities are, however, likely to be larger than those in this article since following Kimani et al,^45^ we expect the coverage probability to be closest to the nominal coverage if the same endpoint is used for subpopulation selection and estimation.

We have based the methodology on the score statistic. This has the advantage of producing estimates that align with the commonly used log rank test. However, since the asymptotic distribution of the score statistic holds best when the log hazard ratio is close to zero, we observed from the simulation study that for the cases where the true hazard ratio is < 0.4, the proposed approximate UMVCUE underestimates the treatment effect slightly. In real trials, this will have little impact since it is unlikely that the true hazard ratio is as small as 0.4. For example, for the stem cell therapies where relatively big treatment effects are observed, a simple online search of “stem cell therapies hazard ratios” did not identify a publication where the hazard ratio was less than 0.5. Furthermore, if the observed hazard ratio is smaller than 0.4, although possibly biased, the clinical decision that one treatment is superior would be unchanged if the expression for the proposed approximate UMVCUE is used to compute an estimate. An alternative to using the score statistic is to determine the asymptotic distribution of the log hazard ratio from the Cox’s proportional hazards model using techniques described by several authors.^26,46,47^ This would be expected to give similar results to those based on score statistics in most realistic trials where the treatment effects are not expected to be very big. However, in the instance with a very big treatment effect, the hazard ratio estimate based on the Cox’s model may be smaller than the one that is based on the score statistic. Also, the Cox model has the advantage of being able to incorporate covariates.^26^ Similarly, the upper bounds based on the score statistic distribution were observed to be conservative, which as with approximate UMVCUE will have little impact on trials. Using the Cox model to obtain the duality confidence intervals is straightforward because the stagewise *P*-values are obtained from stages 1 and 2 patients separately.

Since from our simulation study and previous work,^4–6,48^ the naive point estimator can have substantial bias, we rec-ommend using the approximate UMVCUE. The expression for the UMVCUE given by (3) is straightforward to implement. Also, the naive confidence intervals do not have the desired properties. Hence, we recommend the confidence intervals obtained by using the bounds of the simultaneous duality confidence intervals that we have developed when they are informative, and using the naive confidence intervals bounds when the bounds of the simultaneous duality confidence intervals are not informative. For example, using the results in Table 1, the confidence interval for the effect in partition 1 would be (−1.499, −0.077). It is straightforward to obtain the naive confidence intervals using expression (2). While demonstrating how to compute the duality confidence intervals in Section 4, we have written R functions to solve expressions (4) and (6) that can be used with any number of partitions. The code that includes the R functions is available in the supplementary material and we have also provided the key estimates to input in the functions to enable reproducing the worked example results.

## Supplementary Material

sup 01

## Figures and Tables

**TABLE 1:** Summary of the estimates from the constructed example

	Stage 1^a^	All data^a^	Increment^a^	UMVCUE $\left({\hat{\theta }}_{U_{i}}\right)$^a^		Confidence intervals	
	
	${\hat{\theta }}_{1,j}\left(\sigma_{1,j}^{2}\right)$	${\hat{\theta }}_{N_{j}}\left(\sigma_{N_{j}}^{2}\right)$	${\hat{\theta }}_{2,j}\left(\sigma_{2j}^{2}\right)$	AT^b^	IND^c^	Naive	Duality
Partition 1	−0.902 (0.191)	−0.746 (0.089)	−0.609 (0.167)	−0.737	(−0.631)	(−1.415, −0.077)	(−1.499, 0.000)
Partition 2	−0.419 (0.103)	−0.362 (0.053)	−0.301 (0.108)	−0.359	(−0.335)	(−0.876, 0.153)	(−0.911, 0.093)

a *j* = 1 for partition 1 and *j* = 2 for partition 2.

b AT=Adaptive threshold design.

c IND=Independently selecting partitions.

**TABLE 2:** Sample sizes for 1-year single-stage trials

Shape parameter (*γ*)	Control		Experimental		Required deaths	Required patients

	*λ_C_*	Median days	*λ_E_*	Median days
0.5	ln(2)∕20	400	ln(2)∕25	625	630	2060
1.0	ln(2)∕20^2^	400	ln(2)∕500	500	630	2600
1.5	ln(2)∕20^3^	400	ln(2)∕10^4^	465	630	3300

**TABLE 3:** Selection probabilities

True log hazard ratios	Ideal		Partitions selected				
(Configuration)	selection	Distribution	1, 2, 3, and 4	1, 2, and 3	1 and 2	1	Stop
*θ*_1_ = *θ*_2_= *θ*_3_ = *θ*_4_ =0.0198	Stop	*γ* = 0.5	0.4329	0.0876	0.0764	0.0812	**0.3219**
(Configuration 1)		*γ* = 1.0	0.4306	0.0866	0.0752	0.0841	**0.3235**
		*γ* = 1.5	0.4308	0.0872	0.0766	0.0834	**0.3220**
*θ*_1_ = −0.2231; *θ*_3_ = 0.3364;	1 and 2	*γ* = 0.5	0.1838	0.3022	**0.3387**	0.0756	0.0997
*θ*_2_ = −0.0953; *θ*_4_ = 0.4055		*γ* = 1.0	0.1830	0.3026	**0.3373**	0.0758	0.1014
(Configuration 2)		*γ* = 1.5	0.1850	0.3008	**0.3374**	0.0765	0.1003
*θ*_1_ = −0.4055; *θ*_3_ = −0.0953;	All	*γ* = 0.5	**0.9395**	0.0323	0.0146	0.0065	0.0070
*θ*_2_ = −0.2231; *θ*_4_ = 0.0000		*γ* = 1.0	**0.9389**	0.0332	0.0140	0.0068	0.0070
(Configuration 3)		*γ* = 1.5	**0.9386**	0.0334	0.0144	0.0065	0.0071

**TABLE 4:** Simulated biases and root mean squared errors of the estimators for the log hazard ratios (*γ* = 0.5)

		Simulated bias				Root mean squared error			
Selected		${\hat{\theta }}_{N_{j}}$		${\hat{\theta }}_{U_{j}}$		${\hat{\theta }}_{N_{j}}$		${\hat{\theta }}_{U_{j}}$	
		
partitions (S)	Partition	${\tilde{t}}_{1}>t_{1}$${\tilde{t}}_{1}=t_{1}$	${\tilde{t}}_{1}>t_{1}$	${\tilde{t}}_{1}=t_{1}$	${\tilde{t}}_{1}>t_{1}$	${\tilde{t}}_{1}=t_{1}$	${\tilde{t}}_{1}>t_{1}$	${\tilde{t}}_{1}=t_{1}$	${\tilde{t}}_{1}>t_{1}$
True log hazard ratios: *θ*_1_ = *θ*_2_ = *θ*_3_ = *θ*_4_ = 0.0198									
All	1	0.0423	0.0537	0.0142	0.0012	0.1469	0.1653	0.1514	0.1845
	2	0.0406	0.0518	0.0126	−0.0006	0.1466	0.1655	0.1513	0.1856
	3	0.0422	0.0531	0.0145	0.0012	0.1472	0.1654	0.1516	0.1846
	4	0.0419	0.0529	0.0142	0.0010	0.1472	0.1655	0.1515	0.1846
1, 2, and 3	1	0.0235	0.0289	0.0101	0.0050	0.1322	0.1455	0.1501	0.1939
	2	0.0192	0.0237	0.0039	−0.0048	0.1324	0.1450	0.1508	0.1947
	3	0.0221	0.0277	0.0075	0.0013	0.1314	0.1442	0.1502	0.1945
1 and 2	1	0.0235	0.0274	0.0074	0.0015	0.1181	0.1268	0.1339	0.1595
	2	0.0237	0.0278	0.0079	0.0022	0.1182	0.1266	0.1338	0.1584
1	1	0.0214	0.0241	0.0028	−0.0008	0.0927	0.0969	0.1043	0.1141
True log hazard ratios: *θ*_1_ = −0.2231, *θ*_2_ = −0.0953, *θ*_3_ = 0.3365, *θ*_4_ = 0.4055									
All	1	0.0765	0.0964	0.0271	0.0010	0.1676	0.1915	0.1656	0.2084
	2	0.0697	0.0889	0.0226	−0.0017	0.1602	0.1834	0.1600	0.2020
	3	0.0613	0.0767	0.0213	0.0004	0.1475	0.1681	0.1479	0.1855
	4	0.0597	0.0746	0.0209	0.0010	0.1462	0.1646	0.1460	0.1797
1, 2, and 3	1	0.0274	0.0334	0.0088	0.0002	0.1392	0.1532	0.1552	0.1948
	2	0.0265	0.0332	0.0086	0.0014	0.1367	0.1500	0.1516	0.1891
	3	0.0240	0.0295	0.0087	0.0026	0.1276	0.1389	0.1401	0.1712
1 and 2	1	−0.0098	−0.0114	−0.0017	0.0015	0.1207	0.1295	0.1353	0.1573
	2	−0.0107	−0.0123	−0.0030	−0.0003	0.1190	0.1276	0.1332	0.1540
1	1	−0.0128	−0.0138	−0.0008	0.0019	0.0942	0.0983	0.1059	0.1149
True log hazard ratios: *θ*_1_ = −0.4055, *θ*_2_ = −0.2231, *θ*_3_ = −0.0953, *θ*_4_ = 0									
All	1	0.0056	0.0063	0.0010	−0.0018	0.1504	0.1681	0.1534	0.1752
	2	0.0062	0.0074	0.0020	0.0000	0.1460	0.1633	0.1485	0.1694
	3	0.0068	0.0085	0.0027	0.0013	0.1426	0.1592	0.1451	0.1653
	4	0.0057	0.0072	0.0017	0.0004	0.1401	0.1567	0.1426	0.1624
1, 2, and 3	1	−0.0671	−0.0805	−0.0245	−0.0014	0.1498	0.1658	0.1573	0.2004	
	2	−0.0624	−0.0769	−0.0221	−0.0038	0.1454	0.1619	0.1519	0.1928
	3	−0.0574	−0.0678	−0.0189	0.0035	0.1396	0.1551	0.1468	0.1860
1 and 2	1	−0.0652	−0.0769	−0.0190	−0.0033	0.1348	0.1488	0.1363	0.1613
	2	−0.0611	−0.0730	−0.0167	−0.0030	0.1291	0.1414	0.1306	0.1532
1	1	−0.0471	−0.0523	−0.0109	−0.0046	0.1001	0.1044	0.1007	0.1067

**TABLE 5:** Coverage probability and type I error rate (Weibull distribution, *γ* = 0.5)

	Selected	Coverage (Type I error rate)^a^	
True log hazard ratios	partitions (S)	Naive	Duality
	All	94.8 (4.5)	98.3 (1.7)
*θ*_1_ = *θ*_2_ = *θ*_3_ = *θ*_4_ =0.0198	1, 2, and 3	96.1 (3.0)	99.1 (0.9)
	1 and 2	96.0 (3.1)	99.2 (0.8)
	1	96.3 (2.9)	99.6 (0.4)
	All	93.0 (6.6)	98.4 (1.5)
*θ*_1_ = −0.2231, *θ*_2_ = −0.0953,	1, 2, and 3	96.0 (3.1)	99.1 (0.5)
*θ*_3_ = 0.3365, *θ*_4_ = 0.4055	1 and 2	96.3 (1.4)	99.2 (0.0)
	1	96.1 (1.5)	99.4 (0.0)
	All	95.4 (2.6)	98.7 (0.7)
*θ*_1_ = −0.4055, *θ*_2_ = −0.2231,	1, 2, and 3	93.3 (0.3)	97.6 (0.0)
*θ*_3_ = −0.0953, *θ*_4_ = 0	1 and 2	93.8 (0.7)	98.2 (0.0)
	1	94.0 (0.9)	98.6 (0.0)

a Type I error is the probability that at least one upper bound is less than the true value.
