# Supplementary material for: Point and interval estimation in two-stage adaptive designs with time to event data and biomarker-driven subpopulation selection
Source: Stat Med. Author manuscript; Available in PMC 2021 Aug 30. (PMC7785132; doi:10.1002/sim.8557)
Supplement: sup 01 [file NIHMS1649347-supplement-sup_01.pdf]

Point and interval estimation in two-stage adaptive designs with time to event data and biomarker-driven subpopulation selection

Supplementary material

1 Extracting the estimates using R

The estimates described in Section 2.2 of the main paper can be extracted from statistical packages. For example for stage 1 estimate $\hat{\theta}_{1,j}$, in R [1], the output of the function "survdiff" in the "survival" package includes the Fisher information $V_{1,j}$ and the chi-squared test statistic $T_{1,j} = S_{1,j}^2/V_{1,j}$ that corresponds to the log-rank test. Hence, when the expected deaths (E) are more than the observed deaths (O) in the experimental arm, $\hat{\theta}_{1,j} = -\sqrt{T_{1,j}/V_{1,j}}$ and $S_{1,j} = -\sqrt{T_{1,j}V_{1,j}}$ while when $E \leq O$, $\hat{\theta}_{1,j} = \sqrt{T_{1,j}/V_{1,j}}$ and $S_{1,j} = \sqrt{T_{1,j}V_{1,j}}$. This is demonstrated with a worked example in Section 5 of this document.

2 Deriving the approximate asymptotic uniformly minimum variance conditional estimator

We only give the sketch for the derivation as most steps follow from several papers [2–8]. Let $Q_{\mathcal{S}}$ denote the event of selecting the subset $\mathcal{S} \subseteq \{1,...,K\}$. Without loss of generality, suppose that $\mathcal{S} = \{1,2\}$. We derive the asymptotic uniformly minimum variance conditional unbiased estimator (UMVCUE) for θ_1. The UMVCUE for θ_2 can be derived similarly. When a density for outcome X is conditional on $Q_{\mathcal{S}}$, we use the convention $f_{Q_{\mathcal{S}}}(x)$. The joint density for the data collected before and after the interim analysis that are used for the estimation is $f_{Q_{\mathcal{S}}}(\hat{\theta}_{1,1}, \hat{\theta}_{1,2}, \hat{\theta}_{1,3}..., \hat{\theta}_{1,K}, \hat{\theta}_{2,1}, \hat{\theta}_{2,2})$ and can be expressed as

$$\frac{1}{\sigma_{1,1}}\phi\left(\frac{\hat{\theta}_{1,1}-\theta_1}{\sigma_{1,1}}\right)\frac{1}{\sigma_{1,2}}\phi\left(\frac{\hat{\theta}_{1,2}-\theta_2}{\sigma_{1,2}}\right)\frac{1}{\sigma_{1,3}}\phi\left(\frac{\hat{\theta}_{1,3}-\theta_3}{\sigma_{1,3}}\right)\times\cdots\times\frac{1}{\sigma_{1,K}}\phi\left(\frac{\hat{\theta}_{1,K}-\theta_K}{\sigma_{1,K}}\right)\times$$
$$\frac{1}{\sigma_{2,1}}\phi\left(\frac{\hat{\theta}_{2,1}-\theta_1}{\sigma_{2,1}}\right)\frac{1}{\sigma_{2,2}}\phi\left(\frac{\hat{\theta}_{2,2}-\theta_2}{\sigma_{2,2}}\right)\frac{\mathbf{1}[Q_{\mathcal{S}}]}{P[Q_{\mathcal{S}}]}, \quad (1)$$

where $\mathbf{1}[Q_{\mathbb{S}}]$ and $P[Q_{\mathbb{S}}]$ are respectively the indicator and probability for the event $Q_{\mathbb{S}}$. It can be re-expressed as

$$\frac{1}{\sigma_{2,1}}\phi\left(\frac{\frac{\sigma_{2,1}}{\sigma_{1,1}}\hat{\theta}_{1,1}+\frac{\sigma_{1,1}}{\sigma_{2,1}}\hat{\theta}_{2,1}-\theta_1\alpha_1}{\sqrt{\sigma_{1,1}^2+\sigma_{2,1}^2}}\right)\frac{1}{\sigma_{1,1}}\phi\left(\frac{\hat{\theta}_{1,1}-\left(\frac{\sigma_{2,1}}{\sigma_{1,1}}\hat{\theta}_{1,1}+\frac{\sigma_{1,1}}{\sigma_{2,1}}\hat{\theta}_{2,1}\right)/\alpha_1}{\frac{\sigma_{1,1}^2}{\sigma_{2,1}^2}\alpha_2}\right)A\frac{\mathbf{1}[Q_{\mathbb{S}}]}{P[Q_{\mathbb{S}}]}, \tag{2}$$

where $\alpha_1=\frac{\sigma_{1,1}}{\sigma_{2,1}}+\frac{\sigma_{2,1}}{\sigma_{1,1}}=$, $\alpha_2=\frac{\sigma_{2,1}^2}{\sqrt{\sigma_{1,1}^2+\sigma_{2,1}^2}}$ and

$$A=\frac{1}{\sigma_{1,2}}\phi\left(\frac{\hat{\theta}_{1,2}-\theta_2}{\sigma_{1,2}}\right)\frac{1}{\sigma_{1,3}}\phi\left(\frac{\hat{\theta}_{1,3}-\theta_3}{\sigma_{1,3}}\right)\times\cdots\times\frac{1}{\sigma_{1,K}}\phi\left(\frac{\hat{\theta}_{1,K}-\theta_K}{\sigma_{1,K}}\right)\frac{1}{\sigma_{2,2}}\phi\left(\frac{\hat{\theta}_{2,2}-\theta_2}{\sigma_{2,2}}\right).$$

Let $\tilde{\theta}_1=\frac{\sigma_{2,1}}{\sigma_{1,1}}\hat{\theta}_{1,1}+\frac{\sigma_{1,1}}{\sigma_{2,1}}\hat{\theta}_{2,1}$. Conditional on $Q_{\mathbb{S}}$, the vector $\left(\tilde{\theta}_1,\hat{\theta}_{1,2},\hat{\theta}_{1,3}...,\hat{\theta}_{1,K},\hat{\theta}_{2,2}\right)$ is sufficient and complete for estimating $(\theta_1,\theta_2,...,\theta_K)$. Therefore, since $\hat{\theta}_{2,1}$ is unbiased for θ_1, the UMVCUE for θ_1 is the expression for $E\left[\hat{\theta}_{2,1}|\tilde{\theta}_1,\hat{\theta}_{1,2},\hat{\theta}_{1,3}...,\hat{\theta}_{1,K},\hat{\theta}_{2,2},Q_{\mathbb{S}}\right]$. To get the expression we require the density

$$f_{Q_{\mathbb{S}}}\left(\hat{\theta}_{2,1}|\tilde{\theta}_1,\hat{\theta}_{1,2},\hat{\theta}_{1,3}...,\hat{\theta}_{1,K},\hat{\theta}_{2,2}\right)=\frac{f_{Q_{\mathbb{S}}}\left(\hat{\theta}_{2,1},\tilde{\theta}_1,\hat{\theta}_{1,2},\hat{\theta}_{1,3}...,\hat{\theta}_{1,K},\hat{\theta}_{2,2}\right)}{f_{Q_{\mathbb{S}}}\left(\tilde{\theta}_1,\hat{\theta}_{1,2},\hat{\theta}_{1,3}...,\hat{\theta}_{1,K},\hat{\theta}_{2,2}\right)}. \tag{3}$$

The numerator in expression (3) is obtained by transforming the density given by expression (1). Density (1) is first re-expressed to a form similar to density (2) and then using transformation of random variables techniques which gives

$$f_{Q_{\mathbb{S}}}\left(\hat{\theta}_{2,1},\tilde{\theta}_1,\hat{\theta}_{1,2},\hat{\theta}_{1,3}...,\hat{\theta}_{1,K},\hat{\theta}_{2,2},Q_{\mathbb{S}}\right)=\phi\left(\frac{\tilde{\theta}_1-\theta_1\alpha_1}{\sqrt{\sigma_{1,1}^2+\sigma_{2,1}^2}}\right)\frac{1}{\sigma_{2,1}^2}\phi\left(\frac{\hat{\theta}_{2,1}-\frac{\tilde{\theta}_1}{\alpha_1}}{\alpha_2}\right)A\frac{\mathbf{1}[Q_{\mathbb{S}}]}{P[Q_{\mathbb{S}}]}. \tag{4}$$

The denominator in expression (3) is obtained by transforming the density given by expression (1) to obtain the density $f_{Q_{\mathbb{S}}}\left(\hat{\theta}_{1,1},\tilde{\theta}_1,\hat{\theta}_{1,2},\hat{\theta}_{1,3}...,\hat{\theta}_{1,K},\hat{\theta}_{2,2}\right)$ and then integrating out $\hat{\theta}_{1,1}$. Using transformation of random variables, the density $f_{Q_{\mathbb{S}}}\left(\hat{\theta}_{1,1},\tilde{\theta}_1,\hat{\theta}_{1,2},\hat{\theta}_{1,3}...,\hat{\theta}_{1,K},\hat{\theta}_{2,2}\right)$ follows from expression (2) (a re-expression of the density given by (1)) and is given by

$$f_{Q_{\mathbb{S}}}\left(\hat{\theta}_{1,1},\tilde{\theta}_1,\hat{\theta}_{1,2},\hat{\theta}_{1,3}...,\hat{\theta}_{1,K},\hat{\theta}_{2,2}\right)=\phi\left(\frac{\tilde{\theta}_1-\theta_1\alpha_1}{\sqrt{\sigma_{1,1}^2+\sigma_{2,1}^2}}\right)\frac{1}{\sigma_{1,1}^2}\phi\left(\frac{\hat{\theta}_{1,1}-\frac{\tilde{\theta}_1}{\alpha_1}}{\frac{\sigma_{1,1}^2}{\sigma_{2,1}^2}\alpha_2}\right)A\frac{\mathbf{1}[Q_{\mathbb{S}}]}{P[Q_{\mathbb{S}}]}. \tag{5}$$

Thus the denominator in expression (3), $f_{Q_{\mathbb{S}}}\left(\tilde{\theta}_1,\hat{\theta}_{1,2},\hat{\theta}_{1,3}...,\hat{\theta}_{1,K},\hat{\theta}_{2,2}\right)$, is obtained by integrating out $\tilde{\theta}_{1,1}$ in expression (5) as follows.

$$A\frac{\mathbf{1}[Q_{\mathbb{S}}]}{P[Q_{\mathbb{S}}]}\phi\left(\frac{\tilde{\theta}_1-\theta_1\alpha_1}{\sqrt{\sigma_{1,1}^2+\sigma_{2,1}^2}}\right)\int_{l_1}^{w_1}\phi\left(\frac{\hat{\theta}_{1,1}-\frac{\tilde{\theta}_1}{\alpha_1}}{\frac{\sigma_{1,1}^2}{\sigma_{2,1}^2}\alpha_2}\right)d\hat{\theta}_{1,1}, \tag{6}$$

where l_1 and w_1 are expressions for the bounds described in Section 4 and in the main paper in Section 3. Note that it is while integrating that we account for the selection rule since the expressions for l_1 and w_1 are based on the selection rule and observed data. Solving the integral in expression (6), the expression for $f_{Q_{\mathbb{S}}}\left(\tilde{\theta}_1, \hat{\theta}_{1,2}, \hat{\theta}_{1,3}..., \hat{\theta}_{1,K}, \hat{\theta}_{2,2}\right)$ is

$$A \frac{\mathbf{1}[Q_{\mathbb{S}}]}{P[Q_{\mathbb{S}}]} \frac{\alpha_2}{\sigma_{2,1}^2} \phi\left(\frac{\tilde{\theta}_1 - \theta_1\alpha_1}{\sqrt{\sigma_{1,1}^2 + \sigma_{2,1}^2}}\right) \times$$

$$\left[\Phi\left\{\frac{\sqrt{\sigma_{1,1}^2 + \sigma_{2,1}^2}}{\sigma_{1,1}^2}\left(w_1 - \hat{\theta}_{N_1}\right)\right\} - \Phi\left\{\frac{\sqrt{\sigma_{1,1}^2 + \sigma_{2,1}^2}}{\sigma_{1,1}^2}\left(l_1 - \hat{\theta}_{N_1}\right)\right\}\right], \tag{7}$$

where

$$\hat{\theta}_{N_1} = \frac{\sigma_{2,1}^2 \hat{\theta}_{1,1} + \sigma_{1,1}^2 \hat{\theta}_{2,1}}{\sigma_{1,1}^2 + \sigma_{2,1}^2}.$$

From expressions (3), (4) and (7), the expression for $f_{Q_{\mathbb{S}}}\left(\hat{\theta}_{2,1} | \tilde{\theta}_1, \hat{\theta}_{1,2}, \hat{\theta}_{1,3}..., \hat{\theta}_{1,K}, \hat{\theta}_{2,2}\right)$ is

$$\frac{\frac{1}{\alpha_2}\phi\left(\frac{\hat{\theta}_{2,1} - \frac{\tilde{\theta}_1}{\alpha_1}}{\alpha_2}\right)}{\Phi\{g(l_1)\} - \Phi\{g(w_1)\}} I\left[\frac{\sigma_{2,1}}{\sigma_{1,1}}\left(\tilde{\theta}_1 - \frac{\sigma_{2,1}}{\sigma_{1,1}} w_1\right) < \hat{\theta}_{2,1} < \frac{\sigma_{2,1}}{\sigma_{1,1}}\left(\tilde{\theta}_1 - \frac{\sigma_{2,1}}{\sigma_{1,1}} l_1\right)\right], \tag{8}$$

where $I[x]$ denotes an indicator for event x and for $x \in \{l_1, w_1\}$,

$$g(x) = \left\{\frac{\sqrt{\sigma_{1,1}^2 + \sigma_{2,1}^2}}{\sigma_{1,1}^2}\left(\hat{\theta}_{N_1} - x\right)\right\}.$$

It follows that the UMVCUE for θ_1 is

$$\frac{1}{\Phi\{g(l_1)\} - \Phi\{g(w_1)\}} \int_b^a \frac{\hat{\theta}_{2,1}}{\alpha_2} \phi\left(\frac{\hat{\theta}_{2,1} - \frac{\tilde{\theta}_1}{\alpha_1}}{\alpha_2}\right) d\hat{\theta}_{2,1},$$

where $a = \frac{\sigma_{2,1}}{\sigma_{1,1}}\left(\tilde{\theta}_1 - \frac{\sigma_{2,1}}{\sigma_{1,1}} l_1\right)$ and $b = \frac{\sigma_{2,1}}{\sigma_{1,1}}\left(\tilde{\theta}_1 - \frac{\sigma_{2,1}}{\sigma_{1,1}} w_1\right)$. The integral can be solved as done in several papers [3, 4, 7, 9], which gives the simplified expression for the UMVCUE for θ_1 as

$$\hat{\theta}_{U_1} = \hat{\theta}_{N_1} - \frac{\sigma_{2,1}^2}{\sqrt{\sigma_{2,1}^2 + \sigma_{2,1}^2}} \frac{\phi(g(l_1)) - \phi(g(w_1))}{\Phi(g(l_1)) - \Phi(g(w_1))}.$$

3 More selection rules and details for the expressions of the bounds

The expressions for the bounds are a function of the selection rule, selected partitions and stage 1 estimates. However, since for a trial a single subset $\mathcal{S}$ is selected, for each $j \in \mathcal{S}$, we simplified notation for the bounds to be l_j and w_j rather than, for example, $l_{j,\mathcal{S}}$ and $w_{j,\mathcal{S}}$. As examples, we describe the expressions for l_j and w_j for the selection rules in Section 2.3 in the main paper.

For $K = 2$, the points corresponding to the expressions for l_j and w_j are illustrated in Figure S1. While estimating θ_1, the lower and upper edges of the vertical dashed and dotted lines that go through the stage 1 estimates correspond to l_1 (lower bound for $\hat{\theta}_{1,1}$) and w_1 (upper bound for $\hat{\theta}_{1,1}$), respectively. For estimating θ_2, the right hand and left hand edges of the horizontal lines that go through the stage 1 estimates correspond to l_2 (lower bound for $\hat{\theta}_{1,2}$) and w_2 (upper bound for $\hat{\theta}_{1,2}$), respectively. Consequently from Figure S1(a), for $K = 2$, for the adaptive threshold enrichment design, when only partition 1 is selected, $l_1 = (b - p_2\hat{\theta}_{1,2})/p_1$ and $w_1 = b$, while when both partitions are selected $l_1 = -\infty$, $w_1 = (b - p_2\hat{\theta}_{1,2})/p_1$, $l_2 = -\infty$ and $w_2 = (b - p_1\hat{\theta}_{1,1})/p_2$. Let $p'_j = \sum_{i=1}^{j} p_i$ $(j = 1, ..., K)$. For the adaptive threshold enrichment design, we show in the next section that for any $K \geq 2$, when a subpopulation consisting of s $(s = 1, ..., K)$ partitions is selected, for each $j \in \{1, ..., s\} = \mathcal{S}$, $w_j = \left(p'_s b - \sum_{\substack{i=1 \\ i \neq j}}^{s} p_i\hat{\theta}_{1,i}\right) \Big/ p_j$ (the term $\sum_{\substack{i=1 \\ i \neq j}}^{s} p_i\hat{\theta}_{1,i}$ is set to zero when $s = 1$) and

$$l_j = \max\left\{ \frac{p'_{s+1}b - \sum_{\substack{i=1 \\ i \neq j}}^{s+1} p_i\hat{\theta}_{1,i}}{p_j}, \frac{p'_{s+2}b - \sum_{\substack{i=1 \\ i \neq j}}^{s+2} p_i\hat{\theta}_{1,i}}{p_j}, ..., \frac{p'_K b - \sum_{\substack{i=1 \\ i \neq j}}^{K} p_i\hat{\theta}_{1,i}}{p_j} \right\},$$

with l_j set to be $-\infty$ if all partitions are selected.

From Figure S1(b), for the second selection rule in Section 2.3 in the main paper where all partitions whose stage 1 estimates are $\leq b$ are selected, for any $\mathcal{S} \subseteq \{1, ..., K\}$, for each $j \in \mathcal{S}$, $l_j = -\infty$ and $w_j = b$.

Kimani *et al.* [7] consider the case of two partitions, that is $K = 2$, and where the trial always continues to stage 2. Their selection rule assumes a monotonic relationship between the biomarker values and the treatment effect so that the trial continues to stage 2 with partition 1 only or F. When the selection rule is modified so that a smaller estimate is desired, partition 1 is selected if $\hat{\theta}_{1,1} < \hat{\theta}_{1,2} - m/p_2$, where m is a pre-specified positive number. Otherwise the trial continues with F. The decision regions when $p_1 = p_2$ are shown in Figure S1(c). For the case of selecting partition 1, $l_1 = -\infty$ and $w_1 = \hat{\theta}_{1,2} - m/p_2$. For the case of selecting F, $l_1 = \hat{\theta}_{1,2} - m/p_2$, $w_1 = \infty$, $l_2 = -\infty$ and $w_2 = \hat{\theta}_{1,1} + m/p_2$.

Kunzmann *et al.* [10] selection rule replace m/p_2 in the Kimani *et al.* selection rule with a function $d_\alpha(\cdot)$ that encompasses a class of selection rules such as using stage 1 p-value(s) for selection, with the Kimani *et al.* rule being a special case. Consequently, for Kunzmann *et al.* selection rule, we replace m/p_2 with $d_\alpha(\cdot)$ in l_1, l_2, w_1 and w_2 defined for Kimani *et al.*.

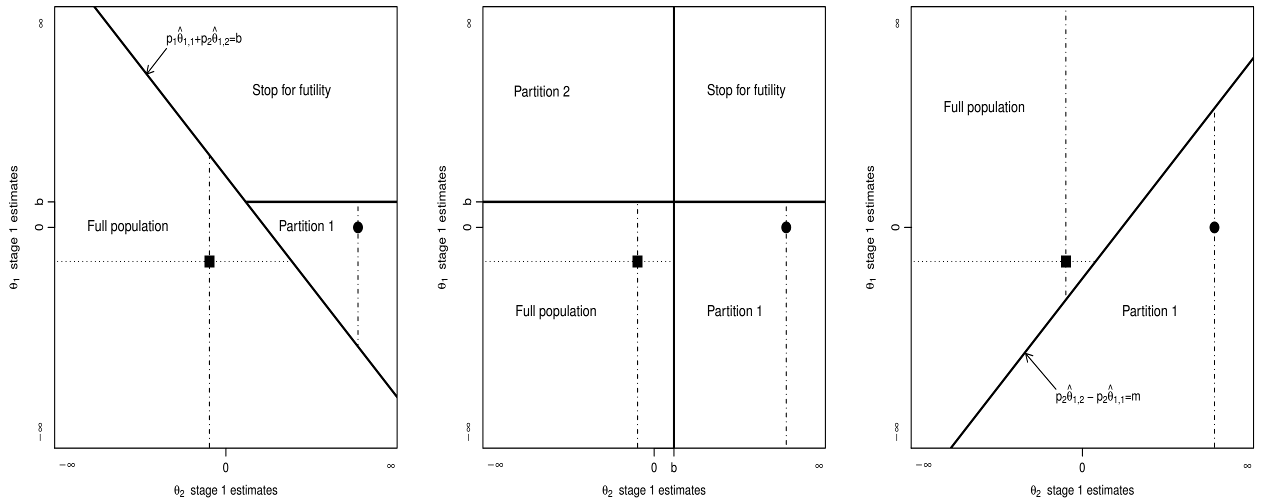

(a) Adaptive threshold enrichment design (b) Selecting partitions independently (c) Kimani et al. (2015) selection rule

Figure S1: Decision regions for two selection rules when $K = 2$. The continuous lines are the decision boundaries. The filled circle and square are two possible stage 1 results that lead to selecting partition 1 and F, respectively. The edges of the vertical dashed and dotted lines give the bounds for estimating θ_1. The edges of the horizontal dashed lines give the bounds for estimating θ_2.

4 The bounds for the adaptive threshold enrichment design

The case of selecting the full population (K partitions)

The full population (F) is selected if $p_1\hat{\theta}_{1,1} + p_2\hat{\theta}_{1,2} + \cdots + p_K\hat{\theta}_{1,K} \leq b$ which implies that for j ($j = 1, ..., K$)

$$\hat{\theta}_{1,j} \leq \frac{b - \sum_{\substack{i=1\\ i\neq j}}^{K} p_i\hat{\theta}_{1,i}}{p_j}.$$

When F is selected, there is no lower bound for $p_1\hat{\theta}_{1,1} + p_2\hat{\theta}_{1,2} + \cdots + p_K\hat{\theta}_{1,K}$. Therefore, when F is selected, $l_j < \hat{\theta}_{1,j} \leq w_j$ ($j = 1, ..., K$), where $l_j = -\infty$ and

$$w_j = \frac{b - \sum_{\substack{i=1\\ i\neq j}}^{K} p_i\hat{\theta}_{1,i}}{p_j}.$$

The case of selecting the subpopulation consisting of $K-1$ partitions

The subpopulation consisting of $K-1$ partitions is selected if the following two conditions are satisfied

- $p_1\hat{\theta}_{1,1} + p_2\hat{\theta}_{1,2} + \cdots + p_K\hat{\theta}_{1,K} > b$.
- $\frac{p_1\hat{\theta}_{1,1} + p_2\hat{\theta}_{1,2} + \cdots + p_{K-1}\hat{\theta}_{1,K-1}}{p'_{K-1}} \leq b$, where $p'_{K-1} = \sum_{i=1}^{K-1} p_i$.

For a selected partition j $(j = 1, ..., K-1)$, the first condition implies that

$$\hat{\theta}_{1,j} > \frac{b - \sum_{\substack{i=1 \\ i \neq j}}^{K} p_i \hat{\theta}_{1,i}}{p_j}$$

and the second condition implies that

$$\hat{\theta}_{1,j} \leq \frac{p'_{K-1} b - \sum_{\substack{i=1 \\ i \neq j}}^{K-1} p_i \hat{\theta}_{1,i}}{p_j}.$$

Hence, when $K-1$ partititions are selected, $l_j < \hat{\theta}_{1,j} \leq w_j$ $(j = 1, ..., K-1)$, where

$$l_j = \frac{b - \sum_{\substack{i=1 \\ i \neq j}}^{K} p_i \hat{\theta}_{1,i}}{p_j} \quad \text{and} \quad w_j = \frac{p'_{K-1} b - \sum_{\substack{i=1 \\ i \neq j}}^{K-1} p_i \hat{\theta}_{1,i}}{p_j}.$$

The case of selecting the subpopulation consisting of $K-2$ partitions

The subpopulation consisting of $K-2$ partitions is selected if the following three conditions are satisfied

- $p_1 \hat{\theta}_{1,1} + p_2 \hat{\theta}_{1,2} + \cdots + p_K \hat{\theta}_{1,K} > b.$
- $\frac{p_1 \hat{\theta}_{1,1} + p_2 \hat{\theta}_{1,2} + \cdots + p_{K-1} \hat{\theta}_{1,K-1}}{p'_{K-1}} > b$, where $p'_{K-1} = \sum_{i=1}^{K-1} p_i$.
- $\frac{p_1 \hat{\theta}_{1,1} + p_2 \hat{\theta}_{1,2} + \cdots + p_{K-2} \hat{\theta}_{1,K-2}}{p'_{K-2}} \leq b$, where $p'_{K-2} = \sum_{i=1}^{K-2} p_i$.

For a selected partition j $(j = 1, ..., K-2)$, the first condition implies that

$$\hat{\theta}_{1,j} > \frac{b - \sum_{\substack{i=1 \\ i \neq j}}^{K} p_i \hat{\theta}_{1,i}}{p_j},$$

the second condition implies that

$$\hat{\theta}_{1,j} > \frac{p'_{K-1} b - \sum_{\substack{i=1 \\ i \neq j}}^{K-1} p_i \hat{\theta}_{1,i}}{p_j}$$

and the third condition implies that

$$\hat{\theta}_{1,j} \leq \frac{p'_{K-2} b - \sum_{\substack{i=1 \\ i \neq j}}^{K-2} p_i \hat{\theta}_{1,i}}{p_j}.$$

From the three conditions, when $K-2$ partitions are selected, $l_j < \hat{\theta}_{1,j} \leq w_j$ $(j = 1, ..., K-2)$, where

$$l_j = \max \left\{ \frac{b - \sum_{\substack{i=1 \\ i \neq j}}^{K} p_i \hat{\theta}_{1,i}}{p_j}, \frac{p'_{K-1} b - \sum_{\substack{i=1 \\ i \neq j}}^{K-1} p_i \hat{\theta}_{1,i}}{p_j} \right\} \quad \text{and} \quad w_j = \frac{p'_{K-2} b - \sum_{\substack{i=1 \\ i \neq j}}^{K-2} p_i \hat{\theta}_{1,i}}{p_j}.$$

The case of selecting the subpopulation consisting of s partitions

Let $p'_j = \sum_{i=1}^{j} p_i$ $(j = 1,...,K)$. In general, following the above description of how to obtain bounds for the cases of F, $K-1$ partitions and $K-2$ partitions, it can be seen that when the subpopulation consisting of s $(s \in \{1,...,K\})$ partitions is selected, $l_j < \hat{\theta}_{1,j} \leq w_j$ $(j = 1,...,s)$ where

$$l_j = \max\left\{ \frac{p'_{s+1}b - \sum_{\substack{i=1 \\ i\neq j}}^{K-2} p_i\hat{\theta}_{1,i}}{p_j}, \frac{p'_{s+2}b - \sum_{\substack{i=1 \\ i\neq j}}^{s+2} p_i\hat{\theta}_{1,i}}{p_j}, ..., \frac{p'_K b - \sum_{\substack{i=1 \\ i\neq j}}^{K} p_i\hat{\theta}_{1,i}}{p_j} \right\}$$

and

$$w_j = \frac{p'_s b - \sum_{\substack{i=1 \\ i\neq j}}^{s} p_i\hat{\theta}_{1,i}}{p_j}.$$

5 Worked example

5.1 Format of the data

Table S1 shows the template of how the data may be formatted. We created a dataset with variables “Group” (1 for C-MTX and 0 for HDMTX), “Partition” (1 for high risk patients and 2 for low and intermediate risk patients) and “Stage” (1 and 2 for stages 1 and 2 patients, respectively). The dataset also includes the variables “SurvTime1” and “Status1” which are the survival time and censoring status (1 for DFS event and 0 for no DFS event) for stage 1 patients 2176 days after the first enrolment (t_1 is the date corresponding to this day). The other key variables are ”SurvTime2” and “Status2” which are the survival time and censoring status (1 for DFS event and 0 for no DFS event) 3455 days after the first enrolment for stage 1 patients ($\tilde{t}_1$ is the date corresponding to this day) and 3644 days after the first enrolment for stage 2 patients (t_2 is the date corresponding to this day).

We named the dataset “WinterEtAllData”.

5.2 R code used to compute estimates

5.2.1 Loading the required package

```
library(survival)
```

5.2.2 Creating a copy of the dataset

```
TALLdata <- WinterEtAllData
```

5.2.3 Stage 1 estimates

```
TALLdata$SurvTime <- with(TALLdata, Surv(SurvTime1, Status1)) # Specifying survival time and status variables (Interim analysis)
```

Partition 1 estimates

```
TALLdata1 <- TALLdata[TALLdata$Partition==1,] # Selecting Partition 1 patients
a <- survdiff(SurvTime1 ~ Group, data=TALLdata1, rho = 0) # Log rank test
```

Table S1: Template dataset

PatID	EnrolDay	Group	Partition	Stage	SurvTime1	Status1	SurvTime2	Status2
1	1	0	2	1	2175	0	2957	1
2	2	1	2	1	689	1	689	1
3	2	0	2	1	1836	1	1836	1
4	2	1	2	1	2174	0	3453	0
5	7	0	1	1	2169	0	3448	0
6	10	1	1	1	1361	1	1361	1
7	12	0	2	1	741	1	741	1
8	12	1	2	1	2164	0	3224	1
9	13	0	2	1	964	1	964	1
10	14	1	2	1	2162	0	3630	0
11	2178	0	2	2	·	·	1466	0
12	2178	1	2	2	·	·	1138	1
13	2179	0	2	2	·	·	897	1
14	2183	1	2	2	·	·	1461	0
15	2187	0	1	2	·	·	1457	0
16	2188	1	1	2	·	·	1235	1
17	2191	0	2	2	·	·	1453	0
18	2192	1	2	2	·	·	987	1
19	2196	0	2	2	·	·	1448	0
20	2199	1	2	2	·	·	1189	1

Indic1 <- ((a$exp[2]-a$obs[2])>0) # Indicator that the expected deaths are more than observed deaths

Indic2 <- # Indicator that the expected deaths are less or equal to the observed deaths

P1information <- a$var[2,2] # Partition 1 Fisher information ($V_{1,1}$)

round(P1information, 3)

5.242

P1estimate <- Indic1*-1*sqrt(a$chisq / P1information) + Indic2*sqrt(a$chisq / P1information) # Partition 1 stage 1 estimate ($\hat{\theta}_{1,1}$)

round(P1estimate, 3)

-0.902

P1var <- 1/P1information # Variance of Partition 1 estimate ($\sigma^2_{1,1}$).

round(P1var, 3)

0.191

<u>Partition 2 estimates</u>

TALLdata2 <- TALLdata[TALLdata$Partition==2,] # Selecting Partition 2 patients

a <- survdiff(SurvTime1 ∼ Group, data=TALLdata2, rho = 0) # Log rank test

Indic1 <- ((a$exp[2]-a$obs[2])>0) # Indicator that the expected deaths are more than observed deaths

Indic2 <- # Indicator that the expected deaths are less or equal to the observed deaths

P2information <- a$var[2,2] # Partition 2 Fisher information ($V_{1,2}$)

round(P2information, 3)

9.739

P2estimate <- Indic1*-1*sqrt(a$chisq / P2information) + Indic2*sqrt(a$chisq / P2information) # Partition 2 stage 1 estimate ($\hat{\theta}_{1,2}$)

round(P2estimate, 3)

-0.419

P2var <- 1/P2information # Variance of Partition 2 estimate ($\sigma^2_{1,2}$).

round(P2var, 3)

0.103

5.2.4 Naive estimates

TALLdata$SurvTime <- with(TALLdata, Surv(SurvTime2, Status2)) # Specifying survival time and status variables (Stage 2 analysis)

<u>Partition 1</u>

TALLdata1 <- TALLdata[TALLdata$Partition==1,] # Selecting Partition 1 patients

a <- survdiff(SurvTime1 ~ Group, data=TALLdata1, rho = 0) # Log rank test

Indic1 <- ((a$exp[2]-a$obs[2])>0) # Indicator that the expected deaths are more than observed deaths

Indic2 <- # Indicator that the expected deaths are less or equal to the observed deaths

P1informationAll <- a$var[2,2] # Partition 1 Fisher information (V_{N_1})

round(P1informationAll, 3)

11.217

P1naive.estimate <- Indic1*-1*sqrt(a$chisq / P1informationAll) + Indic2*sqrt(a$chisq / P1informationAll) # Partition 1 naive estimate ($\hat{\theta}_{N_1}$)

round(P1naive.estimate, 3)

-0.746

<u>Partition 2</u>

TALLdata2 <- TALLdata[TALLdata$Partition==2,] # Selecting Partition 2 patients

a <- survdiff(SurvTime1 ~ Group, data=TALLdata2, rho = 0) # Log rank test

Indic1 <- ((a$exp[2]-a$obs[2])>0) # Indicator that the expected deaths are more than observed deaths

Indic2 <- # Indicator that the expected deaths are less or equal to the observed deaths

P2informationAll <- a$var[2,2] # Partition 2 Fisher information (V_{N_2})

round(P2informationAll, 3)

18.983

P2naive.estimate <- Indic1*-1*sqrt(a$chisq / P2informationAll) + Indic2*sqrt(a$chisq / P2informationAll) # Partition 2 stage 1 estimate ($\hat{\theta}_{N_2}$)

round(P2naive.estimate, 3)

-0.362

5.2.5 Naive confidence intervals

<u>Partition 1</u>

P1naive.lower <- P1naive.estimate - (qnorm(1 - (0.05/4)) * sqrt(1/P1informationAll)) # Partition 1 lower bound

round(P1naive.lower, 3)

-1.415

P1naive.upper <- P1naive.estimate + (qnorm(1 - (0.05/4)) * sqrt(1/P1informationAll)) # Partition 1 upper bound

round(P1naive.upper, 3)

-0.077

<u>Partition 2</u>

P2naive.lower <- P2naive.estimate - (qnorm(1 - (0.05/4)) * sqrt(1/P2informationAll)) # Partition 2 lower bound

round(P2naive.lower, 3)

-0.876

P2naive.upper <- P2naive.estimate + (qnorm(1 - (0.05/4)) * sqrt(1/P2informationAll)) # Partition 2 upper bound

round(P2naive.upper, 3)

0.153

5.2.6 Approximate UMVCUE estimates

Quantities and estimates common to any selection rule

b <- 0 # Futility boundary

P1prev <- 0.2 # Partition 1 prevalence (p_1)

P2prev <- 0.8 # Partition 2 prevalence (p_2)

P1var1 <- 1/(P1informationAll - P1information) # Variance of Partition 1 increment estimate ($\sigma^2_{2,1} = 1/(V_{N_1} - V_{1,1})$).

P1var11 <- P1var1 / sqrt(P1var+P1var1) # Partition 1 second term in equation (3) in main paper $\left(\frac{\sigma^2_{2,1}}{\sqrt{\sigma^2_{1,1}+\sigma^2_{2,1}}}\right)$

P1var12 <- sqrt(P1var+P1var1)/P1var # First term in the expressions of $g(L_1)$ and $g(W_1)$ in equation (3) in main paper $\left(\frac{\sigma^2_{1,1}+\sigma^2_{2,1}}{\sigma^2_{1,1}}\right)$.

P2var1 <- 1/(P2informationAll - P2information) # Variance of Partition 2 increment estimate ($\sigma^2_{2,2} = 1/(V_{N_2} - V_{1,2})$).

P2var11 <- P2var1 / sqrt(P2var+P2var1) # Partition 2 second term in equation (3) in main paper $\left(\frac{\sigma^2_{2,2}}{\sqrt{\sigma^2_{1,2}+\sigma^2_{2,2}}}\right)$.

P2var12 <- sqrt(P2var+P2var1)/P2var # First term in the expressions of $g(L_2)$ and $g(W_2)$ in equation (3) in main paper $\left(\frac{\sigma^2_{1,2}+\sigma^2_{2,2}}{\sigma^2_{1,2}}\right)$.

UMVCUE with the adaptive enrichment design

Partition 1

L1 <- -Inf # That is, $l_1 = -\infty$

gL1 <- P1var12 * (P1naive.estimate - L1) # $g(L_1) = \left(\frac{\sigma^2_{1,1}+\sigma^2_{2,1}}{\sigma^2_{1,1}}\right)(\hat{\theta}_{N_1} - l_1)$ in expression (3) in the main paper

w1 <- (b - ((P2prev*P2estimate) + (P3prev*P3estimate)))/P1prev # That is expression for w_1.

gw1 <- P1var12 * (P1naive.estimate - w1) # $g(W_1) = \left(\frac{\sigma^2_{1,1}+\sigma^2_{2,1}}{\sigma^2_{1,1}}\right)(\hat{\theta}_{N_1} - w_1)$ in expression (3) in the main paper

P1adj <- (dnorm(gL1)-dnorm(gw1))/(pnorm(gL1)-pnorm(gw1))

P1umvcue <- P1naive.estimate - (P1var11 * P1adj)

round(P1umvcue, 3)

-0.737

Partition 2

L2 <- -Inf # That is, $l_2 = -\infty$

gL2 <- P2var12 * (P2naive.estimate - L2) # $g(L_2)$ in expression (3) in the main paper

w2 <- (b - ((P1prev*P1estimate) + (P3prev*P3estimate)))/P2prev

gw2 <- P2var12 * (P2naive.estimate - w2) # $g(W_2)$ in expression (3) in the main paper

P2adj <- (dnorm(gL2)-dnorm(gw2))/(pnorm(gL2)-pnorm(gw2))

P2umvcue <- P2naive.estimate - (P2var11 * P2adj)

round(P2umvcue, 3)

-0.359

UMVCUE with selecting all partitions with stage 1 log hazard ratio ≥ 0

Partition 1

L1 <- -Inf

gL1 <- P1var12 * (P1naive.estimate - L1) # $g(L_1)$ in expression (3) in the main paper

w1 <- b # That is the value for w_1.

gw1 <- P1var12 * (P1naive.estimate - w1) # $g(W_1)$ in expression (3) in the main paper

P1adj <- (dnorm(gL1)-dnorm(gw1))/(pnorm(gL1)-pnorm(gw1))

P1umvcue <- P1naive.estimate - (P1var11 * P1adj)

round(P1umvcue, 3)

-0.631

Partition 2

L2 <- -Inf

gL2 <- P2var12 * (P2naive.estimate - L2) # $g(V_2)$ in expression (3) in the main paper

w2 <- b

gw2 <- P2var12 * (P2naive.estimate - w2) # $g(W_2)$ in expression (3) in the main paper

P2adj <- (dnorm(gL2)-dnorm(gw2))/(pnorm(gL2)-pnorm(gw2))

P2umvcue <- P2naive.estimate - (P2var11 * P2adj)

round(P2umvcue, 3)

-0.335

5.2.7 Duality confidence intervals

(i) Specifying the weights of stage 1 and 2 patients

w1 <- sqrt(0.75) # Stage 1 weight (w_1) 0.75 is approximately 90/120

w2 <- sqrt(0.25) # Stage 2 weight (w_2)

(ii) Computing estimates for stage 1 patients required to compute duality confidence intervals

TALLdata <- WinterEtAllData # Creating a copy of the dataset

TALLdata$SurvTime <- with(TALLdata, Surv(SurvTime2, Status2)) # Specifying survival time and status variables

TALLdata <- TALLdata[TALLdata$Stage==1,] # Selecting Stage 1 patients

Partition 1

TALLdata1 <- TALLdata[TALLdata$Partition==1,] # Selecting Partition 1 patients

a <- survdiff(SurvTime ~ TwoArm, data=TALLdata1, rho = 0) # Log rank test

Indic1 <- ((a$exp[2]-a$obs[2])>0) # Indicator that the expected deaths are more than observed deaths

Indic2 <- ((a$exp[2]-a$obs[2])<=0) # Indicator that the expected deaths are less or equal to the observed deaths

P1infoDualStg1 <- a$var[2,2] # Partition 1 Fisher information $\tilde{V}_{1,1}$ ($\tilde{\sigma}_{1,1} = 1/\tilde{V}_{1,1}$)

round(P1infoDualStg1, 3)

7.972 ## $\tilde{\sigma}_{1,1} = 1/\tilde{V}_{1,1} = 1/7.972 = 0.125$

P1.dualStg1 <- Indic1*-1*sqrt(a$chisq / P1infoDualStg1) + Indic2*sqrt(a$chisq / P1infoDualStg1) # That is $\tilde{\theta}_{1,1}$ (See notation in Figure S2(a)).

round(P1.dualStg1, 3)

-0.955

Partition 2

TALLdata2 <- TALLdata[TALLdata$Partition==2,] # Selecting Partition 2 patients

a <- survdiff(SurvTime ~ TwoArm, data=TALLdata2, rho = 0) # Log rank test

Indic1 <- ((a$exp[2]-a$obs[2])>0) # Indicator that the expected deaths are more than observed deaths

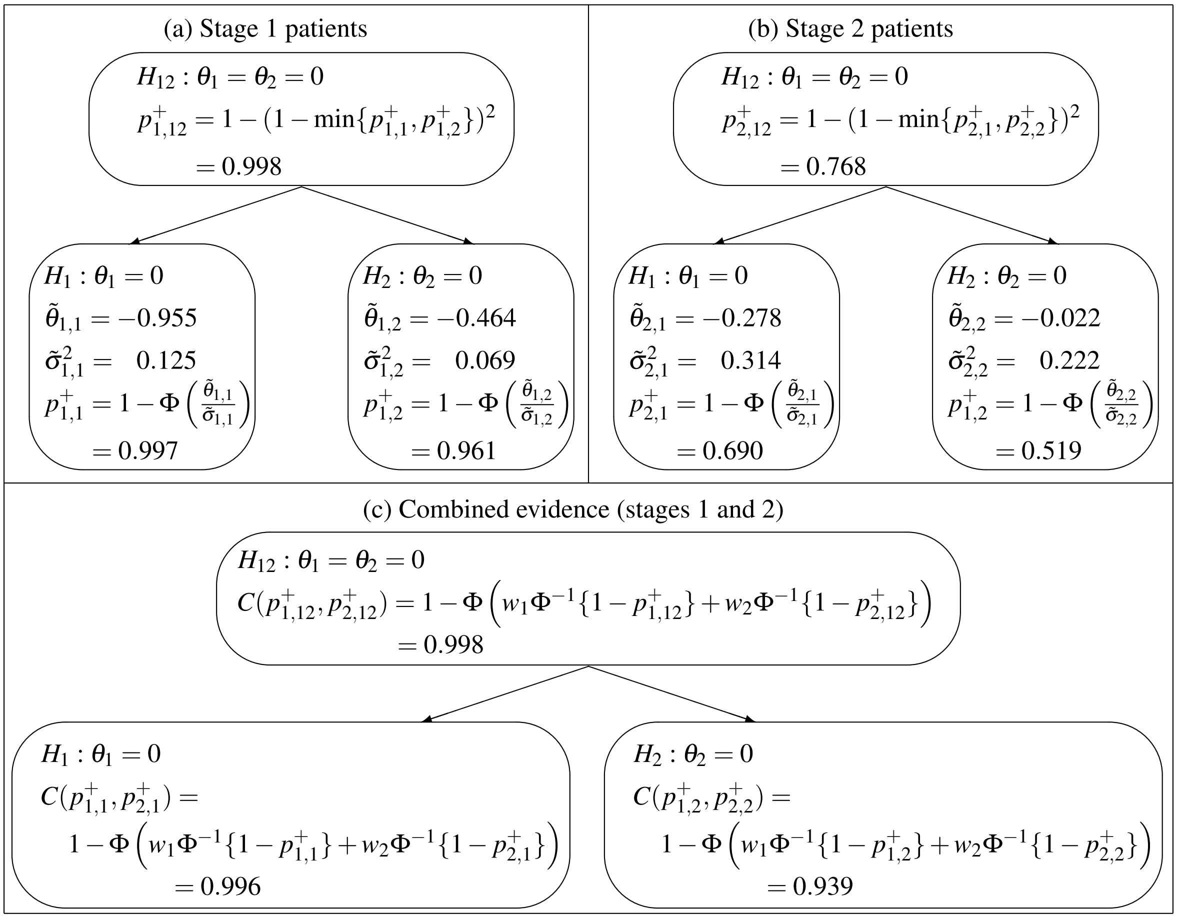

Figure S2: Hypothesis testing based on closure principle and combination of p-values. The weights $w_1 = \sqrt{0.75}$ and $w_2 = \sqrt{0.25}$. The alternative hypothesis is that the log hazard ratios are greater than 0. These are required for determining the lower limits of the simultaneous duality confidence intervals.

Indic2 <- ((a$exp[2]-a$obs[2])<=0) # Indicator that the expected deaths are less or equal to the observed deaths

P2infoDualStg1 <- a$var[2,2] # Partition 2 Fisher information $\tilde{V}_{1,2}$ ($\tilde{\sigma}_{1,2} = 1/\tilde{V}_{1,2}$)

round(P2infoDualStg1, 3)

14.484 *##* $\tilde{\sigma}_{1,2} = 1/\tilde{V}_{1,2} = 1/14.484 = 0.069$

P2.dualStg1 <- Indic1*-1*sqrt(a$chisq / P2infoDualStg1) + Indic2*sqrt(a$chisq / P2infoDualStg1) # That is $\tilde{\theta}_{1,2}$ (See notation in Figure S2(a)).

round(P2.dualStg1, 3)

-0.464

(iii) Computing estimates for stage 2 patients required to compute duality confidence intervals

TALLdata <- WinterEtAllData # Creating a copy of the dataset

TALLdata$SurvTime <- with(TALLdata, Surv(SurvTime2, Status2)) # Specifying survival time and status variables

TALLdata <- TALLdata[TALLdata$Stage==2,] # Selecting Stage 2 patients

Partition 1

TALLdata1 <- TALLdata[TALLdata$Partition==1,] # Selecting Partition 1 patients

a <- survdiff(SurvTime ~ TwoArm, data=TALLdata1, rho = 0) # Log rank test

Indic1 <- ((a$exp[2]-a$obs[2])>0) # Indicator that the expected deaths are more than observed deaths

Indic2 <- ((a$exp[2]-a$obs[2])<=0) # Indicator that the expected deaths are less or equal to the observed deaths

P1infoDualStg2 <- a$var[2,2] # Partition 1 Fisher information $\tilde{V}_{2,1}$ ($\tilde{\sigma}_{2,1} = 1/\tilde{V}_{2,1}$)

round(P1infoDualStg2, 3)

3.183 ## $\tilde{\sigma}_{2,1} = 1/\tilde{V}_{2,1} = 1/3.183 = 0.314$

P1.dualStg2 <- Indic1*-1*sqrt(a$chisq / P1infoDualStg2) + Indic2*sqrt(a$chisq / P1infoDualStg2) # # That is $\tilde{\theta}_{2,1}$ (See notation in Figure S2(b)).

round(P1.dualStg2, 3)

-0.278

Partition 2

TALLdata2 <- TALLdata[TALLdata$Partition==2,] # Selecting Partition 2 patients

a <- survdiff(SurvTime ~ TwoArm, data=TALLdata2, rho = 0) # Log rank test

Indic1 <- ((a$exp[2]-a$obs[2])>0) # Indicator that the expected deaths are more than observed deaths

Indic2 <- ((a$exp[2]-a$obs[2])<=0) # Indicator that the expected deaths are less or equal to the observed deaths

P2infoDualStg2 <- a$var[2,2] # Partition 2 Fisher information $\tilde{V}_{2,2}$ ($\tilde{\sigma}_{2,2} = 1/\tilde{V}_{2,2}$)

round(P2infoDualStg2, 3)

4.499 ## $\tilde{\sigma}_{2,2} = 1/\tilde{V}_{2,2} = 1/4.499 = 0.222$

P2.dualStg2 <- Indic1*-1*sqrt(a$chisq / P2infoDualStg2) + Indic2*sqrt(a$chisq / P2infoDualStg2) # That is $\tilde{\theta}_{2,2}$ (See notation in Figure S2(b)).

round(P2.dualStg2, 3)

-0.022

(iv) Hypothesis testing associated with lower bound (See Figure S2)

Stage 1 p-values

iPvalue1h1 <- 1 - pnorm(P1.dualStg1/sqrt(1/P1infoDualStg1)) # ($p^{+}_{1,1}$)

round(iPvalue1h1, 3)

0.997

iPvalue1h2 <- 1 - pnorm(P2.dualStg1/sqrt(1/P2infoDualStg1)) # ($p^{+}_{1,2}$)

round(iPvalue1h2, 3)

0.961

iPvalue1h12 <- 1 - (1 - min(c(iPvalue1h1, iPvalue1h2)))^2 # ($p^{+}_{1,12}$)

round(iPvalue1h12, 3)

0.998

Stage 2 p-values

iPvalue2h1 <- 1 - pnorm(P1.dualStg2/sqrt(1/P1infoDualStg2)) # ($p^{+}_{2,1}$)

round(iPvalue2h1, 3)

0.690

iPvalue2h2 <- 1 - pnorm(P2.dualStg2/sqrt(1/P2infoDualStg2)) # ($p^{+}_{2,2}$)

round(iPvalue2h2, 3)

0.519

iPvalue2h12 <- 1 - (1 - min(c(iPvalue2h1, iPvalue2h2)))^2 # ($p^{+}_{1,12}$)

round(iPvalue2h12, 3)

0.768

Combined p values

iPvalueOh1 <- 1 - pnorm((w1*qnorm(1-iPvalue1h1))+(w2*qnorm(1-iPvalue2h1))) # ($C(p^{+}_{1,1}, p^{+}_{2,1})$)

round(iPvalueOh1, 3)

0.996

iPvalueOh2 <- 1 - pnorm((w1*qnorm(1-iPvalue1h2))+(w2*qnorm(1-iPvalue2h2))) # ($C(p^+_{1,2}, p^+_{2,2})$).

round(iPvalueOh2, 3)

0.939

iPvalueOh12 <- 1 - pnorm((w1*qnorm(1-iPvalue1h12))+(w2*qnorm(1-iPvalue2h12))) # ($C(p^+_{1,12}, p^+_{2,12})$).

round(iPvalueOh12, 3)

0.998

Adjusted (for multiple hypotheses and interim analysis) p-values for determining whether log hazard ratios in partitions are greater than 0

P1.infPvalue <- max(iPvalueOh1, iPvalueOh12) # Adjusted p-value for the whether log hazard ratio in partition 1 is greater than 0

P1.infPvalue

0.998 ## This adjusted p-value is >0.025 and so we do not reject that the log hazard ratio in partition 1 is 0

P2.infPvalue <- max(iPvalueOh2, iPvalueOh12) # Adjusted p-value for the whether log hazard ratio in partition 2 is greater than 0

P2.infPvalue

0.998 ## This adjusted p-value is >0.025 and so we do not reject that the log hazard ratio in partition 2 is 0

(v) Obtaining the lower bounds

##
#################### In both partitions log hazards are concluded not greater than 0 ####################
####### Therefore expression (5) in the main paper is used to obtain the lower bounds in both partitions #########
##

R function for expression (5)

rootfunction.multiH <- function(stage1pvalues, ## Stage 1 pairwise p-values for partitions for which we are not computing the lower bound

stg1estimates, ## Stage 1 point estimate for the partition lower bound being computed

stage2pvalues, ## Stage 2 pairwise p-values for the partitions for which we are not computing the lower bound

stg2estimates, ## Stage 2 point estimate for the partition lower bound is being computed

v, ## The root been solved v in the expression for $\theta^I_{j,L}$ in expression (5) in the main paper

nhypo, ## The number of hypotheses in the intersection hypothesis in stage 1, that is $|I|$

stg2nhypo, ## The number of hypotheses in the intersection hypothesis in stage 2, that is $II \cap \mathcal{S}|$

wt1, ## Stage 1 weight (ω_1)

wt2, ## Stage 2 weight (ω_2)

alpha){ ## Type I error rate 0.025 if want two sided 95% confidence intervals

stg1.gpvalue <- 1 - pnorm((stg1estimates[1]-v)/sqrt(stg1estimates[2])) ## $p^+_{1,j}(v, \mathbf{x}_1)$ (Stage 1 generalised pairwise p-value for partition computing ## lower bound)

stg1.gpvalue <- 1 - (1 - min(c(stage1pvalues, stg1.gpvalue)))^nhypo ## $p^+_{1,I}(j, v)$

stg2.gpvalue <- 1 - pnorm((stg2estimates[1]-v)/sqrt(stg2estimates[2])) ## $p^+_{2,j}(v, \mathbf{x}_2)$ (Stage 2 generalised pairwise p-value for partition computing ## lower bound)

stg2.gpvalue <- 1 - (1 - min(c(stage2pvalues, stg2.gpvalue)))^stg2nhypo ## $p^+_{2,I}(j, v)$

combn.gpvalue <- 1 - pnorm((wt1*qnorm(1-stg1.gpvalue))+(wt2*qnorm(1-stg2.gpvalue))) ## $C(p^+_{1,I}(j,v), p^+_{2,I}(j,v))$

alpha - combn.gpvalue

}

Partition 1 lower bound

P1.lowerH1 <- (uniroot(rootfunction.multiH, ## Specifying seeking root based on the function above and for $I = \{1\}$ (H_1).
c(log(0.2), log(5)), ## Limits for the root
stage1pvalues=0.9999999999999, ## Since the intersection hypothesis is pairwise, we set this close 1
stage2pvalues=0.9999999999999, ## Since the intersection hypothesis is pairwise, we set this close 1
stg1estimates=c(P1.dualStg1, 1/P1infoDualStg1), ## ($\tilde{\theta}_{1,1}$, $\tilde{\sigma}_{1,1} = 1/\tilde{V}_{1,1}$) $= (-0.955, 0.125)$ See Figure S2 and 5.2.7(ii) above
stg2estimates=c(P1.dualStg2, 1/P1infoDualStg2), ## ($\tilde{\theta}_{2,1}$, $\tilde{\sigma}_{2,1} = 1/\tilde{V}_{2,1}$) $= (-0.278, 0.314)$ See Figure S2 and 5.2.7(iii) above
nhypo=1, ## Number of hypotheses is 1 since intersection hypothesis consists of one partition

stg2nhypo=1, ## Number of hypotheses is 1 since intersection hypothesis consists of one partition
wt1=w1,
wt2=w2,
alpha=0.025)
)$root

round(P1.LowerH1, 3)   ## This is $\theta_{1,L}^{\{1\}}$ in expression (5) in the main paper

-1.361

P1.LowerH12 <- (uniroot(rootfunction.multiH,   ## Specifying seeking root based on the function above and for $I = \{1,2\}$ (H_{12}).
c(log(0.2), log(5)),
stage1pvalues=iPvalue1h2, ## ($p_{1,2}^{+} = 0.961$); Stage 1 Partition 2 pairwise p-value; See Figure S2 and 5.2.7(iv) above
stage2pvalues=iPvalue2h2, ## ($p_{2,2}^{+} = 0.519$); Stage 2 Partition 2 pairwise p-value; See Figure S2 and 5.2.7(iv) above
stg1estimates=c(P1.dualStg1, 1/P1infoDualStg1),
stg2estimates=c(P1.dualStg2, 1/P1infoDualStg2),
nhypo=2, ## Number of hypotheses is 2 since intersection hypothesis H_{12} consists of two partitions
stg2nhypo=2, ## Number of hypotheses is 2 since both partitions continue to stage 2 so that H_{12} is tested using data from two partitions
wt1=w1,
wt2=w2,
alpha=0.025)
)$root

round(P1.LowerH12, 3)   ## This is $\theta_{1,L}^{\{1,2\}}$ in expression (5) in the main paper

-1.499

P1.lowerbound <- min(c(P1.LowerH1, P1.LowerH12))   ## Partition 1 lower bound. As per expression (5) in main paper, it is $\min\{\theta_{1,L}^{\{1\}}, \theta_{1,L}^{\{1,2\}}\}$.
round(P1.lowerbound, 3)

-1.499

<u>Partition 2 lower bound</u>

P2.LowerH2 <- (uniroot(rootfunction.multiH,
c(log(0.2), log(5)),
stage1pvalues=0.9999999999999, ## Since the intersection hypothesis is pairwise, we set this close 1
stage2pvalues=0.9999999999999, ## Since the intersection hypothesis is pairwise, we set this close 1
stg1estimates=c(P2.dualStg1, 1/P2infoDualStg1), ## ($\tilde{\theta}_{1,2}$, $\tilde{\sigma}_{1,2} = 1/\tilde{V}_{1,2}$) = $(-0.464, 0.069)$ See Figure S2 and 5.2.7(ii) above
stg2estimates=c(P2.dualStg2, 1/P2infoDualStg2), ## ($\tilde{\theta}_{2,2}$, $\tilde{\sigma}_{2,2} = 1/\tilde{V}_{2,2}$) = $(-0.022, 0.222)$ See Figure S2 and 5.2.7(iii) above
nhypo=1, ## Number of hypotheses is 1 since intersection hypothesis consists of one partition
stg2nhypo=1, ## Number of hypotheses is 1 since intersection hypothesis consists of one partition
wt1=w1,
wt2=w2,
alpha=0.025)
)$root

round(P2.LowerH2, 3)   ## This is $\theta_{2,L}^{\{1\}}$ in expression (5) in the main paper

-0.806

P2.LowerH12 <- (uniroot(rootfunction.multiH,
c(log(0.2), log(5)),
stage1pvalues=iPvalue1h1, ## ($p_{1,1}^{+} = 0.997$); Stage 1 Partition 1 pairwise p-value; See Figure S2 and 5.2.7(iv) above
stage2pvalues=iPvalue2h1, ## ($p_{2,1}^{+} = 0.690$); Stage 2 Partition 1 pairwise p-value; See Figure S2 and 5.2.7(iv) above
stg1estimates=c(P2.dualStg1, 1/P2infoDualStg1),
stg2estimates=c(P2.dualStg2, 1/P2infoDualStg2),
nhypo=2, ## Number of hypotheses is 2 since intersection hypothesis H_{12} consists of two partitions
stg2nhypo=2, ## Number of hypotheses is 2 since both partitions continue to stage 2 so that H_{12} is tested using data from two partitions
wt1=w1,
wt2=w2,

alpha=0.025)
)$root

round(P2.LowerH12, 3)   ## This is $\theta_{2,L}^{\{1,2\}}$ in expression (5) in the main paper

-0.911

P2.lowerbound <- min(c(P2.LowerH2, P2.LowerH12))   ## Partition 2 lower bound. As per expression (5) in main paper, it is $\min\{\theta_{2,L}^{\{2\}}, \theta_{2,L}^{\{1,2\}}\}$.

round(P2.lowerbound, 3)

-0.911

(vi) Hypothesis testing associated with upper bound (See Figure S3)

Stage 1 p-values

sPvalue1h1 <- 1 - pnorm(-P1.dualStg1/sqrt(1/P1infoDualStg1)) # ($p_{1,1}^{-}$)

round(sPvalue1h1, 3)

0.003

sPvalue1h2 <- 1 - pnorm(-P2.dualStg1/sqrt(1/P2infoDualStg1)) # ($p_{1,2}^{-}$)

round(sPvalue1h2, 3)

0.039

sPvalue1h12 <- 1 - (1 - min(c(sPvalue1h1, sPvalue1h2)))^2 # ($p_{1,12}^{-}$)

round(sPvalue1h12, 3)

0.006

Stage 2 p-values

sPvalue2h1 <- 1 - pnorm(-P1.dualStg2/sqrt(1/P1infoDualStg2)) # ($p_{2,1}^{-}$)

round(sPvalue2h1, 3)

0.310

sPvalue2h2 <- 1 - pnorm(-P2.dualStg2/sqrt(1/P2infoDualStg2)) # ($p_{2,2}^{-}$)

round(sPvalue2h2, 3)

0.481

sPvalue2h12 <- 1 - (1 - min(c(sPvalue2h1, sPvalue2h2)))^2 # ($p_{2,12}^{-}$)

round(sPvalue2h12, 3)

0.524

Combined p values

sPvalueOh1 <- 1 - pnorm((w1*qnorm(1-sPvalue1h1))+(w2*qnorm(1-sPvalue2h1))) # ($C(p_{1,1}^{-}, p_{2,1}^{-})$)

round(sPvalueOh1, 3)

0.004

sPvalueOh2 <- 1 - pnorm((w1*qnorm(1-sPvalue1h2))+(w2*qnorm(1-sPvalue2h2))) # ($C(p_{1,2}^{-}, p_{2,2}^{-})$)

round(sPvalueOh2, 3)

0.061

sPvalueOh12 <- 1 - pnorm((w1*qnorm(1-sPvalue1h12))+(w2*qnorm(1-sPvalue2h12))) # ($C(p_{1,12}^{-}, p_{2,12}^{-})$)

round(sPvalueOh12, 3)

0.016

Adjusted (for multiple hypotheses and interim analysis) p-values for determining whether log hazard ratios in partitions are less than 0

P1.supPvalue <- max(sPvalueOh1, sPvalueOh12) # Adjusted p-value for whether log hazard ratio in partition 1 is less than 0

P1.supPvalue

0.016   ## This adjusted p-value is <0.025 and so we conclude that the negative of log hazard ratio in partition 1 is greater than 0

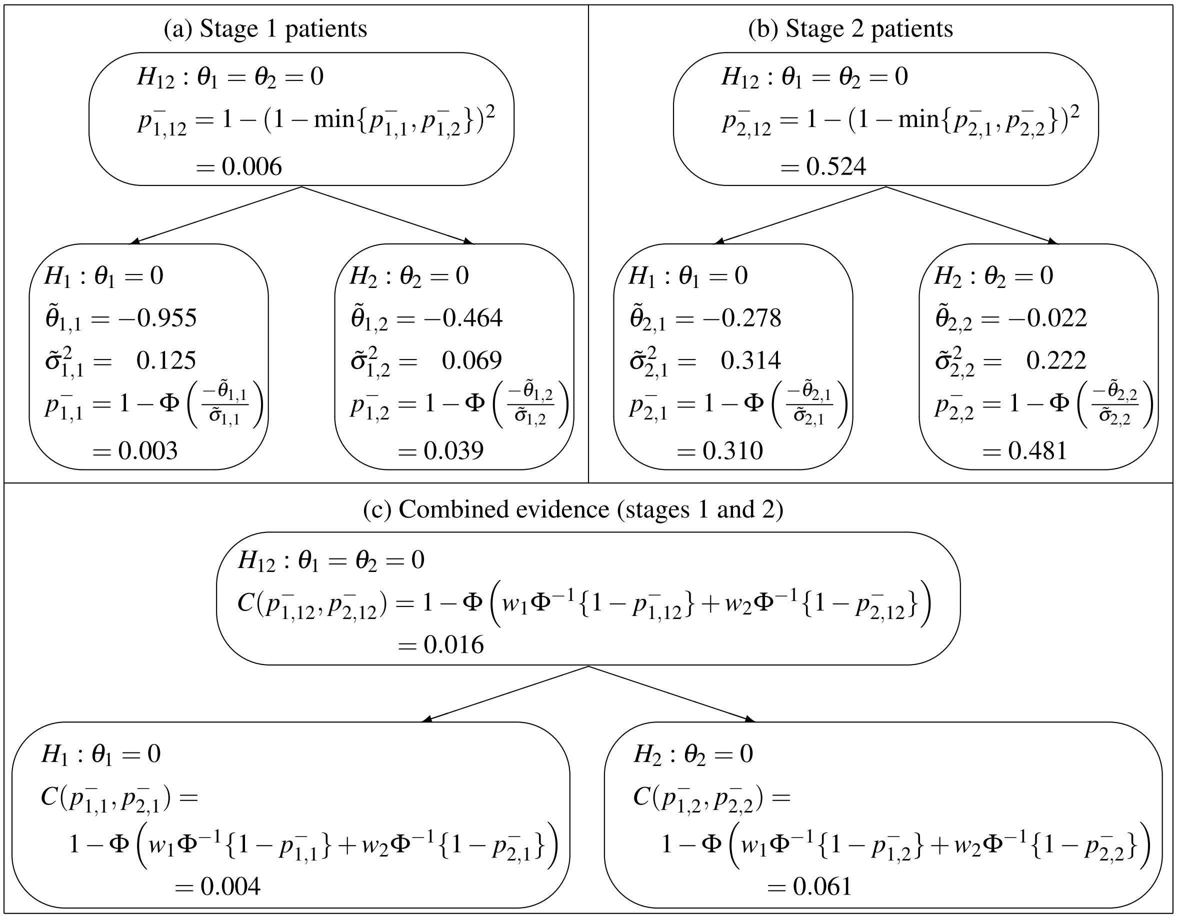

Figure S3: Hypothesis testing based on closure principle and combination of p-values. The weights $w_1 = \sqrt{0.75}$ and $w_2 = \sqrt{0.25}$. The alternative hypothesis is that the log hazard ratios are less than 0. These are required for determining the upper limits of the simultaneous duality confidence intervals.

P2.supPvalue <- max(sPvalueOh2, sPvalueOh12) # Adjusted p-value for whether log hazard ratio in partition 2 is greater than 0

P2.supPvalue

0.061 ## This adjusted p-value is >0.025 and so we do not reject that the negative of log hazard ratio in partition 2 is 0

(vii) Obtaining the upper bounds

```
##############################################################################################
##################### In partition 1 negative log hazard is concluded greater than 0 #####################
##################### In partition 2 negative log hazard is not concluded greater than 0 ###################
################## Therefore the lower bound for the negative log hazard in partition is 0 ##################
############ And expression (5) in the main paper is used to obtain the lower bound in Partition 2 ###########
##############################################################################################
```

Partition 1 upper bound

P1.upperbound <- 0, ## This is the upper bound for the log hazard ratio in partition 1

Partition 2 upper bound

We obtain lower bound for negative log hazard ratio. Upper bound for log hazard ratio is negative of this

```
P2.UpperH1 <- (uniroot(rootfunction.multiH,
    c(log(0.2), log(5)),
    stage1pvalues=0.9999999999999, ## Since the intersection hypothesis is pairwise, we set this close 1
```

```
stage2pvalues=0.9999999999999, ## Since the intersection hypothesis is pairwise, we set this close 1
stg1estimates=c(-P2.dualStg1, 1/P2infoDualStg1), ## (−θ̃1,2, σ̃1,2 = 1/Ṽ1,2) = (0.464,0.069) See Figures S2 & S3 and 5.2.7(ii) above
stg2estimates=c(-P2.dualStg2, 1/P2infoDualStg2), ## (−θ̃2,2, σ̃2,2 = 1/Ṽ2,2) = (−0.022,0.222) See Figure S2 & S3 and 5.2.7(iii) above
nhypo=1, ## Number of hypotheses is 1 since intersection hypothesis consists of one partition
stg2nhypo=1, ## Number of hypotheses is 1 since intersection hypothesis consists of one partition
wt1=w1,
wt2=w2,
alpha=0.025)
)$root

round(P2.UpperH12, 3)
```

-0.093

```
P2.UpperH12 <- Inf    ### That is θ_{2,L}^{{1,2}} is set to ∞ since hypothesis H12 is rejected.

P2.upperbound <- min (c(P2.UpperH1, P2.UpperH12))
P2.upperbound <-  -P2.upperbound    ## Upper bound is negative of the lower bound of the lower bound for the negative log hazard ratio
round(P2.upperbound, 3)
```

0.093

5.3 R code for computing estimates for data that includes a bootstrap sample

In this section, we describe the R code used to obtain the various estimates for the example data combined with a boostrap sample of the same size as the example data. The estimates are summarised in Table S2. The R Code to obtain the point estimates and the naive confidence intervals can be deduced from the R code in Section 5.2.

The estimates required to obtain the duality confidence intervals are summarised in Figure S4. The p-values required to compute the lower bounds are not presented but can be computed using estimates in the figure. Based on them, it can not be concluded that the log hazard ratios in each of Partitions 1 and 2 are greater than 0 and so the lower bounds are obtained using the same R code as 5.2.7 (v) above, which we do not repeat. We give the R code for obtaining the upper bounds below.

Adjusted (for multiple hypotheses and interim analysis) p-values for determining whether log hazard ratios in partitions are less than 0

```
P1.supPvalue <- max(0.001, 0.002) # Adjusted p-value for whether log hazard ratio in partition 1 is less than 0
P1.supPvalue
```

0.002 ## This adjusted p-value is <0.025 and so we conclude that the negative of log hazard ratio in partition 1 is greater than 0

```
P2.supPvalue <- max(0.005, 0.002) # Adjusted p-value for whether log hazard ratio in partition 2 is greater than 0
P2.supPvalue
```

0.005 ## This adjusted p-value is <0.025 and so we conclude that the negative of log hazard ratio in partition 2 is greater than 0

```
###################################################################################################################
### Since the adjusted p-values for testing effects in selected partition are both <0.025, expression (4) in the main paper is required to obatin the ###
####################################        upper bounds for the effects in the two partitions        ####################################
###################################################################################################################
```

R function for Expression (4) in the main paper

```
rootfunction <- function(pmplus,    ## This is p_M^+ in main paper expression (4). Maximum stage 1 p-value for hypotheses that exclude S
                  stg1estimates,    ## Stage 1 point estimate for the partition lower bound being computed
                  stg2estimates,    ## Stage 2 point estimate for the partition lower bound being computed
```

Table S2: Summary of the estimates from the constructed example that includes a bootstrap sample

	Stage 1[§]	All data[§]	Increment[§]	UMVCUE ($\hat{\theta}_{U_j}$)[§]		Confidence intervals	
	$\hat{\theta}_{1,j}$ ($\sigma^2_{1,j}$)	$\hat{\theta}_{N_j}$ ($\sigma^2_{N_j}$)	$\hat{\theta}_{2,j}$ ($\sigma^2_{2,j}$)	AT[†]	(IND)[‡]	Naive	Duality
Partition 1	-0.725 (0.101)	-0.647 (0.049)	-0.572 (0.096)	-0.638	-0.551	(-1.143, -0.150)	(-1.218, -0.123)
Partition 2	-0.392 (0.054)	-0.434 (0.027)	-0.476 (0.055)	-0.434	-0.431	(-0.804, -0.064)	(-0.831, -0.028)

[§] $j = 1$ for partition 1 and $j = 2$ for partition 2; [†] AT=Adaptive threshold design; [†] IND=Independently selecting partitions.

v, ## The root being solved
npol, ## Number of partition in stage 1, that is, K
nsel, ## Number of partitions selected to continue to stage 2, that is, $|\mathbb{S}|$
wt1, ## Stage 1 weight (ω_1)
wt2, ## Stage 2 weight (ω_2)
alpha){ ## Type I error rate 0.025 if want two sided 95% confidence intervals
stg1.gpvalue <- 1 - pnorm((stg1estimates[1]-v)/sqrt(stg1estimates[2])) ## $p^+_{1,j}(v, \mathbf{x}_1)$ (Stage 1 generalised pairwise p-value for partition computing ## lower bound)
stg1.gpvalue <- 1 - (1 - stg1.gpvalue)^npol ## $1-(1-p^+_{1,j}(v,\mathbf{x}_1))^K$
stg1.gpvalue <- max(c(pmplus, stg1.gpvalue)) ## $\max\{p^+_M,\ 1-(1-p^+_{1,j}(v,\mathbf{x}_1))^K\}$
stg2.gpvalue <- 1 - pnorm((stg2estimates[1]-v)/sqrt(stg2estimates[2])) ## $p^+_{2,j}(v, \mathbf{x}_2)$ (Stage 2 generalised pairwise p-value for partition computing ## lower bound)
stg2.gpvalue <- 1 - (1 - stg2.gpvalue)^nsel ## $1-(1-p^+_{2,j}(v,\mathbf{x}_2))^{|\mathbb{S}|}$
combn.gpvalue <- 1 - pnorm((wt1*qnorm(1-stg1.gpvalue))+(wt2*qnorm(1-stg2.gpvalue))) ## $\sup\{\cdot\}$ in Expression (4) in the main paper
alpha - combn.gpvalue
}

Partition 1 upper bound

P1.rootsolution <- (uniroot(rootfunction,
c(log(0.2), log(5)),
pmplus=0.000000000001, ## Both partitions are selected so we should set $p^+_M = 0$. Because of combination function we use a small +ve number
stg1estimates=c(0.831, 0.065), ## This is $(-\tilde{\theta}_{1,1}, \tilde{\sigma}^2_{1,1})$: See Figure S4
stg2estimates=c(0.192, 0.215), ## This is $(-\tilde{\theta}_{2,1}, \tilde{\sigma}^2_{2,1})$: See Figure S4
npol=2, ## That is the number of partitions in stage 1 $K = 2$.
nsel=2, ## That is the number of partitions selected to continues to stage 2 $|\mathbb{S}| = 2$.
wt1=sqrt(0.75), ## Stage 1 weight ω_1. We use same weights as before.
wt2=sqrt(0.25), ## Stage 2 weight ω_1. We use same weights as before.
alpha=0.025)
)$root

P1.UpperBound <- min(c(0, -P1.rootsolution))

round(P1.UpperBound, 3)

-0.123

Partition 2 upper bound

P2.rootsolution <- (uniroot(rootfunction,
c(log(0.2), log(5)),
pmplus=0.000000000001, ## Both partitions are selected so we should set $p^+_M = 0$. Because of combination function we use a small +ve number
stg1estimates=c(0.461, 0.036), ## This is $(-\tilde{\theta}_{1,2}, \tilde{\sigma}^2_{1,2})$: See Figure S4
stg2estimates=c(0.337, 0.115), ## This is $(-\tilde{\theta}_{2,2}, \tilde{\sigma}^2_{2,2})$: See Figure S4
npol=2,
nsel=2,
wt1=sqrt(0.75),
wt2=sqrt(0.25),
alpha=0.025)

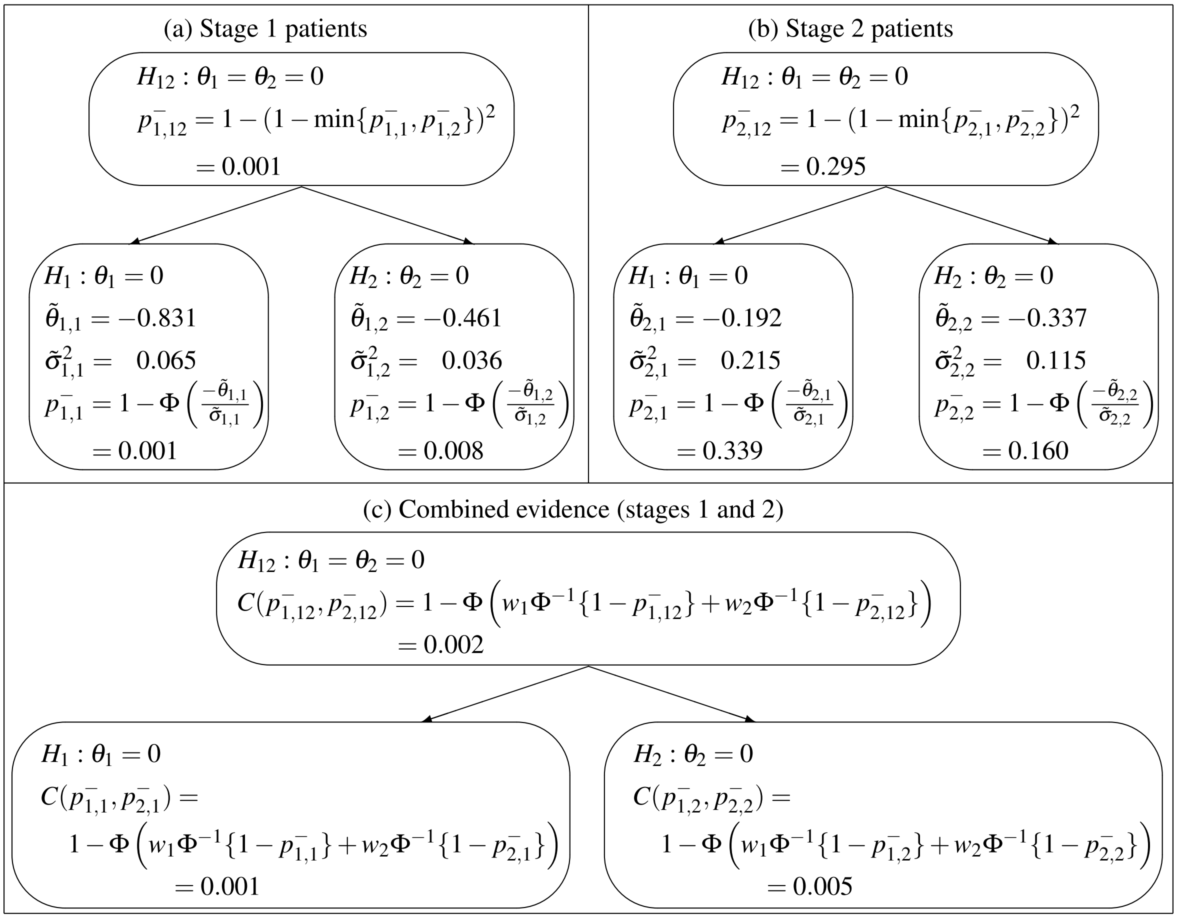

Figure S4: Hypothesis testing based on closure principle and combination of p-values. The weights $w_1 = \sqrt{0.75}$ and $w_2 = \sqrt{0.25}$. The alternative hypothesis is that the log hazard ratios are less than 0. These are required for determining the upper limits of the simultaneous duality confidence intervals.

```
    )$root

P2.UpperBound <- min(c(0, -P2.rootsolution))

round(P2.UpperBound, 3)
```

-0.028

6 All confidence intervals properties

Table S3: Coverage probability and type I error rate (Weibull distribution, $\gamma = 0.5$)

True log hazard ratios	Selected partitions ($\mathcal{S}$)	Partition	Coverage (Type I error)[†] Naive	Coverage (Type I error)[†] Duality
$\theta_1 = \theta_2 = \theta_3 = \theta_4 = 0.0198$	All	Global[‡]	94.8 (4.5)	98.3 (1.7)
		1	98.6 (1.2)	99.6 (0.4)
		2	98.7 (1.1)	99.6 (0.4)
		3	98.7 (1.1)	99.6 (0.4)
		4	98.6 (1.1)	99.6 (0.4)
	1, 2 & 3	Global[‡]	96.1 (3.0)	99.1 (0.9)
		1	98.7 (1.1)	99.7 (0.3)
		2	98.7 (1.1)	99.7 (0.3)
		3	98.7 (0.9)	99.7 (0.3)
	1 & 2	Global[‡]	96.0 (3.1)	99.2 (0.8)
		1	98.0 (1.6)	99.5 (0.5)
		2	98.0 (1.5)	99.7 (0.3)
	1	1	96.3 (2.9)	99.6 (0.4)
$\theta_1 = -0.2231$, $\theta_2 = -0.0953$, $\theta_3 = 0.3365$, $\theta_4 = 0.4055$	All	Global[‡]	93.0 (6.6)	98.4 (1.5)
		1	98.0 (1.9)	99.9 (0.0)
		2	98.2 (1.7)	99.9 (0.0)
		3	98.3 (1.6)	99.3 (0.7)
		4	98.4 (1.5)	99.3 (0.7)
	1, 2 & 3	Global[‡]	96.0 (3.1)	99.1 (0.5)
		1	98.7 (1.0)	99.9 (0.0)
		2	98.8 (0.9)	99.8 (0.0)
		3	98.5 (1.1)	99.5 (0.5)
	1 & 2	Global[‡]	96.3 (1.4)	99.2 (0.0)
		1	98.2 (0.7)	99.6 (0.0)
		2	98.1 (0.7)	99.6 (0.0)
	1	1	96.1 (1.5)	99.4 (0.0)
$\theta_1 = -0.4055$, $\theta_2 = -0.2231$, $\theta_3 = -0.0953$, $\theta_4 = 0$	All	Global[‡]	95.4 (2.6)	98.7 (0.7)
		1	98.9 (0.6)	99.8 (0.0)
		2	98.8 (0.6)	99.8 (0.0)
		3	98.8 (0.7)	99.8 (0.0)
		4	98.8 (0.7)	99.2 (0.7)
	1, 2 & 3	Global[‡]	93.3 (0.3)	97.6 (0.0)
		1	97.5 (0.1)	99.1 (0.0)
		2	97.9 (0.2)	99.3 (0.0)
		3	97.8 (0.0)	99.2 (0.0)
	1 & 2	Global[‡]	93.8 (0.7)	98.2 (0.0)
		1	96.9 (0.5)	99.1 (0.0)
		2	96.9 (0.2)	99.0 (0.0)
	1	1	94.0 (0.9)	98.6 (0.0)

7 Simulation results for additional scenarios with the adaptive threshold design

7.1 Summary of the main findings

Sections 7.2 to 7.6 describe in detail the findings for more scenarios (simulation results are given in Tables S4 to S17 and Figures S5 to S11), for the case of performing subpopulation selection using the adaptive threshold design selection rule described in the main paper. For all the scenarios, the probabilities assessing the properties of the naive and the duality confidence intervals (Tables S5, S7, S9, S11, S15 and S17) are almost equal to those given in Table 5 in the main paper and hence the same finding. The finding is that we consider the duality confidence regions to have at least the nominal coverage probabilities and the probabilities that at least one upper bound is less than the true value to be less than the target 2.5% but usually very small, which is partly explained by non-informative confidence intervals. Because of the very similar results for the characteristics of the confidence intervals, we do not describe the results of the confidence intervals further, focussing on the point estimates only in Sections 7.2 to 7.6.

For the point estimates, the summary is that we recommend having $\tilde{t}_1 > t_1$ and obtaining point estimates using the UMVCUE $\hat{\theta}_{U_j}$. Although the recommendation is the same for all scenarios, there are some differences, especially for the naive estimates, which we describe in detail in Sections 7.2 to 7.6.

7.2 Comparing the results for $\gamma = 0.5$, $\gamma = 1.0$ (exponenital distribution) and $\gamma = 1.5$

The results for the point estimates for $\gamma = 0.5$ are given in the main paper (Table 4 and Figure 4) while the results for $\gamma = 1$ (exponential distribution) and $\gamma = 1.5$ are given in this document; Table S4 and Figure S5 for $\gamma = 1.0$, and Table S6 and Figure S6 for $\gamma = 1.5$. The results (biases and RMSEs) for the three values of γ are almost identical. Therefore, as in the case of $\gamma = 0.5$, for $\gamma = 1$ and $\gamma = 1.5$, we recommend the UMVCUE $\hat{\theta}_{U_j}$ evaluated at $\tilde{t}_1 > t_1$.

7.3 Assessing the impact of a slower recruitment rate

To assess the impact of a slower patient recruitment so that the trial is longer, for $\gamma = 0.5$, we performed simulations where stages 1 and 2 are each approximately 2 years. In this case, the required sample is smaller and so we assume up to 1700 patients can be recuited uniformly over 4 years., with $\tilde{t}_1$ corresponding to 630 days after the interim analysis. The average additional follow-up events from stage 1 censored observations in the selected partitions was approximately 40. The selection probabilities (not reported) are similar to those in the main paper Table 2, which is the case of a quicker recruitment rate. The simulation results for point estimates are given in Table S8 and Figure S7. Generally, the results are similar to those in the main paper (Table 4 and Figure 4) and hence UMVCUE with $\tilde{t}_1 > t_1$ remain the best point estimator for a longer trial.

7.4 The impact of the number of events from stage 1 patients without events at the interim analysis

To assess the impact of the number of the number of events from stage 1 censored observations, for $\gamma = 1$ (Exponential distribution) and $\gamma = 1.5$, we peformed simulations with $\tilde{t}_1$ corresponding to 250 days and 150 days, respectively, after the interim analysis. The average number of additional deaths in the selected partitions was approximately 80, compared to approximately 40 in the other scenarios. The properties of the point estimators are summarised in Tables S10 and S12, and Figures S8 and S9. As expected, the naive estimator with $\tilde{t}_1 > t_1$ $(\hat{\theta}_{N_j})$ has smaller biases and RMSEs for the cases with more events from stage 1 patients without events at the interim analysis (Tables S10 and S12, and Figure S8 and S9) than the case of fewer events for stage 1 patients without events at the interim analysis (Table S4 and Figure S5 for $\gamma = 1.0$, and Table S6 and Figure S6 for $\gamma = 1.5$). Also, the UMVCUE $(\hat{\theta}_{U_j})$ with $\tilde{t}_1 > t_1$ exhibits less bias and RMSE for the former and consequently much better properties than the UMVCUE $(\hat{\theta}_{U_j})$ with $\tilde{t}_1 = t_1$ when there are more events from stage 1 patients without events at the interim analysis. Hence, we recommend the UMVCUE $\hat{\theta}_{U_j}$ evaluated at $\tilde{t}_1 > t_1$.

7.5 Trials with fewer events

To assess the impact of the total number of events in a trial, for $\gamma = 0.5$, we performed simulations for the case of 200 events in each of stages 1 and 2. We used the same recruitment rate as the scenario considered for the results of Table 4 and Figure 4 in the main paper. The average number of the extra follow-up events for censored stage 1 observations in the selected partitions is 30. The biases of the naive point estimators $\hat{\theta}_{N_j}$ (Table S14 and Figure S10) tend to be higher than the comparable results in the main paper (Tables 4 and Figure 4). This is attributable to the higher variability of stage 1 estimates and hence lower probabilities of making the ideal decisions as well as the smaller size of the unbiased stage 2 data. However, the UMVCUE $\hat{\theta}_{U_j}$ for $\tilde{t}_1 > t_1$ is still the preferred point estimator.

7.6 Performing subpopulation selection earlier in the trial

To assess the impact of conducting subpopulation selection earlier in the trial, we performed simulations with the interim analysis done after 200 events and the trial stops after 400 events from patients recruited in stage 2 (in most of the previous results it is 300 events in each stage), with $\tilde{t}_1$ corresponding to 320 days after the interim analysis. The average number of events from stage 1 patients without events at the interim analysis in the selected partitions was approximately 40. The results of the point estimators are given in Table S16 and Figure S11. Compared to the case of 300 events in each stage (Table 4 and Figure 4 in the main paper), the biases of the naive estimtors are smaller which is attributable to more weight for the unbiased stage 2 data. As in the other scenarios, having $\tilde{t}_1 > t_1$ and using the UMVCUE $\hat{\theta}_{U_j}$ provides the best point estimates.

8 Exponential distribution simulation results

Table S4: Simulated biases and mean squared errors of the estimators for the log hazard ratios (Exponential distribution)

Selected		Simulated bias				Root mean squared error			
		$\hat{\theta}_{N_j}$		$\hat{\theta}_{U_j}$		$\hat{\theta}_{N_j}$		$\hat{\theta}_{U_j}$	
partitions ($\mathcal{S}$)	Partition	$\tilde{t}_1 > t_1$	$\tilde{t}_1 = t_1$	$\tilde{t}_1 > t_1$	$\tilde{t}_1 = t_1$	$\tilde{t}_1 > t_1$	$\tilde{t}_1 = t_1$	$\tilde{t}_1 > t_1$	$\tilde{t}_1 = t_1$
All	1	0.0404	0.0518	0.0128	-0.0002	0.1463	0.1660	0.1510	0.1858
	2	0.0411	0.0529	0.0132	0.0000	0.1457	0.1655	0.1507	0.1862
	3	0.0408	0.0523	0.0128	-0.0005	0.1458	0.1654	0.1505	0.1859
	4	0.0416	0.0533	0.0140	0.0012	0.1466	0.1666	0.1511	0.1863
1, 2 & 3	1	0.0240	0.0298	0.0095	0.0034	0.1301	0.1424	0.1476	0.1901
	2	0.0200	0.0253	0.0054	-0.0016	0.1294	0.1425	0.1468	0.1907
	3	0.0209	0.0263	0.0062	-0.0005	0.1302	0.1434	0.1469	0.1911
1 & 2	1	0.0207	0.0250	0.0040	-0.0022	0.1174	0.1258	0.1330	0.1589
	2	0.0233	0.0277	0.0081	0.0032	0.1173	0.1274	0.1330	0.1603
1	1	0.0221	0.0250	0.0040	0.0007	0.0929	0.0976	0.1043	0.1145
True log hazard ratios: $\theta_1 = -0.2231$, $\theta_2 = -0.0953$, $\theta_3 = 0.3365$, $\theta_4 = 0.4055$									
All	1	0.0732	0.0942	0.0244	-0.0004	0.1643	0.1897	0.1630	0.2079
	2	0.0711	0.0903	0.0246	0.0004	0.1621	0.1856	0.1605	0.2015
	3	0.0587	0.0755	0.0194	0.0000	0.1447	0.1659	0.1450	0.1827
	4	0.0603	0.0764	0.0222	0.0030	0.1438	0.1651	0.1428	0.1798
1, 2 & 3	1	0.0255	0.0318	0.0066	-0.0024	0.1395	0.1541	0.1555	0.1962
	2	0.0270	0.0333	0.0084	0.0000	0.1354	0.1494	0.1502	0.1884
	3	0.0223	0.0274	0.0075	0.0010	0.1260	0.1382	0.1387	0.1705
1 & 2	1	-0.0111	-0.0136	-0.0036	-0.0019	0.1205	0.1296	0.1348	0.1570
	2	-0.0105	-0.0123	-0.0030	-0.0005	0.1177	0.1271	0.1314	0.1535
1	1	-0.0125	-0.0141	-0.0010	0.0010	0.0952	0.0990	0.1068	0.1154
True log hazard ratios: $\theta_1 = -0.4055$, $\theta_2 = -0.2231$, $\theta_3 = -0.0953$, $\theta_4 = 0$									
All	1	0.0047	0.0058	0.0001	-0.0023	0.1494	0.1673	0.1524	0.1743
	2	0.0057	0.0074	0.0015	0.0002	0.1449	0.1624	0.1474	0.1683
	3	0.0050	0.0064	0.0010	-0.0006	0.1420	0.1592	0.1444	0.1650
	4	0.0055	0.0073	0.0017	0.0007	0.1396	0.1567	0.1419	0.1622
1, 2 & 3	1	-0.0659	-0.0831	-0.0227	-0.0048	0.1494	0.1674	0.1558	0.1983
	2	-0.0593	-0.0721	-0.0199	0.0011	0.1456	0.1633	0.1546	0.1968
	3	-0.0571	-0.0697	-0.0183	0.0019	0.1380	0.1532	0.1446	0.1828
1 & 2	1	-0.0677	-0.0790	-0.0202	-0.0026	0.1384	0.1527	0.1420	0.1694
	2	-0.0563	-0.0642	-0.0118	0.0083	0.1263	0.1363	0.1310	0.1554
1	1	-0.0498	-0.0562	-0.0131	-0.0080	0.1042	0.1099	0.1057	0.1132

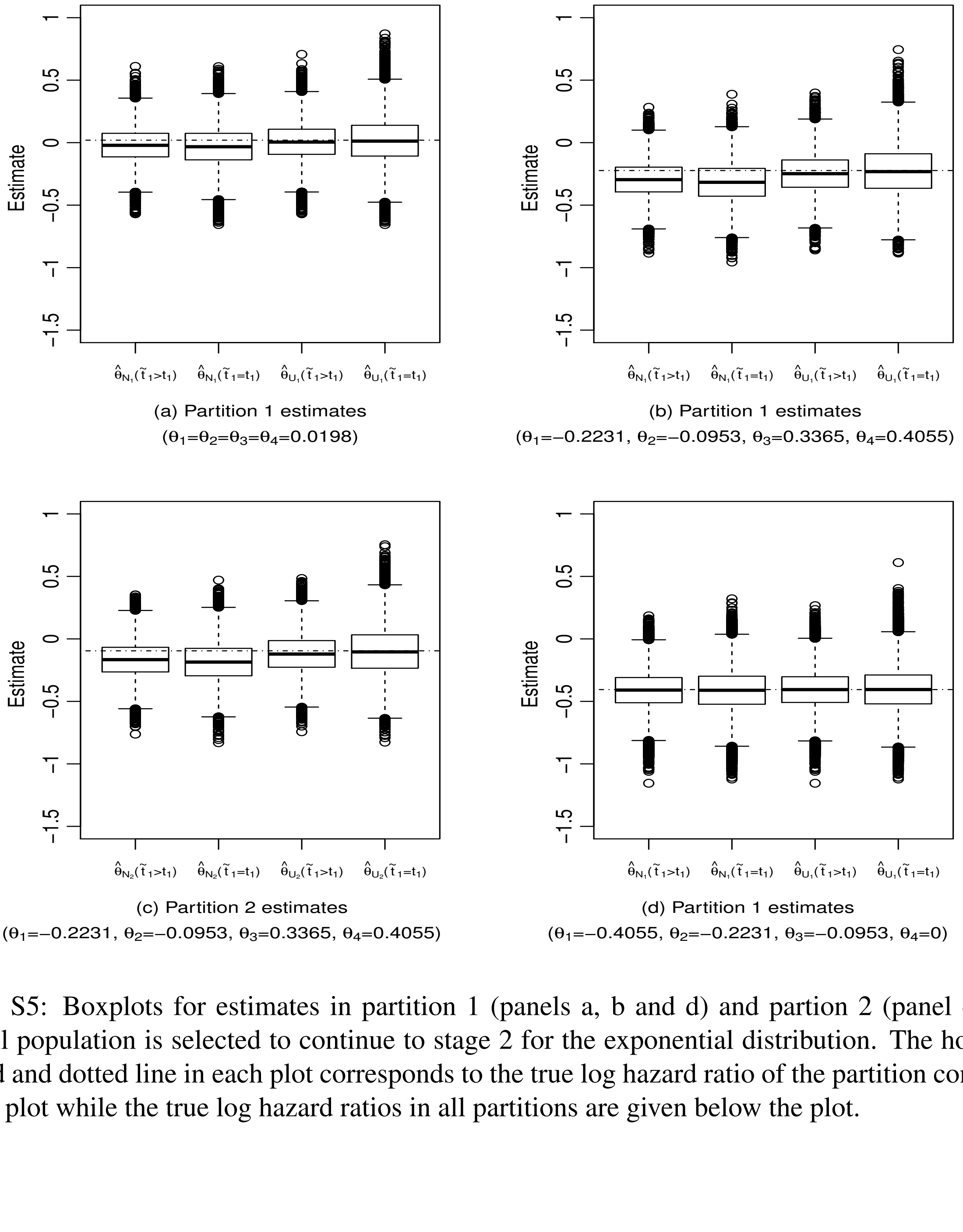

(a) Partition 1 estimates ($\theta_1=\theta_2=\theta_3=\theta_4=0.0198$)

(b) Partition 1 estimates ($\theta_1=-0.2231$, $\theta_2=-0.0953$, $\theta_3=0.3365$, $\theta_4=0.4055$)

(c) Partition 2 estimates ($\theta_1=-0.2231$, $\theta_2=-0.0953$, $\theta_3=0.3365$, $\theta_4=0.4055$)

(d) Partition 1 estimates ($\theta_1=-0.4055$, $\theta_2=-0.2231$, $\theta_3=-0.0953$, $\theta_4=0$)

Figure S5: Boxplots for estimates in partition 1 (panels a, b and d) and partion 2 (panel c) when the full population is selected to continue to stage 2 for the exponential distribution. The horizontal dashed and dotted line in each plot corresponds to the true log hazard ratio of the partition considered in that plot while the true log hazard ratios in all partitions are given below the plot.

Table S5: Coverage probability and type I error rate (Weibull distribution, $\gamma = 1.0$)

True log hazard ratios	Selected partitions ($\mathcal{S}$)	Coverage (Type I error rate)†	
		Naive	Duality
$\theta_1 = \theta_2 = \theta_3 = \theta_4 = 0.0198$	All	94.8 (4.4)	98.3 (1.7)
	1, 2 & 3	96.3 (2.9)	99.2 (0.8)
	1 & 2	96.1 (2.9)	99.2 (0.8)
	1	96.1 (3.0)	99.5 (0.4)
$\theta_1 = -0.2231$, $\theta_2 = -0.0953$, $\theta_3 = 0.3365$, $\theta_4 = 0.4055$	All	93.4 (6.2)	98.4 (1.5)
	1, 2 & 3	96.1 (2.9)	99.2 (0.4)
	1 & 2	96.2 (1.3)	99.1 (0.0)
	1	95.8 (1.5)	99.4 (0.0)
$\theta_1 = -0.4055$, $\theta_2 = -0.2231$, $\theta_3 = -0.0953$, $\theta_4 = 0$	All	95.5 (2.6)	98.7 (0.7)
	1, 2 & 3	93.7 (0.6)	97.9 (0.0)
	1 & 2	93.6 (0.4)	97.8 (0.0)
	1	93.9 (0.1)	98.4 (0.0)

† Type I error is the probability that at least one upper bound is less than the true value.

9 Weibull distribution ($\gamma = 1.5$) simulation results

Table S6: Simulated biases and mean squared errors of the estimators for the log hazard ratios (Weibull distribution, $\gamma = 1.5$)

Selected partitions ($\mathcal{S}$)	Partition	Simulated bias				Root mean squared error			
		$\hat{\theta}_{N_j}$		$\hat{\theta}_{U_j}$		$\hat{\theta}_{N_j}$		$\hat{\theta}_{U_j}$	
		$\tilde{t}_1 > t_1$	$\tilde{t}_1 = t_1$	$\tilde{t}_1 > t_1$	$\tilde{t}_1 = t_1$	$\tilde{t}_1 > t_1$	$\tilde{t}_1 = t_1$	$\tilde{t}_1 > t_1$	$\tilde{t}_1 = t_1$
True log hazard ratios: $\theta_1 = \theta_2 = \theta_3 = \theta_4 = 0.0198$									
All	1	0.0413	0.0532	0.0135	0.0009	0.1459	0.1651	0.1504	0.1847
	2	0.0405	0.0518	0.0128	-0.0005	0.1458	0.1647	0.1506	0.1851
	3	0.0418	0.0532	0.0135	0.0001	0.1474	0.1668	0.1522	0.1872
	4	0.0415	0.0526	0.0137	0.0003	0.1461	0.1656	0.1504	0.1852
1, 2 & 3	1	0.0219	0.0268	0.0082	0.0016	0.1313	0.1441	0.1493	0.1931
	2	0.0212	0.0270	0.0064	-0.0003	0.1305	0.1441	0.1477	0.1911
	3	0.0218	0.0279	0.0070	0.0014	0.1306	0.1441	0.1475	0.1911
1 & 2	1	0.0218	0.0267	0.0051	0.0000	0.1157	0.1256	0.1325	0.1600
	2	0.0225	0.0265	0.0071	0.0016	0.1177	0.1267	0.1332	0.1591
1	1	0.0231	0.0259	0.0047	0.0013	0.0935	0.0977	0.1047	0.1143
True log hazard ratios: $\theta_1 = -0.2231$, $\theta_2 = -0.0953$, $\theta_3 = 0.3365$, $\theta_4 = 0.4055$									
All	1	0.0759	0.0969	0.0266	0.0022	0.1669	0.1922	0.1650	0.2088
	2	0.0698	0.0897	0.0227	-0.0009	0.1616	0.1857	0.1614	0.2043
	3	0.0598	0.0750	0.0207	-0.0006	0.1459	0.1670	0.1456	0.1839
	4	0.0592	0.0752	0.0210	0.0021	0.1443	0.1647	0.1436	0.1796
1, 2 & 3	1	0.0278	0.0339	0.0088	-0.0002	0.1396	0.1532	0.1553	0.1942
	2	0.0266	0.0329	0.0087	0.0011	0.1362	0.1497	0.1510	0.1881
	3	0.0217	0.0266	0.0070	0.0007	0.1251	0.1372	0.1379	0.1705
1 & 2	1	-0.0123	-0.0138	-0.0044	-0.0012	0.1217	0.1303	0.1361	0.1577
	2	-0.0101	-0.0115	-0.0027	0.0003	0.1175	0.1262	0.1311	0.1520
1	1	-0.0126	-0.0144	-0.0009	0.0008	0.0943	0.0984	0.1059	0.1147
True log hazard ratios: $\theta_1 = -0.4055$, $\theta_2 = -0.2231$, $\theta_3 = -0.0953$, $\theta_4 = 0$									
All	1	0.0048	0.0060	0.0002	-0.0019	0.1495	0.1678	0.1524	0.1747
	2	0.0062	0.0079	0.0018	0.0004	0.1452	0.1630	0.1478	0.1694
	3	0.0053	0.0067	0.0012	-0.0003	0.1420	0.1592	0.1445	0.1651
	4	0.0058	0.0070	0.0019	0.0002	0.1396	0.1565	0.1419	0.1620
1, 2 & 3	1	-0.0682	-0.0822	-0.0255	-0.0032	0.1516	0.1694	0.1556	0.1981
	2	-0.0584	-0.0738	-0.0158	0.0036	0.1392	0.1564	0.1466	0.1860
	3	-0.0544	-0.0676	-0.0145	0.0055	0.1403	0.1552	0.1478	0.1849
1 & 2	1	-0.0714	-0.0818	-0.0260	-0.0088	0.1337	0.1462	0.1313	0.1537
	2	-0.0617	-0.0729	-0.0169	-0.0016	0.1335	0.1453	0.1375	0.1611
1	1	-0.0463	-0.0503	-0.0096	-0.0018	0.1052	0.1090	0.1077	0.1144

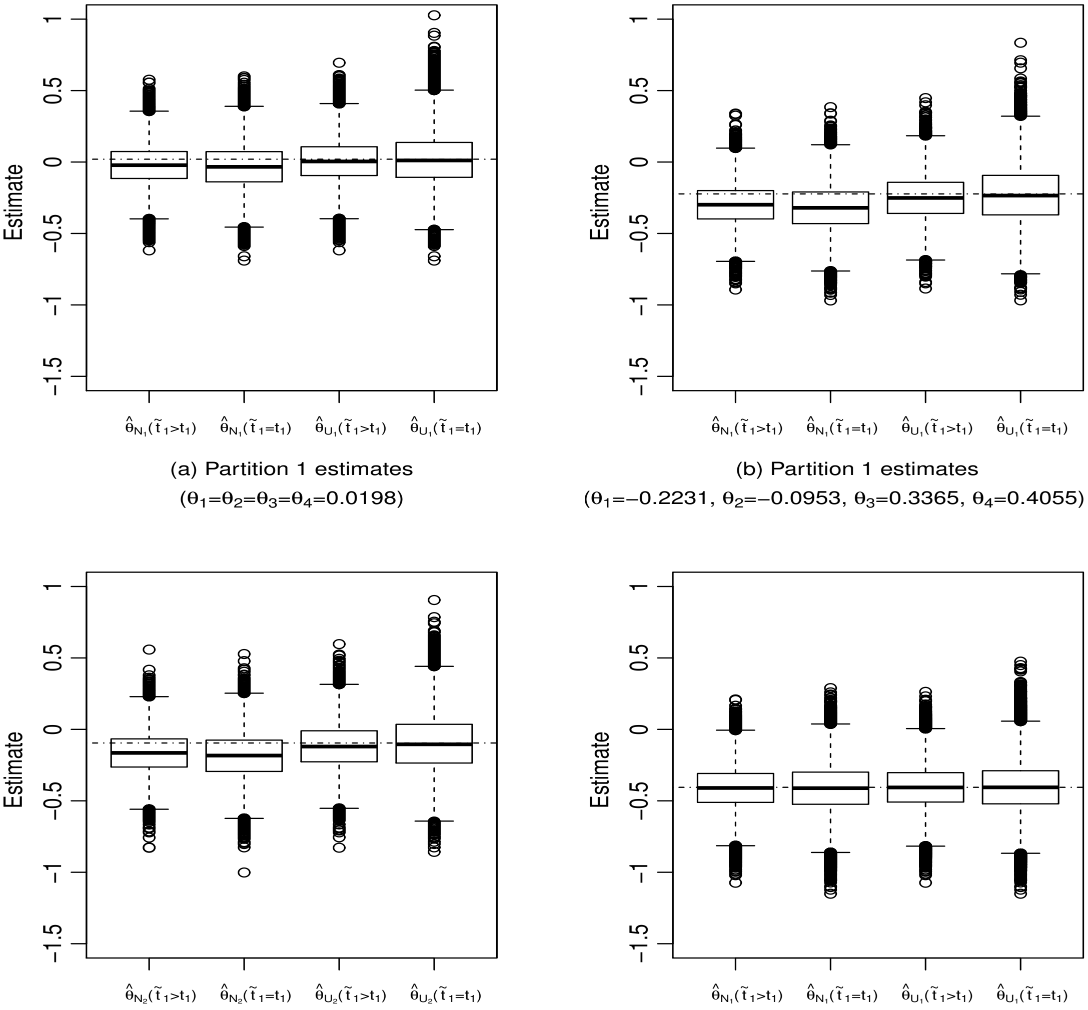

(a) Partition 1 estimates (θ_1=θ_2=θ_3=θ_4=0.0198)

(b) Partition 1 estimates (θ_1=−0.2231, θ_2=−0.0953, θ_3=0.3365, θ_4=0.4055)

(c) Partition 2 estimates (θ_1=−0.2231, θ_2=−0.0953, θ_3=0.3365, θ_4=0.4055)

(d) Partition 1 estimates (θ_1=−0.4055, θ_2=−0.2231, θ_3=−0.0953, θ_4=0)

Figure S6: Boxplots for estimates in partition 1 (panels a, b and d) and partion 2 (panel c) when the full population is selected to continue to stage 2 for the Weibull ($\gamma = 1.5$) distribution. The horizontal dashed and dotted line in each plot corresponds to the true log hazard ratio of the partition considered in that plot while the true log hazard ratios in all partitions are given below the plot.

Table S7: Coverage probability and type I error rate (Weibull distribution, $\gamma = 1.5$)

True log hazard ratios	Selected partitions ($\mathcal{S}$)	Coverage (Type I error rate)[†]	
		Naive	Duality
$\theta_1 = \theta_2 = \theta_3 = \theta_4 = 0.0198$	All	95.0 (4.3)	98.4 (1.6)
	1, 2 & 3	96.4 (2.6)	99.3 (0.7)
	1 & 2	96.2 (2.7)	99.3 (0.7)
	1	96.2 (2.9)	99.5 (0.5)
$\theta_1 = -0.2231$, $\theta_2 = -0.0953$, $\theta_3 = 0.3365$, $\theta_4 = 0.4055$	All	93.1 (6.6)	98.3 (1.6)
	1, 2 & 3	96.2 (3.0)	99.2 (0.5)
	1 & 2	96.4 (1.3)	99.2 (0.0)
	1	96.3 (1.4)	99.4 (0.0)
$\theta_1 = -0.4055$, $\theta_2 = -0.2231$, $\theta_3 = -0.0953$, $\theta_4 = 0$	All	95.4 (2.5)	98.7 (0.6)
	1, 2 & 3	93.7 (0.5)	98.1 (0.0)
	1 & 2	93.0 (0.4)	97.6 (0.0)
	1	94.3 (0.3)	98.5 (0.0)

[†] Type I error is the probability that at least one upper bound is less than the true value.

10 Slower recruitment rate with Weibull ($\gamma = 0.5$) distribution simulation results

Table S8: Simulated biases and mean squared errors of the estimators for the log hazard ratios with the Weibull ($\gamma = 0.5$) distribution when the trial recruits for approximately 2 years in each stage (slower recruitment rate)

Selected partitions ($\mathcal{S}$)	Partition	Simulated bias				Root mean squared error			
		$\hat{\theta}_{N_j}$		$\hat{\theta}_{U_j}$		$\hat{\theta}_{N_j}$		$\hat{\theta}_{U_j}$	
		$\tilde{t}_1 > t_1$	$\tilde{t}_1 = t_1$	$\tilde{t}_1 > t_1$	$\tilde{t}_1 = t_1$	$\tilde{t}_1 > t_1$	$\tilde{t}_1 = t_1$	$\tilde{t}_1 > t_1$	$\tilde{t}_1 = t_1$
True log hazard ratios: $\theta_1 = \theta_2 = \theta_3 = \theta_4 = 0.0198$									
All	1	0.0410	0.0519	0.0129	-0.0010	0.1473	0.1657	0.1521	0.1861
	2	0.0406	0.0520	0.0126	-0.0006	0.1468	0.1659	0.1516	0.1859
	3	0.0414	0.0525	0.0132	0.0000	0.1464	0.1653	0.1511	0.1849
	4	0.0409	0.0516	0.0131	-0.0004	0.1463	0.1647	0.1509	0.1845
1, 2 & 3	1	0.0230	0.0271	0.0083	-0.0003	0.1305	0.1432	0.1488	0.1924
	2	0.0183	0.0243	0.0037	-0.0020	0.1318	0.1447	0.1499	0.1934
	3	0.0220	0.0267	0.0077	0.0004	0.1329	0.1455	0.1504	0.1937
1 & 2	1	0.0227	0.0263	0.0056	-0.0018	0.1170	0.1265	0.1324	0.1595
	2	0.0207	0.0243	0.0051	-0.0010	0.1171	0.1250	0.1335	0.1584
1	1	0.0214	0.0239	0.0027	-0.0011	0.0939	0.0982	0.1055	0.1154
True log hazard ratios: $\theta_1 = -0.2231$, $\theta_2 = -0.0953$, $\theta_3 = 0.3365$, $\theta_4 = 0.4055$									
All	1	0.0752	0.0960	0.0264	0.0020	0.1666	0.1920	0.1652	0.2098
	2	0.0733	0.0928	0.0266	0.0027	0.1631	0.1870	0.1612	0.2026
	3	0.0581	0.0740	0.0176	-0.0031	0.1473	0.1670	0.1486	0.1864
	4	0.0595	0.0753	0.0201	0.0012	0.1471	0.1666	0.1471	0.1818
1, 2 & 3	1	0.0268	0.0332	0.0077	-0.0008	0.1390	0.1525	0.1548	0.1930
	2	0.0264	0.0321	0.0085	0.0001	0.1351	0.1494	0.1498	0.1883
	3	0.0217	0.0276	0.0058	-0.0004	0.1289	0.1400	0.1428	0.1747
1 & 2	1	-0.0111	-0.0128	-0.0036	-0.0009	0.1201	0.1296	0.1345	0.1571
	2	-0.0099	-0.0118	-0.0024	0.0001	0.1182	0.1272	0.1320	0.1537
1	1	-0.0119	-0.0136	-0.0001	0.0018	0.0937	0.0982	0.1055	0.1150
True log hazard ratios: $\theta_1 = -0.4055$, $\theta_2 = -0.2231$, $\theta_3 = -0.0953$, $\theta_4 = 0$									
All	1	0.0069	0.0071	0.0023	-0.0010	0.1504	0.1684	0.1533	0.1753
	2	0.0062	0.0075	0.0019	0.0001	0.1457	0.1633	0.1483	0.1695
	3	0.0059	0.0071	0.0018	0.0000	0.1425	0.1593	0.1449	0.1650
	4	0.0055	0.0072	0.0015	0.0003	0.1403	0.1573	0.1427	0.1629
1, 2 & 3	1	-0.0643	-0.0802	-0.0206	0.0001	0.1453	0.1641	0.1509	0.1930
	2	-0.0598	-0.0742	-0.0194	-0.0002	0.1436	0.1598	0.1515	0.1919
	3	-0.0588	-0.0722	-0.0199	-0.0009	0.1413	0.1595	0.1468	0.1877
1 & 2	1	-0.0657	-0.0762	-0.0187	-0.0001	0.1356	0.1488	0.1374	0.1640
	2	-0.0613	-0.0703	-0.0186	-0.0019	0.1315	0.1438	0.1338	0.158
1	1	-0.0489	-0.0518	-0.0127	-0.0034	0.1079	0.1124	0.1099	0.1182

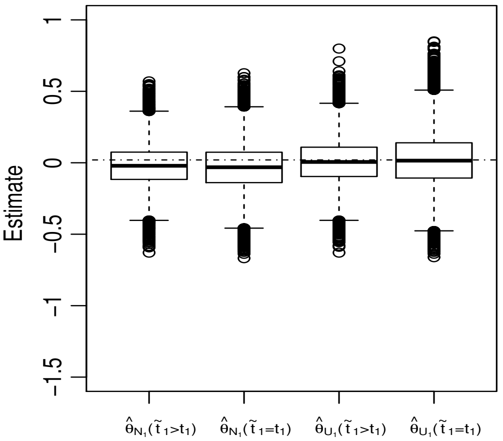

(a) Partition 1 estimates
(θ_1=θ_2=θ_3=θ_4=0.0198)

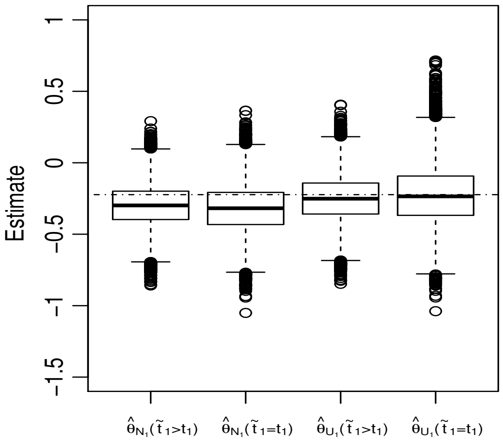

(b) Partition 1 estimates
(θ_1=−0.2231, θ_2=−0.0953, θ_3=0.3365, θ_4=0.4055)

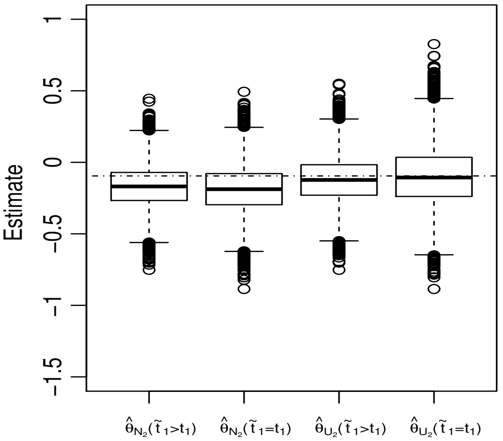

(c) Partition 2 estimates
(θ_1=−0.2231, θ_2=−0.0953, θ_3=0.3365, θ_4=0.4055)

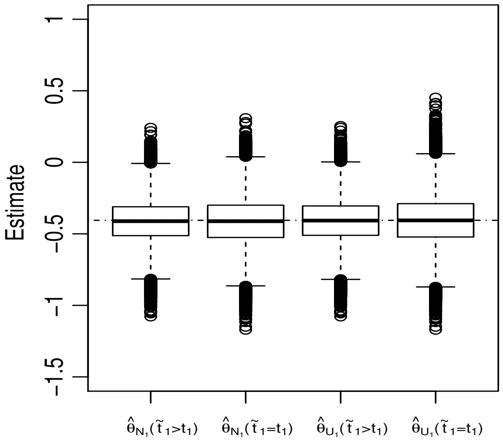

(d) Partition 1 estimates
(θ_1=−0.4055, θ_2=−0.2231, θ_3=−0.0953, θ_4=0)

Figure S7: Boxplots for estimates in partition 1 (panels a, b and d) and partion 2 (panel c) when the full population is selected to continue to stage 2 for Weibull ($\gamma = 0.5$) distribution and with the trial recruiting for approximately 2 years in each stage (Slower recruitment rate). The horizontal dashed and dotted line in each plot corresponds to the true log hazard ratio of the partition considered in that plot while the true log hazard ratios in all partitions are given below the plot.

Table S9: Coverage probability and type I error rate (Weibull distribution, $\gamma = 0.5$)

True log hazard ratios	Selected partitions ($\mathcal{S}$)	Coverage (Type I error rate)†	
		Naive	Duality
$\theta_1 = \theta_2 = \theta_3 = \theta_4 = 0.0198$	All	94.8 (4.5)	98.3 (1.7)
	1, 2 & 3	96.4 (2.6)	99.2 (0.8)
	1 & 2	96.3 (2.8)	99.2 (0.8)
	1	95.8 (3.2)	99.5 (0.5)
$\theta_1 = -0.2231$, $\theta_2 = -0.0953$, $\theta_3 = 0.3365$, $\theta_4 = 0.4055$	All	93.1 (6.5)	98.3 (1.6)
	1, 2 & 3	96.0 (3.0)	99.1 (0.6)
	1 & 2	96.3 (1.4)	99.2 (0.0)
	1	96.2 (1.2)	99.2 (0.0)
$\theta_1 = -0.4055$, $\theta_2 = -0.2231$, $\theta_3 = -0.0953$, $\theta_4 = 0$	All	95.3 (2.7)	98.7 (0.7)
	1, 2 & 3	94.0 (0.2)	98.0 (0.0)
	1 & 2	91.6 (0.7)	98.0 (0.0)
	1	93.4 (0.5)	98.3 (0.0)

† Type I error is the probability that at least one upper bound is less than the true value.

11 Exponential ($\gamma = 1.0$) distribution results with $\tilde{t}_1$ corresponding to 250 days after the interim analysis simulation results

Table S10: Simulated biases and mean squared errors of the estimators for the log hazard ratios (Exponential distribution) with $\tilde{t}_1$ corresponding to 250 days after the interim analysis)

		Simulated bias				Root mean squared error			
Selected		$\hat{\theta}_{N_j}$		$\hat{\theta}_{U_j}$		$\hat{\theta}_{N_j}$		$\hat{\theta}_{U_j}$	
partitions ($\mathcal{S}$)	Partition	$\tilde{t}_1 > t_1$	$\tilde{t}_1 = t_1$	$\tilde{t}_1 > t_1$	$\tilde{t}_1 = t_1$	$\tilde{t}_1 > t_1$	$\tilde{t}_1 = t_1$	$\tilde{t}_1 > t_1$	$\tilde{t}_1 = t_1$
True log hazard ratios: $\theta_1 = \theta_2 = \theta_3 = \theta_4 = 0.0198$									
All	1	0.0341	0.0532	0.0097	0.0009	0.1313	0.1648	0.1350	0.1841
	2	0.0334	0.0521	0.0090	-0.0004	0.1313	0.1648	0.1350	0.1847
	3	0.0334	0.0515	0.0090	-0.0012	0.1315	0.1648	0.1354	0.1851
	4	0.0335	0.0522	0.0091	-0.0002	0.1323	0.1660	0.1362	0.1855
1, 2 & 3	1	0.0181	0.0299	0.0043	0.0034	0.1202	0.1434	0.1353	0.1912
	2	0.0181	0.0253	0.0054	-0.0016	0.1193	0.1417	0.1338	0.1904
	3	0.0174	0.0247	0.0043	-0.0028	0.1218	0.1441	0.1365	0.1927
1 & 2	1	0.0165	0.0234	0.0017	-0.0031	0.1087	0.1241	0.1216	0.1571
	2	0.0195	0.0276	0.0044	0.0009	0.1106	0.1266	0.1239	0.1590
1	1	0.0201	0.0249	0.0030	0.0001	0.0896	0.0981	0.0996	0.1151
True log hazard ratios: $\theta_1 = -0.2231$, $\theta_2 = -0.0953$, $\theta_3 = 0.3365$, $\theta_4 = 0.4055$									
All	1	0.0585	0.0932	0.0150	-0.0030	0.1470	0.1909	0.1468	0.2095
	2	0.0569	0.0907	0.0152	-0.0008	0.1430	0.1846	0.1434	0.2026
	3	0.0477	0.0753	0.0128	0.0001	0.1321	0.1680	0.1326	0.1836
	4	0.0462	0.0741	0.0114	0.0002	0.1298	0.1635	0.1312	0.1797
1, 2 & 3	1	0.0228	0.0326	0.0062	-0.0015	0.1279	0.1545	0.1407	0.1964
	2	0.0218	0.0321	0.0061	0.0002	0.1248	0.1499	0.1372	0.1901
	3	0.0184	0.0278	0.0046	0.0007	0.1159	0.1378	0.1265	0.1705
1 & 2	1	-0.0102	-0.0141	-0.0028	-0.0011	0.1125	0.1295	0.1246	0.1571
	2	-0.0085	-0.0119	-0.0018	-0.0004	0.1109	0.1270	0.1225	0.1534
1	1	-0.0135	-0.0163	-0.0029	-0.0013	0.0914	0.0998	0.1013	0.1157
True log hazard ratios: $\theta_1 = -0.4055$, $\theta_2 = -0.2231$, $\theta_3 = -0.0953$, $\theta_4 = 0$									
All	1	0.0043	0.0050	0.0004	-0.0030	0.1363	0.1678	0.1386	0.1748
	2	0.0053	0.0075	0.0016	0.0001	0.1324	0.1632	0.1344	0.1695
	3	0.0049	0.0075	0.0014	0.0004	0.1282	0.1589	0.1302	0.1648
	4	0.0042	0.0067	0.0007	-0.0002	0.1261	0.1554	0.1280	0.1611
1, 2 & 3	1	-0.0522	-0.0817	-0.0126	-0.0022	0.1339	0.1664	0.1393	0.1952
	2	-0.0504	-0.0738	-0.0142	0.0000	0.1305	0.1599	0.1367	0.1925
	3	-0.0488	-0.0684	-0.0139	0.0041	0.1279	0.1546	0.1335	0.1829
1 & 2	1	-0.0542	-0.0746	-0.0111	0.0019	0.1229	0.1452	0.1262	0.1612
	2	-0.0550	-0.0731	-0.0164	-0.0050	0.1212	0.1452	0.1233	0.1587
1	1	-0.0424	-0.0501	-0.0072	0.0004	0.0989	0.1108	0.1017	0.1190

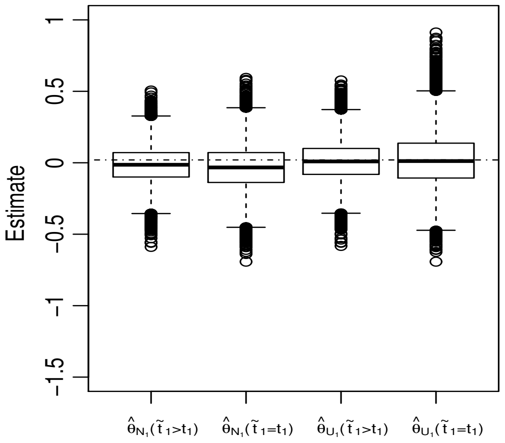

(a) Partition 1 estimates
($\theta_1=\theta_2=\theta_3=\theta_4=0.0198$)

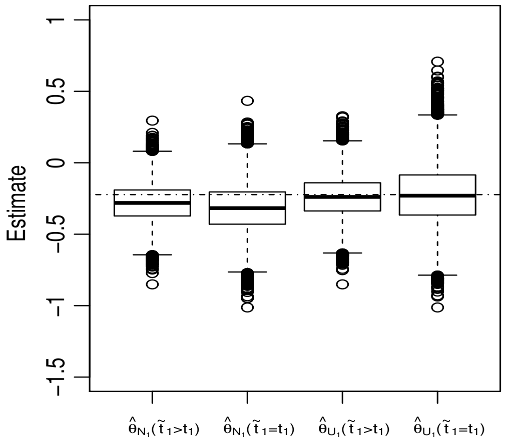

(b) Partition 1 estimates
($\theta_1=-0.2231$, $\theta_2=-0.0953$, $\theta_3=0.3365$, $\theta_4=0.4055$)

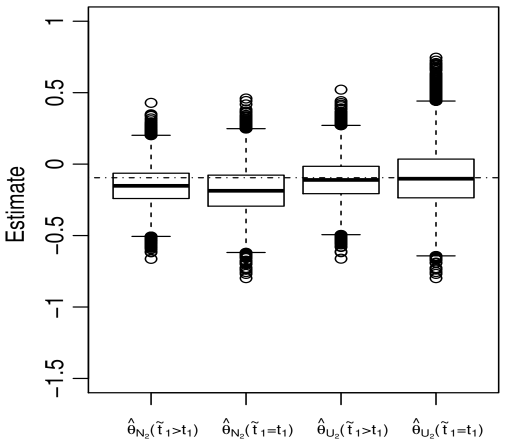

(c) Partition 2 estimates
($\theta_1=-0.2231$, $\theta_2=-0.0953$, $\theta_3=0.3365$, $\theta_4=0.4055$)

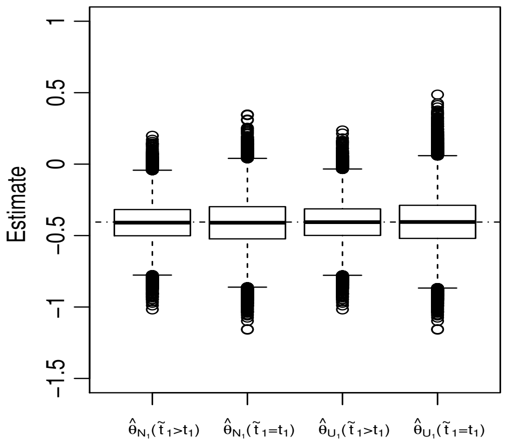

(d) Partition 1 estimates
($\theta_1=-0.4055$, $\theta_2=-0.2231$, $\theta_3=-0.0953$, $\theta_4=0$)

Figure S8: Boxplots for estimates in partition 1 (panels a, b and d) and partion 2 (panel c) when the full population is selected to continue to stage 2 for exponential distribution ($\gamma = 1.0$) and $\tilde{t}_1$ corresponds to 250 days after the interim analysis. The horizontal dashed and dotted line in each plot corresponds to the true log hazard ratio of the partition considered in that plot while the true log hazard ratios in all partitions are given below the plot.

Table S11: Coverage probability and type I error rate (Weibull distribution, $\gamma = 1.0$)

True log hazard ratios	Selected partitions ($\mathcal{S}$)	Coverage (Type I error rate)† Naive	Duality
$\theta_1 = \theta_2 = \theta_3 = \theta_4 = 0.0198$	All	94.9 (4.2)	98.5 (1.5)
	1, 2 & 3	96.3 (2.6)	99.1 (0.9)
	1 & 2	96.3 (2.7)	99.1 (0.9)
	1	96.2 (3.0)	99.4 (0.6)
$\theta_1 = -0.2231$, $\theta_2 = -0.0953$, $\theta_3 = 0.3365$, $\theta_4 = 0.4055$	All	93.6 (5.9)	98.4 (1.4)
	1, 2 & 3	95.8 (3.0)	99.0 (0.5)
	1 & 2	96.2 (1.5)	99.1 (0.0)
	1	96.0 (1.2)	99.0 (0.0)
$\theta_1 = -0.4055$, $\theta_2 = -0.2231$, $\theta_3 = -0.0953$, $\theta_4 = 0$	All	95.4 (2.5)	98.6 (0.7)
	1, 2 & 3	94.0 (0.5)	98.3 (0.0)
	1 & 2	92.6 (0.5)	97.9 (0.0)
	1	93.6 (1.1)	98.1 (0.0)

† Type I error is the probability that at least one upper bound is less than the true value.

12 Weibull ($\gamma = 1.5$) distribution results with $\tilde{t}_1$ corresponding to 150 days after the interim analysis simulation results

Table S12: Simulated biases and mean squared errors of the estimators for the log hazard ratios (Weibull ($\gamma = 1.5$) distribution) with $\tilde{t}_1$ corresponding to 150 days after the interim analysis)

		Simulated bias				Root mean squared error			
Selected		$\hat{\theta}_{N_j}$		$\hat{\theta}_{U_j}$		$\hat{\theta}_{N_j}$		$\hat{\theta}_{U_j}$	
partitions ($\mathcal{S}$)	Partition	$\tilde{t}_1 > t_1$	$\tilde{t}_1 = t_1$	$\tilde{t}_1 > t_1$	$\tilde{t}_1 = t_1$	$\tilde{t}_1 > t_1$	$\tilde{t}_1 = t_1$	$\tilde{t}_1 > t_1$	$\tilde{t}_1 = t_1$
True log hazard ratios: $\theta_1 = \theta_2 = \theta_3 = \theta_4 = 0.0198$									
All	1	0.0336	0.0528	0.0085	-0.0007	0.1331	0.1661	0.1374	0.1866
	2	0.0349	0.0529	0.0102	0.0003	0.1331	0.1656	0.1370	0.1855
	3	0.0337	0.0519	0.0091	-0.0002	0.1327	0.1647	0.1368	0.1845
	4	0.0343	0.0531	0.0097	0.0008	0.1332	0.1653	0.1370	0.1850
1, 2 & 3	1	0.0164	0.0247	0.0032	-0.0024	0.1204	0.1433	0.1352	0.1928
	2	0.0177	0.0259	0.0044	-0.0013	0.1189	0.1417	0.1333	0.1891
	3	0.0203	0.0274	0.0075	0.0008	0.1215	0.1434	0.1364	0.1927
1 & 2	1	0.0186	0.0258	0.0044	0.0005	0.1100	0.1268	0.1232	0.1600
	2	0.0180	0.0270	0.0027	0.0006	0.1090	0.1250	0.1229	0.1581
1	1	0.0206	0.0251	0.0035	0.0003	0.0897	0.0979	0.0995	0.1145
True log hazard ratios: $\theta_1 = -0.2231$, $\theta_2 = -0.0953$, $\theta_3 = 0.3365$, $\theta_4 = 0.4055$									
All	1	0.0609	0.0953	0.0169	-0.0010	0.1494	0.1917	0.1493	0.2110
	2	0.0593	0.0921	0.0173	0.0006	0.1462	0.1876	0.1459	0.2054
	3	0.0501	0.0767	0.0150	0.0015	0.1324	0.1676	0.1329	0.1831
	4	0.0499	0.0753	0.0155	0.0016	0.1310	0.1653	0.1312	0.1809
1, 2 & 3	1	0.0229	0.0341	0.0057	-0.0002	0.1291	0.1538	0.1420	0.1946
	2	0.0216	0.0322	0.0058	0.0008	0.1260	0.1501	0.1386	0.1893
	3	0.0191	0.0273	0.0054	0.0004	0.1161	0.1376	0.1265	0.1703
1 & 2	1	-0.0105	-0.0143	-0.0033	-0.0021	0.1134	0.1295	0.1258	0.1576
	2	-0.0090	-0.0119	-0.0019	0.0003	0.1115	0.1269	0.1232	0.1534
1	1	-0.0120	-0.0145	-0.0014	0.0005	0.0921	0.0992	0.1023	0.1154
True log hazard ratios: $\theta_1 = -0.4055$, $\theta_2 = -0.2231$, $\theta_3 = -0.0953$, $\theta_4 = 0$									
All	1	0.0029	0.0046	-0.0011	-0.0035	0.1369	0.1674	0.1393	0.1747
	2	0.0045	0.0069	0.0007	-0.0006	0.1328	0.1632	0.1350	0.1695
	3	0.0050	0.0073	0.0014	0.0001	0.1301	0.1593	0.1321	0.1652
	4	0.0044	0.0064	0.0009	-0.0004	0.1279	0.1566	0.1298	0.1623
1, 2 & 3	1	-0.0570	-0.0828	-0.0177	-0.0037	0.1377	0.1695	0.1440	0.2018
	2	-0.0509	-0.0737	-0.0147	-0.0011	0.1316	0.1601	0.1392	0.1935
	3	-0.0477	-0.0691	-0.0123	0.0019	0.1277	0.1551	0.1340	0.1870
1 & 2	1	-0.0566	-0.0753	-0.0152	-0.0028	0.1254	0.1467	0.1280	0.1600
	2	-0.0528	-0.0739	-0.0121	-0.0038	0.1209	0.1430	0.1248	0.1577
1	1	-0.0351	-0.0440	0.0001	0.0058	0.0911	0.1024	0.0949	0.1102

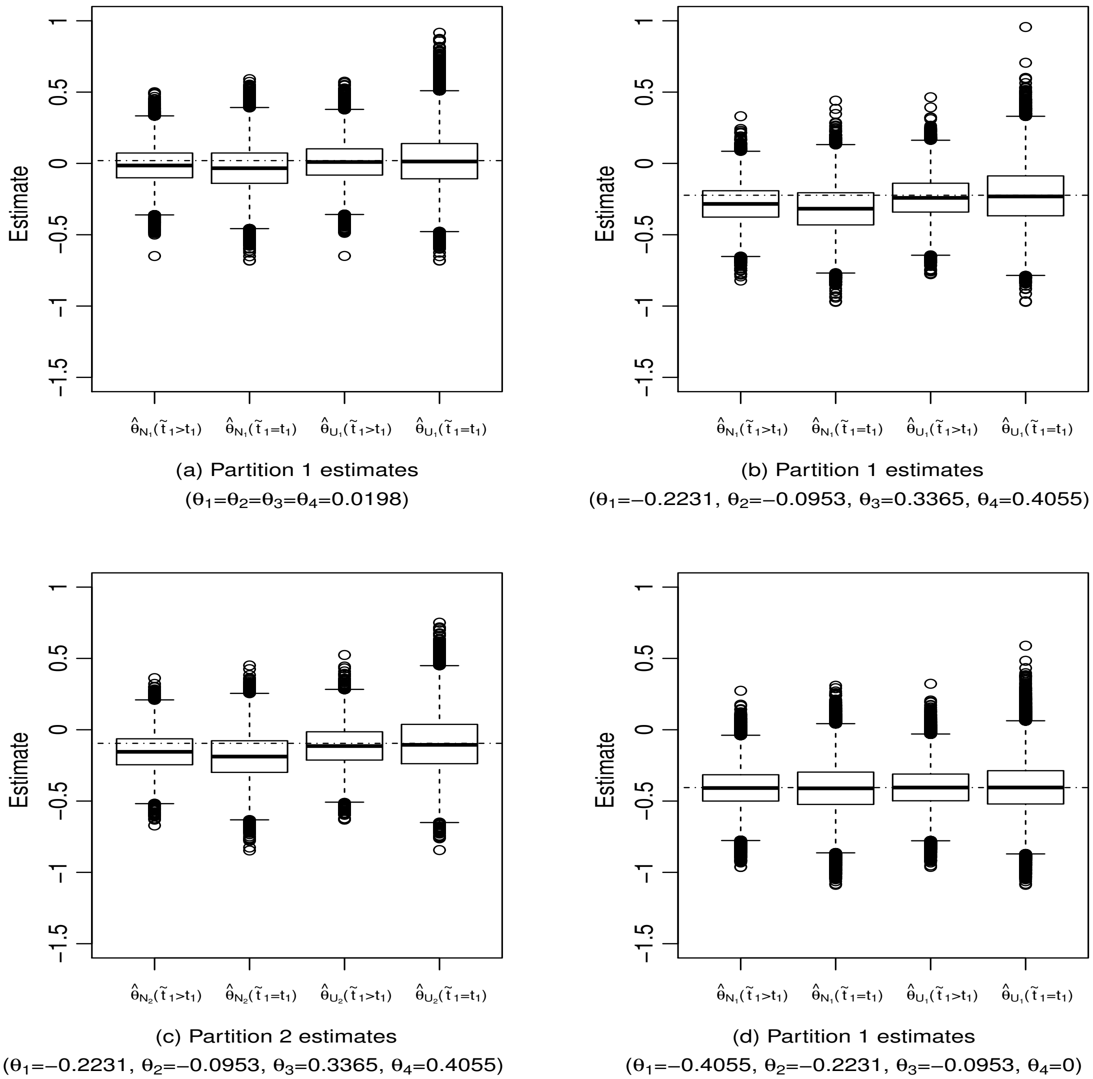

(a) Partition 1 estimates (θ_1=θ_2=θ_3=θ_4=0.0198)

(b) Partition 1 estimates (θ_1=−0.2231, θ_2=−0.0953, θ_3=0.3365, θ_4=0.4055)

(c) Partition 2 estimates (θ_1=−0.2231, θ_2=−0.0953, θ_3=0.3365, θ_4=0.4055)

(d) Partition 1 estimates (θ_1=−0.4055, θ_2=−0.2231, θ_3=−0.0953, θ_4=0)

Figure S9: Boxplots for estimates in partition 1 (panels a, b and d) and partion 2 (panel c) when the full population is selected to continue to stage 2 for Weibull ($\gamma = 1.5$) distribution and $\tilde{t}_1$ corresponds to 150 days after the interim analysis. The horizontal dashed and dotted line in each plot corresponds to the true log hazard ratio of the partition considered in that plot while the true log hazard ratios in all partitions are given below the plot.

Table S13: Coverage probability and type I error rate (Weibull distribution, $\gamma = 1.0$)

True log hazard ratios	Selected partitions ($\mathcal{S}$)	Coverage (Type I error rate)† Naive	Coverage (Type I error rate)† Duality
$\theta_1 = \theta_2 = \theta_3 = \theta_4 = 0.0198$	All	94.8 (4.3)	98.4 (1.6)
	1, 2 & 3	96.6 (2.5)	99.2 (0.8)
	1 & 2	96.0 (2.8)	99.2 (0.8)
	1	95.8 (3.1)	99.3 (0.7)
$\theta_1 = -0.2231$, $\theta_2 = -0.0953$, $\theta_3 = 0.3365$, $\theta_4 = 0.4055$	All	93.4 (6.1)	98.3 (1.5)
	1, 2 & 3	95.9 (3.0)	98.9 (0.5)
	1 & 2	96.2 (1.5)	99.1 (0.0)
	1	95.8 (1.6)	99.3 (0.0)
$\theta_1 = -0.4055$, $\theta_2 = -0.2231$, $\theta_3 = -0.0953$, $\theta_4 = 0$	All	95.4 (2.6)	98.7 (0.7)
	1, 2 & 3	94.3 (0.4)	98.2 (0.0)
	1 & 2	92.4 (0.4)	97.4 (0.0)
	1	95.8 (0.4)	99.0 (0.0)

† Type I error is the probability that at least one upper bound is less than the true value.

13 Simulation results when there are fewer events in a trial

Table S14: Simulated biases and mean squared errors of the estimators for the log hazard ratios with the Weibull ($\gamma = 0.5$) distribution when there are fewer events (200 events at interim analysis, 200 events from stage 2 patients and $\tilde{t}_1$ corresponding to 300 days after interim analysis which approximately an average of 30 additional events in selected partitions from stage 1 patients without events at the interim analysis).

Selected partitions ($\mathcal{S}$)	Partition	Simulated bias				Root mean squared error			
		$\hat{\theta}_{N_j}$		$\hat{\theta}_{U_j}$		$\hat{\theta}_{N_j}$		$\hat{\theta}_{U_j}$	
		$\tilde{t}_1 > t_1$	$\tilde{t}_1 = t_1$	$\tilde{t}_1 > t_1$	$\tilde{t}_1 = t_1$	$\tilde{t}_1 > t_1$	$\tilde{t}_1 = t_1$	$\tilde{t}_1 > t_1$	$\tilde{t}_1 = t_1$
True log hazard ratios: $\theta_1 = \theta_2 = \theta_3 = \theta_4 = 0.0198$									
All	1	0.0483	0.0617	0.0154	-0.0009	0.1779	0.2020	0.1837	0.2266
	2	0.0495	0.0636	0.0164	0.0009	0.1796	0.2041	0.1853	0.2285
	3	0.0486	0.0635	0.0155	0.0008	0.1780	0.2033	0.1840	0.2281
	4	0.0495	0.0634	0.0167	0.0015	0.1779	0.2027	0.1829	0.2257
1, 2 & 3	1	0.0265	0.0305	0.0111	0.0011	0.1580	0.1750	0.1795	0.2367
	2	0.0258	0.0336	0.0085	0.0026	0.1611	0.1770	0.1825	0.2355
	3	0.0263	0.0330	0.0106	0.0044	0.1586	0.1741	0.1804	0.2339
1 & 2	1	0.0260	0.0299	0.0072	-0.0011	0.1440	0.1549	0.1637	0.1964
	2	0.0251	0.0300	0.0063	-0.0005	0.1428	0.1537	0.1629	0.1952
1	1	0.0264	0.0296	0.0047	0.0003	0.1137	0.1191	0.1276	0.1399
True log hazard ratios: $\theta_1 = -0.2231$, $\theta_2 = -0.0953$, $\theta_3 = 0.3365$, $\theta_4 = 0.4055$									
All	1	0.0832	0.1066	0.0285	-0.0001	0.2002	0.2307	0.2007	0.2549
	2	0.0774	0.1010	0.0252	0.0003	0.1940	0.2240	0.1954	0.2474
	3	0.0667	0.0859	0.0228	0.0017	0.1766	0.2026	0.1773	0.2223
	4	0.0645	0.0830	0.0217	0.0010	0.1754	0.2002	0.1772	0.2210
1, 2 & 3	1	0.0289	0.0353	0.0080	-0.0032	0.1689	0.1864	0.1898	0.2417
	2	0.0287	0.0363	0.0086	0.0002	0.1655	0.1832	0.1856	0.2361
	3	0.0241	0.0309	0.0070	0.0006	0.1525	0.1678	0.1693	0.2109
1 & 2	1	-0.0080	-0.0097	-0.0020	-0.0002	0.1471	0.1585	0.1659	0.1950
	2	-0.0068	-0.0079	-0.0010	0.0013	0.1439	0.1556	0.1618	0.1903
1	1	-0.0109	-0.0113	-0.0030	-0.0008	0.1143	0.1195	0.1289	0.1402
True log hazard ratios: $\theta_1 = -0.4055$, $\theta_2 = -0.2231$, $\theta_3 = -0.0953$, $\theta_4 = 0$									
All	1	0.0097	0.0123	0.0011	-0.0032	0.1824	0.2051	0.1871	0.2172
	2	0.0112	0.0142	0.0032	0.0000	0.1765	0.1996	0.1806	0.2102
	3	0.0110	0.0140	0.0033	0.0004	0.1735	0.1954	0.1775	0.2057
	4	0.0106	0.0132	0.0032	0.0000	0.1691	0.1908	0.1729	0.2007
1, 2 & 3	1	-0.0631	-0.0796	-0.0233	-0.0067	0.1771	0.1975	0.1889	0.2413
	2	-0.0556	-0.0692	-0.0180	0.0004	0.1658	0.1845	0.1815	0.2343
	3	-0.0539	-0.0673	-0.0171	0.0003	0.1649	0.1850	0.1780	0.2283
1 & 2	1	-0.0623	-0.0725	-0.0180	-0.0003	0.1599	0.1709	0.1691	0.1964
	2	-0.0558	-0.0661	-0.0152	-0.0009	0.1487	0.1623	0.1585	0.1899
1	1	-0.0400	-0.0440	-0.0072	-0.0003	0.1204	0.1250	0.1282	0.1380

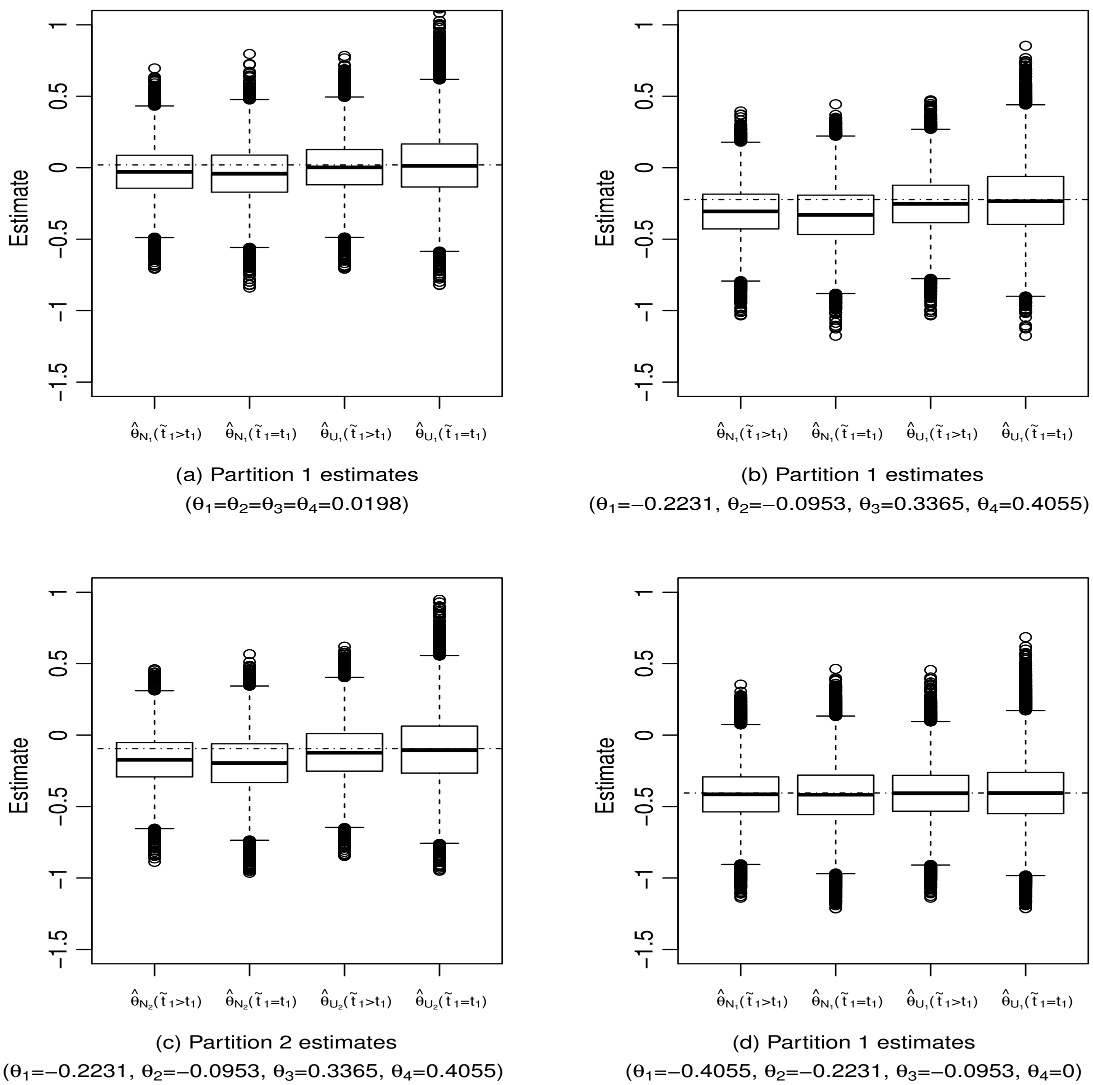

Figure S10: Boxplots for estimates in partition 1 (panels a, b and d) and partion 2 (panel c) when the full population is selected to continue to stage 2. Results are for Weibull ($\gamma = 0.5$) distribution when there are fewer events (200 events at interim analysis, 200 events from stage 2 patients and an average of 40 additional events in selected partitions from the censored stage 1 observations). The horizontal dashed and dotted line in each plot corresponds to the true log hazard ratio of the partition considered in that plot while the true log hazard ratios in all partitions are given below the plot.

Table S15: Coverage probability and type I error rate (Weibull distribution, $\gamma = 0.5$)

True log hazard ratios	Selected partitions ($\mathcal{S}$)	Coverage (Type I error rate)[†] Naive	Duality
$\theta_1 = \theta_2 = \theta_3 = \theta_4 = 0.0198$	All	94.8 (4.4)	98.3 (1.7)
	1, 2 & 3	96.3 (2.6)	99.1 (0.9)
	1 & 2	96.4 (2.6)	99.2 (0.8)
	1	96.0 (3.0)	99.5 (0.5)
$\theta_1 = -0.2231$, $\theta_2 = -0.0953$, $\theta_3 = 0.3365$, $\theta_4 = 0.4055$	All	93.5 (6.0)	98.5 (1.4)
	1, 2 & 3	96.2 (2.8)	99.2 (0.4)
	1 & 2	96.3 (1.6)	99.3 (0.0)
	1	96.2 (1.4)	99.7 (0.0)
$\theta_1 = -0.4055$, $\theta_2 = -0.2231$, $\theta_3 = -0.0953$, $\theta_4 = 0$	All	95.5 (2.7)	98.9 (0.6)
	1, 2 & 3	94.9 (0.6)	98.4 (0.0)
	1 & 2	95.1 (0.4)	98.3 (0.0)
	1	95.1 (0.6)	99.3 (0.0)

[†] Type I error is the probability that at least one upper bound is less than the true value.

14 Simulation results when subpopulation selection is performed earlier in the trial

Table S16: Simulated biases and mean squared errors of the estimators for the log hazard ratios when subpopulation selection is performed earlier in the trial (200 events at interim analysis, 400 patients from stage 2 patients and an average of 40 additional events in selected partitions from the censored stage 1 observations)

Selected partitions ($\mathcal{S}$)	Partition	Simulated bias				Root mean squared error			
		$\hat{\theta}_{N_j}$		$\hat{\theta}_{U_j}$		$\hat{\theta}_{N_j}$		$\hat{\theta}_{U_j}$	
		$\tilde{t}_1 > t_1$	$\tilde{t}_1 = t_1$	$\tilde{t}_1 > t_1$	$\tilde{t}_1 = t_1$	$\tilde{t}_1 > t_1$	$\tilde{t}_1 = t_1$	$\tilde{t}_1 > t_1$	$\tilde{t}_1 = t_1$
True log hazard ratios: $\theta_1 = \theta_2 = \theta_3 = \theta_4 = 0.0198$									
All	1	0.0331	0.0416	0.0076	-0.0002	0.1465	0.1645	0.1503	0.1755
	2	0.0329	0.0414	0.0072	-0.0004	0.1466	0.1638	0.1509	0.1755
	3	0.0331	0.0420	0.0076	0.0005	0.1457	0.1637	0.1500	0.1753
	4	0.0333	0.0417	0.0076	-0.0003	0.1466	0.1639	0.1508	0.1755
1, 2 & 3	1	0.0154	0.0186	0.0039	0.0010	0.1311	0.1432	0.1447	0.1704
	2	0.0158	0.0189	0.0028	-0.0013	0.1305	0.1428	0.1447	0.1708
	3	0.0171	0.0218	0.0041	0.0022	0.1318	0.1432	0.1451	0.1704
1 & 2	1	0.0155	0.0180	0.0025	-0.0002	0.1167	0.1258	0.1280	0.1441
	2	0.0166	0.0194	0.0035	0.0014	0.1147	0.1220	0.1250	0.1390
1	1	0.0170	0.0184	0.0037	0.0023	0.0880	0.0916	0.0939	0.0993
True log hazard ratios: $\theta_1 = -0.2231$, $\theta_2 = -0.0953$, $\theta_3 = 0.3365$, $\theta_4 = 0.4055$									
All	1	0.0554	0.0696	0.0129	-0.0008	0.1612	0.1826	0.1632	0.1939
	2	0.0512	0.0649	0.0106	-0.0022	0.1563	0.1772	0.1582	0.1881
	3	0.0448	0.0565	0.0105	0.0005	0.1444	0.1618	0.1465	0.1711
	4	0.0444	0.0559	0.0107	0.0009	0.1425	0.1595	0.1445	0.1687
1, 2 & 3	1	0.0198	0.0239	0.0042	0.0001	0.1382	0.1515	0.1506	0.1758
	2	0.0175	0.0210	0.0022	-0.0024	0.1349	0.1476	0.1462	0.1700
	3	0.0165	0.0196	0.0039	0.0005	0.1254	0.1366	0.1356	0.1565
1 & 2	1	-0.0064	-0.0074	-0.0024	-0.0021	0.1180	0.1259	0.1278	0.1410
	2	-0.0040	-0.0046	-0.0001	0.0008	0.1144	0.1219	0.1236	0.1362
1	1	-0.0050	-0.0054	-0.0002	0.0003	0.0866	0.0893	0.0931	0.0972
True log hazard ratios: $\theta_1 = -0.4055$, $\theta_2 = -0.2231$, $\theta_3 = -0.0953$, $\theta_4 = 0$									
All	1	0.0070	0.0083	0.0005	-0.0020	0.1508	0.1676	0.1538	0.1736
	2	0.0077	0.0097	0.0017	0.0003	0.1462	0.1627	0.1489	0.1681
	3	0.0089	0.0099	0.0032	0.0008	0.1435	0.1602	0.1460	0.1652
	4	0.0063	0.0079	0.0007	-0.0009	0.1403	0.1562	0.1428	0.1613
1, 2 & 3	1	-0.0408	-0.0496	-0.0113	-0.0045	0.1405	0.1552	0.1498	0.1771
	2	-0.0381	-0.0457	-0.0089	-0.0009	0.1339	0.1483	0.1420	0.1677
	3	-0.0356	-0.0422	-0.0068	0.0022	0.1328	0.1464	0.1418	0.1673
1 & 2	1	-0.0437	-0.0483	-0.0137	-0.0069	0.1235	0.1321	0.1277	0.1411
	2	-0.0335	-0.0391	-0.0048	0.0002	0.1171	0.1244	0.1228	0.1349
1	1	-0.0249	-0.0269	-0.0046	-0.0027	0.0905	0.0945	0.0940	0.0993

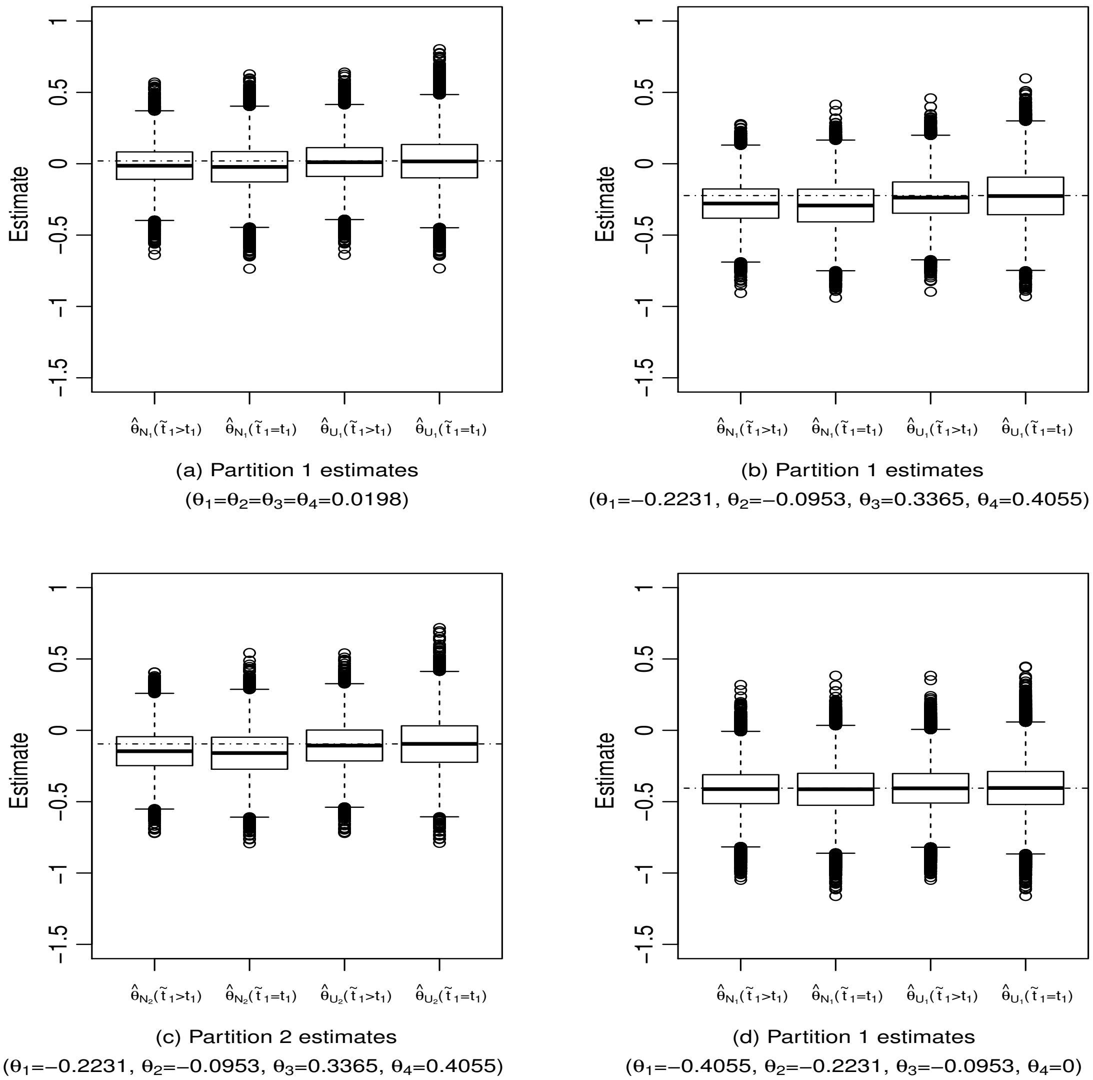

Figure S11: Boxplots for estimates in partition 1 (panels a, b and d) and partion 2 (panel c) when the full population is selected to continue to stage 2. Results are for Weibull ($\gamma = 0.5$) distribution when 200 events at interim analysis, 400 patients from stage 2 patients and an average of 40 additional events in selected partitions from the censored stage 1 observations. The horizontal dashed and dotted line in each plot corresponds to the true log hazard ratio of the partition considered in that plot while the true log hazard ratios in all partitions are given below the plot.

Table S17: Coverage probability and type I error rate (Weibull distribution, $\gamma = 0.5$)

True log hazard ratios	Selected partitions ($\mathcal{S}$)	Coverage (Type I error rate)[†]	
		Naive	Duality
$\theta_1 = \theta_2 = \theta_3 = \theta_4 = 0.0198$	All	95.1 (4.0)	98.5 (1.5)
	1, 2 & 3	95.7 (3.1)	99.0 (1.0)
	1 & 2	95.5 (3.1)	99.2 (0.8)
	1	95.5 (3.3)	99.3 (0.6)
$\theta_1 = -0.2231$, $\theta_2 = -0.0953$, $\theta_3 = 0.3365$, $\theta_4 = 0.4055$	All	94.4 (4.9)	98.6 (1.2)
	1, 2 & 3	95.8 (3.0)	99.1 (0.5)
	1 & 2	96.0 (1.8)	99.1 (0.0)
	1	96.1 (1.7)	99.3 (0.0)
$\theta_1 = -0.4055$, $\theta_2 = -0.2231$, $\theta_3 = -0.0953$, $\theta_4 = 0$	All	95.4 (2.6)	98.7 (0.7)
	1, 2 & 3	95.3 (1.0)	98.7 (0.0)
	1 & 2	94.7 (0.5)	98.0 (0.0)
	1	95.2 (1.0)	98.7 (0.0)

[†] Type I error is the probability that at least one upper bound is less than the true value.

15 Different selection rule: selecting a partition independent of the results in the other partitions

15.1 Simulation results for a different selection rule

To assess the characteristics of the various estimators when a different selection rule is used, we performed simulations for the case of continuing with any partition whose stage 1 log hazard ratio estimate is ≤ 0. The other aspects of the simulations are the same as those used to obtain the results in Table 4 in the main paper, where $\gamma = 0.5$, the number of events in each stage is 300, up to 2200 patients can be recruited for up to two years and three configurations for $(\theta_1, \theta_2, \theta_3, \theta_4)'$ are considered. The results for the point estimators for the three configurations of $(\theta_1, \theta_2, \theta_3, \theta_4)'$ which are $(0.0198, 0.0198, 0.0198, 0.0198)'$, $(-0.2231, -0.0953, 0.3364, 0.4055)'$ and $(-0.4055, -0.2231, -0.0953, 0)'$ are given in the supplementary material in Tables S18 to S20, respectively. The biases of the naive point estimator $\hat{\theta}_{N_j}$ are positive in all cases. This is because a partition is selected if it has a positive effect. In several scenarios, biases are larger than in the case of the adaptive threshold enrichment design (Results in Section 5.2 in the main paper). When $\tilde{t}_1 > t_1$, the UMVCUE $\hat{\theta}_{U_j}$ is slightly biased in some cases but has smaller RMSE than when $\tilde{t}_1 = t_1$. Hence we recommend having $\tilde{t}_1 > t_1$ and using $\hat{\theta}_{U_j}$ to obtain estimates.

The simultaneous properties of the naive and the duality confidence intervals are summarised in Table S21. For the two scenarios where the values for $(\theta_1, \theta_2, \theta_3, \theta_4)'$ are $(0.0198, 0.0198, 0.0198, 0.0198)'$ and $(-0.4055, -0.2231, -0.0953, 0)'$, unlike the naive confidence regions, the duality confidence regions have at least 95% coverage and the probabilities that at least one upper bound is less than the true value are less than 2.5%. For the other scenario of $(\theta_1, \theta_2, \theta_3, \theta_4)'$ equal to $(-0.2231, -0.0953, 0.3364, 0.4055)'$, the simulated probabilities (not reported in the table) for $\mathscr{S}$ equal to $\emptyset$, $\{1, 2\}$, $\{1\}$ and $\{2\}$ are 5.9%, 48.1%, 25.5% and 10.9%, respectively. In these cases which constitute more than 90%, the duality confidence regions have at least 95% coverage probability and the probabilities that at least one upper bound is less than the true value are less than 2.5%. For the remaining cases, the naive confidence intervals have undesirable properties because the coverage probabilities are as small as 88% and the probabilities that at least one upper bound is less than the true value are as high as 12%. The coverage probabilities for the simultaneous duality confidence intervals are generally at least the target 95% but the probabilities that at least one upper bound is less than the true value are mostly above 2.5%, although much smaller than those of the naive confidence intervals. We note that this is driven by the upper bounds for the treatment effects in Partitions 3 and 4. This may be considered to be of less practical impact since the log hazard ratios in these partitions are above 0 and the upper bounds are also mostly above 0 so that the new treatment would not be recommended in partitions 3 and 4. The reason that the duality upper bounds for the effects in partitions 3 and 4 do not show the desired properties is because the hypothesis testing described in Section 3.2 in the main paper does not control the type I error rate conditional on the selection made but controls the probability of selecting any partition where the treatment is not effective and concluding it is effective. When the treatment is effective in some partitions and not in others, conditional on the selection, the type I error rate is above the target 2.5%. Hence, since we assessed the properties of the simultaneous confidence intervals conditional on the selection made, tail probabilities for such scenarios can be above 2.5%.

15.2 Bigger treatment effects

To assess the properties of the estimators for the case of bigger treatment effects, we performed simulations when $(\theta_1, \theta_2, \theta_3, \theta_4)'$ is $(-1.0986, -0.9163, -0.6931, 0)'$, which corresponds to the hazard ratios vector $(1/3, 4/10, 1/2, 1)'$. We used the selection rule used in Section 5.3 in the main paper, that is, selecting all partitions that have positive stage 1 estimated effects. Unlike in other simulations, because the probabilities of selecting some subpopulations such as selecting partitions 1 and 2 are small, we simulated 1,000,000 trials. In most simulated trials, all partitions (49.97%) or partitions 1 to 3 (49.85%) were selected to continue to stage 2. Note that this means that partitions 1 to 3 are selected with a high probability (close to 1) and consequently the selection biases in these partitions would be expected to be neglible. The simulation results are given in Table S22 and Figure S12. Focusssing on the figure, which gives the Boxplots of the estimates in the different partitions when the full population is selected, for partitions 1 to 3, all estimators have approximately the same distribution which suggests neglible selection bias. For partition 3, all the estimators are practically mean unbiased. For partitions 1 and 2, all the estimators underestimate treatment effects with the underestimation bigger in partition 1 where the true log hazard ratio is largest. For partition 2 which corresponds to hazard ratio of 0.4, the bias is very small. We note that for partitions 1 and 2 most estimates are far from zero (point of no treatment effect) and so for an adequately powered trial, the underestimation is unlikely to lead to undesired clinical decision. Since the selection bias is negligible, and the biases increase with the true treatment effect, we attribute the underestimation to the asymptotic distribution of the score statistic being more accurate when the true log hazard ratio is small. In partition 4 where the control and the experimental treatment are equally effective, the naive estimators are positively biased but this is corrected adequately by the UMVCUE. So overall, the UMVCUE performs best in all partitions.

Table S18: Simulated biases and mean squared errors of the estimators for the log hazard ratios for the Weibull ($\gamma = 0.5$) distribution when $\theta_1 = \theta_2 = \theta_3 = \theta_4 = 0.0198$ (Configuration 1) and a partition is selected independent of the results in other partitions.

Selected partitions ($\mathcal{S}$)	Partition	Simulated bias				Root mean squared error			
		$\hat{\theta}_{N_j}$		$\hat{\theta}_{U_j}$		$\hat{\theta}_{N_j}$		$\hat{\theta}_{U_j}$	
		$\tilde{t}_1 > t_1$	$\tilde{t}_1 = t_1$	$\tilde{t}_1 > t_1$	$\tilde{t}_1 = t_1$	$\tilde{t}_1 > t_1$	$\tilde{t}_1 = t_1$	$\tilde{t}_1 > t_1$	$\tilde{t}_1 = t_1$
All	1	0.0787	0.0990	0.0275	0.0015	0.1469	0.1651	0.1529	0.1965
	2	0.0799	0.0993	0.0288	0.0009	0.1488	0.1689	0.1550	0.2034
	3	0.0769	0.0981	0.0251	-0.0012	0.1465	0.1669	0.1535	0.2018
	4	0.0782	0.1000	0.0267	0.0024	0.1467	0.1668	0.1530	0.1989
1, 2 and 3	1	0.0687	0.0838	0.0205	-0.0011	0.1401	0.1541	0.1474	0.1806
	2	0.0667	0.0812	0.0184	-0.0044	0.1365	0.1513	0.1433	0.1780
	3	0.0661	0.0827	0.0177	-0.0023	0.1357	0.1522	0.1430	0.1787
1, 2 and 4	1	0.0659	0.0823	0.0173	-0.0032	0.1384	0.1538	0.1465	0.1814
	2	0.0687	0.0850	0.0208	0.0010	0.1381	0.1524	0.1444	0.1759
	4	0.0679	0.0841	0.0198	-0.0005	0.1381	0.1537	0.1449	0.1789
1, 3 and 4	1	0.0693	0.0854	0.0214	0.0012	0.1392	0.1554	0.1459	0.1806
	3	0.0684	0.0846	0.0205	0.0006	0.1369	0.1523	0.1431	0.1760
	4	0.0710	0.0862	0.0234	0.0025	0.1412	0.1560	0.1476	0.1811
2, 3 and 4	2	0.0679	0.0823	0.0198	-0.0028	0.1385	0.1523	0.1457	0.1792
	3	0.0679	0.0839	0.0197	-0.0006	0.1376	0.1524	0.1445	0.1770
	4	0.0681	0.0834	0.0200	-0.0013	0.1382	0.1532	0.1452	0.1790
1 and 2	1	0.0584	0.0672	0.0171	0.0021	0.1249	0.1350	0.1295	0.1497
	2	0.0552	0.0669	0.0134	0.0016	0.1247	0.1366	0.1308	0.1523
1 and 3	1	0.0550	0.0651	0.0132	-0.0006	0.1234	0.1341	0.1292	0.1498
	3	0.0585	0.0667	0.0171	0.0014	0.1265	0.1360	0.1316	0.1516
1 and 4	1	0.0554	0.0662	0.0137	0.0008	0.1237	0.1357	0.1295	0.1516
	4	0.056	0.0668	0.0144	0.0016	0.1241	0.1356	0.1297	0.1508
2 and 3	2	0.0557	0.0658	0.0139	0.0002	0.1258	0.1367	0.1319	0.1532
	3	0.0542	0.0646	0.0121	-0.0014	0.1237	0.1357	0.1300	0.1524
2 and 4	2	0.0592	0.0692	0.0180	0.0045	0.1264	0.1374	0.1310	0.1517
	4	0.0532	0.0630	0.0110	-0.0034	0.1242	0.1350	0.1310	0.1526
3 and 4	3	0.0539	0.0643	0.0119	-0.0017	0.1227	0.1344	0.1288	0.1510
	4	0.0568	0.0650	0.0151	-0.0009	0.1272	0.1371	0.1333	0.1545
1	1	0.0363	0.0405	0.0067	0.0014	0.0993	0.1044	0.1028	0.1107
2	2	0.0360	0.0400	0.0065	0.0008	0.0988	0.1041	0.1023	0.1105
3	3	0.0357	0.0396	0.0062	0.0004	0.0991	0.1041	0.1027	0.1108
4	4	0.0375	0.0407	0.0081	0.0017	0.0996	0.1044	0.1027	0.1105

Table S19: Simulated biases and mean squared errors of the estimators for the log hazard ratios for the Weibull ($\gamma = 0.5$) distribution when $\theta_1 = -0.2231$, $\theta_2 = -0.0953$, $\theta_3 = 0.3365$ and $\theta_4 = 0.4055$ (Configuration 2) and a partition is selected independent of the results in other partitions.

Selected partitions ($\mathcal{S}$)	Partition	Simulated bias				Root mean squared error			
		$\hat{\theta}_{N_j}$		$\hat{\theta}_{U_j}$		$\hat{\theta}_{N_j}$		$\hat{\theta}_{U_j}$	
		$\tilde{t}_1 > t_1$	$\tilde{t}_1 = t_1$	$\tilde{t}_1 > t_1$	$\tilde{t}_1 = t_1$	$\tilde{t}_1 > t_1$	$\tilde{t}_1 = t_1$	$\tilde{t}_1 > t_1$	$\tilde{t}_1 = t_1$
All	1	0.0139	0.0221	-0.0123	-0.0266	0.1617	0.1723	0.1829	0.2218
	2	0.0313	0.0513	-0.0093	-0.0272	0.1364	0.1615	0.1561	0.2228
	3	0.1756	0.2162	0.0691	0.0016	0.2061	0.2446	0.1563	0.1967
	4	0.1872	0.2396	0.0632	-0.0104	0.2166	0.2676	0.1574	0.2152
1, 2 and 3	1	0.0269	0.0328	0.0066	-0.0008	0.1347	0.1466	0.1466	0.1725
	2	0.0472	0.0590	0.0139	0.0021	0.1380	0.1530	0.1501	0.1828
	3	0.1476	0.1811	0.0479	0.0018	0.1836	0.2139	0.1463	0.1764
1, 2 and 4	1	0.0227	0.0291	0.0018	-0.0051	0.1342	0.1436	0.1469	0.1702
	2	0.0466	0.0566	0.0131	-0.0011	0.1356	0.1485	0.1472	0.1781
	4	0.1699	0.2074	0.0574	0.0037	0.1999	0.2364	0.1462	0.1790
1, 3 and 4	1	0.0227	0.0221	0.0000	-0.0168	0.1564	0.1614	0.1716	0.1913
	3	0.1230	0.1646	0.0132	-0.0341	0.1560	0.1925	0.1240	0.1636
	4	0.1781	0.2074	0.0644	-0.0051	0.2045	0.2321	0.1441	0.1660
2, 3 and 4	2	0.0370	0.0288	0.0008	-0.0388	0.1362	0.1325	0.1529	0.1768
	3	0.1632	0.1699	0.0629	-0.0326	0.1958	0.2186	0.1539	0.2273
	4	0.1808	0.2216	0.0674	0.0151	0.2061	0.2444	0.1436	0.1657
1 and 2	1	0.0208	0.0244	0.0040	-0.0006	0.1215	0.1313	0.1304	0.1473
	2	0.0358	0.0422	0.0081	0.0001	0.1201	0.1299	0.1290	0.1475
1 and 3	1	0.0246	0.0298	0.0072	0.0037	0.1270	0.1367	0.1362	0.1531
	3	0.1218	0.1438	0.0337	0.0035	0.1578	0.1788	0.1271	0.1473
1 and 4	1	0.0183	0.0235	0.0003	-0.0037	0.1239	0.1338	0.1340	0.1521
	4	0.1388	0.1620	0.0381	0.0001	0.1702	0.1920	0.1274	0.1448
2 and 3	2	0.0406	0.0462	0.0114	0.0003	0.1295	0.1406	0.1396	0.1618
	3	0.127	0.145	0.0389	0.0021	0.1618	0.1805	0.1287	0.1496
2 and 4	2	0.0467	0.0535	0.0181	0.0083	0.1285	0.1420	0.1366	0.1607
	4	0.1375	0.1573	0.0352	-0.0093	0.1699	0.1883	0.1285	0.1470
3 and 4	3	0.1420	0.1487	0.0521	-0.0050	0.1739	0.1821	0.1336	0.1466
	4	0.1349	0.1652	0.0255	-0.0131	0.1643	0.1941	0.1196	0.1430
1	1	0.0126	0.0136	0.0013	-0.0008	0.0971	0.1020	0.1019	0.1089
2	2	0.0234	0.0257	0.0040	0.0004	0.0986	0.1034	0.1038	0.1113
3	3	0.0842	0.0921	0.0170	0.0007	0.1161	0.1246	0.0941	0.1026
4	4	0.0306	0.0405	-0.0473	-0.0662	0.0880	0.0961	0.1081	0.1278

Table S20: Simulated biases and mean squared errors of the estimators for the log hazard ratios for the Weibull ($\gamma = 0.5$) distribution when $\theta_1 = -0.4055$, $\theta_2 = -0.2231$, $\theta_3 = -0.0953$ and $\theta_4 = 0$ (Configuration 3) and a partition is selected independent of the results in other partitions.

		Simulated bias				Root mean squared error			
Selected		$\hat{\theta}_{N_j}$		$\hat{\theta}_{U_j}$		$\hat{\theta}_{N_j}$		$\hat{\theta}_{U_j}$	
partitions ($\mathbb{S}$)	Partition	$\tilde{t}_1 > t_1$	$\tilde{t}_1 = t_1$	$\tilde{t}_1 > t_1$	$\tilde{t}_1 = t_1$	$\tilde{t}_1 > t_1$	$\tilde{t}_1 = t_1$	$\tilde{t}_1 > t_1$	$\tilde{t}_1 = t_1$
All	1	0.0084	0.0099	0.0006	-0.0027	0.1461	0.1625	0.1540	0.1790
	2	0.0278	0.0358	0.0075	0.0005	0.1376	0.1520	0.1508	0.1839
	3	0.0505	0.0641	0.0165	0.0017	0.1370	0.1523	0.1498	0.1895
	4	0.0719	0.0900	0.0252	0.0013	0.1413	0.1588	0.1489	0.1925
1, 2 and 3	1	0.0067	0.0080	-0.0002	-0.0026	0.1362	0.1492	0.1429	0.1618
	2	0.0237	0.0289	0.0053	-0.0014	0.1309	0.1426	0.1423	0.1669
	3	0.0430	0.0523	0.0118	-0.0010	0.1285	0.1406	0.1400	0.1691
1, 2 and 4	1	0.0062	0.0075	-0.0009	-0.0034	0.1383	0.1527	0.1452	0.1658
	2	0.0257	0.0313	0.0074	0.0012	0.1307	0.1424	0.1420	0.1663
	4	0.0640	0.0782	0.0210	0.0032	0.1323	0.1475	0.1389	0.1713
1, 3 and 4	1	0.0084	0.0114	0.0014	0.0009	0.1376	0.1520	0.1444	0.1648
	3	0.0455	0.0547	0.0141	0.0009	0.1301	0.1420	0.1411	0.1700
	4	0.0608	0.0747	0.0169	-0.0022	0.1296	0.1437	0.1373	0.1696
2, 3 and 4	2	0.0278	0.0320	0.0094	0.0011	0.1326	0.1460	0.1439	0.1711
	3	0.0458	0.0550	0.0142	0.0007	0.1289	0.1409	0.1398	0.1702
	4	0.0651	0.0728	0.0213	-0.0063	0.1346	0.1448	0.1418	0.1734
1 and 2	1	0.0047	0.0057	-0.0010	-0.0023	0.1211	0.1304	0.1261	0.1384
	2	0.0198	0.0232	0.0046	0.0005	0.1173	0.1260	0.1259	0.1413
1 and 3	1	0.0057	0.0065	-0.0001	-0.0017	0.1233	0.1333	0.1285	0.1420
	3	0.0355	0.0418	0.0088	0.0010	0.1154	0.1240	0.1241	0.1414
1 and 4	1	0.0030	0.0052	-0.0029	-0.0032	0.1240	0.1352	0.1295	0.1441
	4	0.0511	0.0606	0.0134	0.0020	0.1196	0.1288	0.1258	0.1440
2 and 3	2	0.0182	0.0213	0.0022	-0.0028	0.1191	0.1277	0.1285	0.1452
	3	0.0399	0.0470	0.0134	0.0063	0.1167	0.1271	0.1245	0.1437
2 and 4	2	0.0288	0.0313	0.0134	0.0077	0.1268	0.1374	0.1351	0.1538
	4	0.0534	0.0595	0.0154	-0.0009	0.1218	0.1313	0.1276	0.1491
3 and 4	3	0.0457	0.0527	0.0192	0.0116	0.1183	0.1306	0.1249	0.1458
	4	0.0539	0.0648	0.0156	0.0046	0.1167	0.1279	0.1219	0.1432
1	1	0.0031	0.0036	-0.00064	-0.0011	0.0979	0.1028	0.1005	0.1063
2	2	0.0125	0.0140	0.0017	0.0000	0.0992	0.1016	0.1044	0.1087
3	3	0.0260	0.0295	0.0069	0.0044	0.0957	0.1004	0.1009	0.1081
4	4	-0.0782	-0.0762	-0.1083	-0.1167	0.1203	0.1223	0.1488	0.1614

Table S21: Coverage probability and type I error rate (Weibull distribution, $\gamma = 0.5$)

True log hazard ratios	Selected partitions ($\mathcal{S}$)	Coverage (Type I error rate)†	
		Naive	Duality
	All	94.6 (5.1)	97.9 (2.1)
$\theta_1 = \theta_2 = \theta_3 = \theta_4 = 0.0198$	1, 2 & 3	95.2 (4.6)	98.2 (1.8)
	1, 2 & 4	95.2 (4.6)	98.0 (2.0)
	1, 3 & 4	94.9 (4.8)	98.5 (1.5)
	2, 3 & 4	94.8 (4.8)	98.1 (1.9)
	1 & 2	95.4 (4.3)	98.5 (1.5)
	1 & 3	94.4 (5.1)	98.2 (1.8)
	1 & 4	94.9 (4.7)	98.2 (1.8)
	2 & 3	94.6 (4.9)	98.3 (1.7)
	2 & 4	94.9 (4.8)	98.1 (1.9)
	3 & 4	94.9 (4.6)	98.3 (1.7)
	1	95.0 (4.2)	98.8 (1.2)
	2	95.3 (4.0)	98.7 (1.3)
	3	94.7 (4.4)	99.2 (0.8)
	4	94.8 (4.3)	98.5 (1.4)
	All	93.0 (7.0)	96.5 (3.5)
$\theta_1 = -0.2231$, $\theta_2 = -0.0953$,	1, 2 & 3	91.5 (7.8)	95.9 (3.7)
$\theta_3 = 0.3365$, $\theta_4 = 0.4055$	1, 2 & 4	91.0 (8.5)	95.4 (4.3)
	1, 3 & 4	88.0 (12.0)	94.4 (5.6)
	2, 3 & 4	90.9 (9.1)	100.0 (0.0)
	1 & 2	95.6 (3.4)	99.6 (0.0)
	1 & 3	91.2 (8.3)	95.0 (4.6)
	1 & 4	89.0 (10.5)	92.8 (6.5)
	2 & 3	91.5 (8.0)	96.4 (3.3)
	2 & 4	87.6 (11.8)	94.0 (5.4)
	3 & 4	95.7 (4.3)	100.0 (0.0)
	1	95.7 (2.8)	99.5 (0.0)
	2	95.5 (3.4)	99.9 (0.0)
	3	89.2 (10.8)	95.4 (4.6)
	4	87.8 (12.2)	94.8 (5.2)
	All	95.9 (3.4)	98.2 (1.6)
$\theta_1 = -0.4055$, $\theta_2 = -0.2231$,	1, 2 & 3	96.0 (2.9)	99.7 (0.0)
$\theta_3 = -0.0953$, $\theta_4 = 0$	1, 2 & 4	95.6 (3.4)	98.1 (1.6)
	1, 3 & 4	96.1 (3.1)	98.5 (1.2)
	2, 3 & 4	95.2 (4.2)	98.8 (1.2)
	1 & 2	95.7 (2.7)	99.6 (0.0)
	1 & 3	96.0 (2.9)	99.7 (0.0)
	1 & 4	95.5 (3.5)	98.1 (1.5)
	2 & 3	94.7 (4.5)	99.8 (0.0)
	2 & 4	95.1 (3.8)	97.8 (1.9)
	3 & 4	97.6 (2.0)	99.2 (0.4)
	1	94.8 (2.5)	99.3 (0.0)
	2	94.0 (4.4)	99.9 (0.0)
	3	94.0 (4.7)	99.7 (0.0)
	4	94.4 (4.8)	98.4 (1.6)

† Type I error is the probability that at least one upper bound is less than the true value.

16 Point estimation with bigger treatment effects

Table S22: Simulated biases and root mean squared errors when $\theta_1 = -1.0986$, $\theta_2 = -0.9163$, $\theta_3 = -0.6931$ and $\theta_4 = 0$ (Weibull distribution, $\gamma = 0.5$)

Selected partitions ($\mathcal{S}$)	Partition	Simulated bias				Root mean squared error			
		$\hat{\theta}_{N_j}$		$\hat{\theta}_{U_j}$		$\hat{\theta}_{N_j}$		$\hat{\theta}_{U_j}$	
		$\tilde{t}_1 > t_1$	$\tilde{t}_1 = t_1$	$\tilde{t}_1 > t_1$	$\tilde{t}_1 = t_1$	$\tilde{t}_1 > t_1$	$\tilde{t}_1 = t_1$	$\tilde{t}_1 > t_1$	$\tilde{t}_1 = t_1$
All	1	-0.0463	-0.0586	-0.0463	-0.0586	0.1574	0.1753	0.1574	0.1753
	2	-0.0245	-0.0327	-0.0246	-0.0327	0.1511	0.1682	0.1512	0.1684
	3	-0.0086	-0.0124	-0.0092	-0.0131	0.1461	0.1627	0.1470	0.1642
	4	0.0658	0.0825	0.0221	0.0000	0.1308	0.1465	0.1384	0.1783
1, 2 and 3	1	-0.0460	-0.0575	-0.0461	-0.0575	0.1452	0.1603	0.1453	0.1603
	2	-0.0249	-0.0324	-0.0249	-0.0324	0.1389	0.1527	0.1390	0.1528
	3	-0.0090	-0.0126	-0.0095	-0.0132	0.1338	0.1470	0.1345	0.1481
1, 2 and 4	1	-0.0486	-0.0612	-0.0486	-0.0613	0.1477	0.1659	0.1478	0.1659
	2	-0.0193	-0.0256	-0.0193	-0.0257	0.1471	0.1617	0.1472	0.1619
	4	0.0612	0.0720	0.0212	0.0012	0.1246	0.1383	0.1304	0.1620
1, 3 and 4	1	Selected 47 times only							
	3								
	4								
2, 3 and 4	2	Selected 3 times only							
	3								
	4								
1 and 2	1	-0.0500	-0.0592	-0.0500	-0.0592	0.1299	0.1425	0.1299	0.1425
	2	-0.0229	-0.0318	-0.0229	-0.0318	0.1175	0.1286	0.1176	0.1286
1 and 3	1	Selected 63 times only							
	3								
1 and 4	1	Not selected							
	4								
2 and 3	2	Selected 6 times only							
	3								
2 and 4	2	Not selected							
	4								
3 and 4	3	Not selected							
	4								
1	1	Not selected							
2	2	Not selected							
3	3	Not selected							
4	4	Not selected							

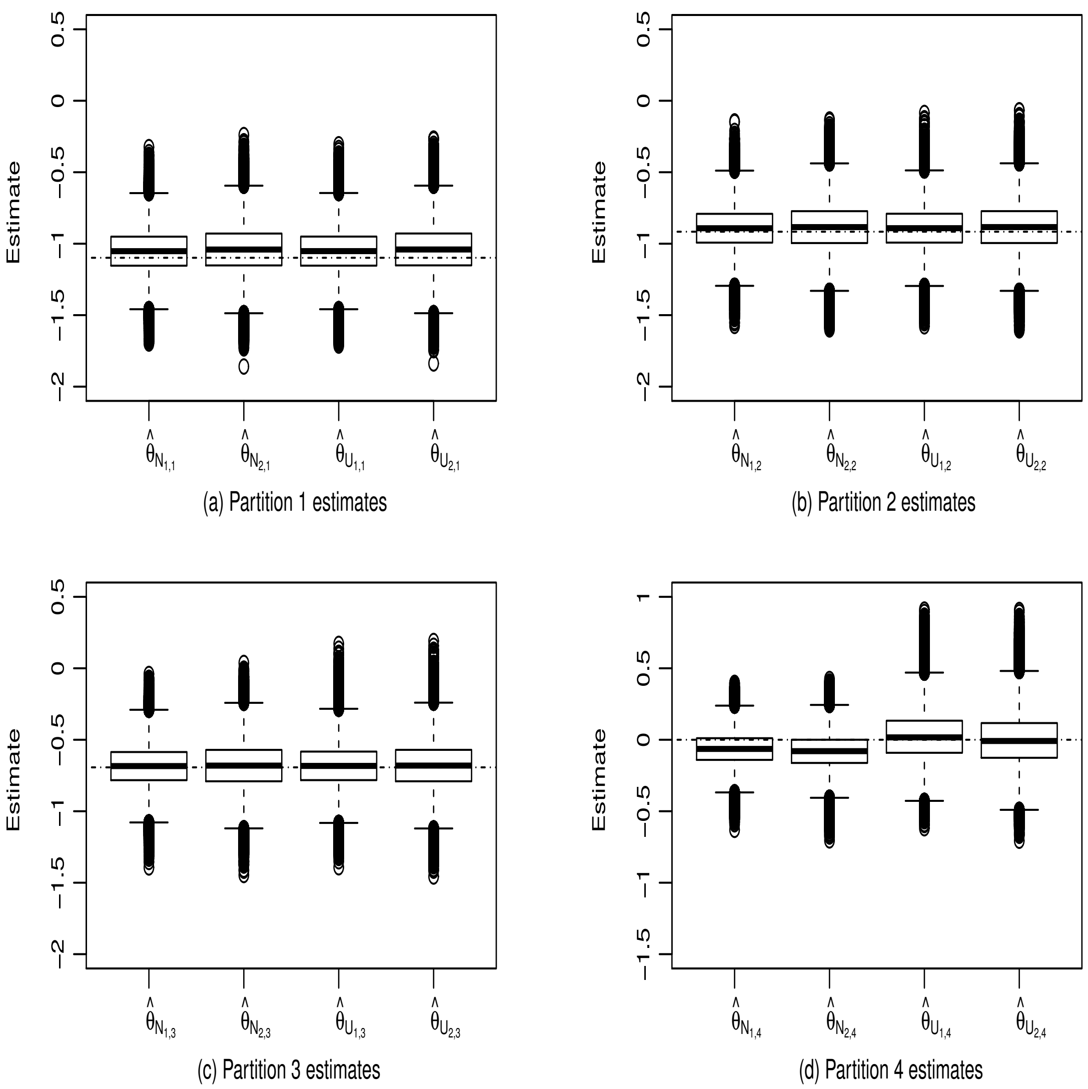

Figure S12: Boxplots for the estimates in different partitions when the full population is selected to continue to stage 2 when $\theta_1 = -1.0986$, $\theta_2 = -0.9163$, $\theta_3 = -0.6931$ and $\theta_4 = 0$ with the Weibull ($\gamma = 0.5$) distribution. The horizontal dashed and dotted line in each plot corresponds to the true log hazard ratio in the partition.

17 Confidence intervals with bigger treatment effects

Table S23: Coverage probability and type I error rate (Weibull distribution, $\gamma = 0.5$)

True log hazard ratios	Selected partitions ($\mathcal{S}$)	Coverage (Type I error rate)†	
		Naive	Duality
	All	95.3 (1.7)	98.7 (0.4)
$\theta_1 = -1.0986$, $\theta_2 = -0.9163$,	1, 2 & 3	95.1 (1.2)	98.8 (0.0)
$\theta_3 = -0.6931$, $\theta_4 = -0.2231$	1, 2 & 4	95.4 (1.5)*	100 (0.0)*
	1, 3 & 4	Selected 9 times	
	2, 3 & 4	‡	‡
	1 & 2	Selected 28 times	
	1 & 3	Selected once	
	1 & 4	‡	‡
	2 & 3	‡	‡
	2 & 4	‡	‡
	3 & 4	‡	‡
	1	‡	‡
	2	‡	‡
	3	‡	‡
	4	‡	‡

† Type I error is the probability that at least one upper bound is less than the true value.
* Results based on 130 simulated trials; ‡ Not selected.

References

[1] R Development Core Team. *R: A Language and Environment for Statistical Computing*. R Foundation for Statistical Computing, Vienna, Austria, 2010. URL `http://www.R-project.org/`. ISBN 3-900051-07-0.

[2] Arthur Cohen and Harold B. Sackrowitz. Two stage conditionally unbiased estimators of the selected mean. *Statistics and Probability Letters*, **8**:273 – 278, 1989.

[3] Jack Bowden and Ekkehard Glimm. Unbiased estimation of selected treatment means in two-stage trials. *Biometrical Journal*, **50**(4):515 – 527, 2008.

[4] Peter K Kimani, Susan Todd, and Nigel Stallard. Conditionally unbiased estimation in phase II/III clinical trials with early stopping for futility. *Statistics in Medicine*, **32**(17):2893–2910, July 2013.

[5] D S Robertson, A T Prevost, and J Bowden. Accounting for selection and correlation in the analysis of two-stage genome-wide association studies. *Biostatistics*, **17**(4):634–649, 2016.

[6] D S Robertson, A T Prevost, and J Bowden. Unbiased estimation in seamless phase II/III trials with unequal treatment effect variances and hypothesis-driven selection rules. *Statistics in Medicine*, **35**(22):3907–3922, 2016.

[7] Peter K Kimani, Susan Todd, and Nigel Stallard. Estimation after subpopulation selection in adaptive seamless trials. *Statistics in Medicine*, **34**:2581–2601, 2015.

[8] Peter K Kimani, Susan Todd, Lindsay A Renfro, and Nigel Stallard. Point estimation following two-stage adaptive threshold enrichment clinical trials. *Statistics in Medicine*, **37**:3179–3196, 2018.

[9] Susan Todd, John Whitehead, and Karen M Facey. Point and interval estimation following a sequential clinical trial. *Biometrika*, **83**(2):453 – 461, 1996.

[10] Kevin Kunzmann, Laura Benner, and Meinhard Kieser. Point estimation in adaptive enrichment designs. *Statistics in Medicine*, **36**(25):3935–3947, 2017.
